# The Essays, Cases, Intelligence, &c.: D—E

**Published:** 1820

**Authors:** 


DAISY, *(bellis perennis)*, botanical description of the, xix. 456.

DALBY'S CARMINATIVE, fatal effects of that remedy on children, v. 39; pernicious to adults, 40.

DALLAM, W. B. on the efficacy of cotton applied to burns, xxix. 137.

DALRYMPLE, William, his account of a case of trismus cured by cold affusion, xiv. 267.

DALTON, Mr. on the supposed origin of the cow-pox in the grease of horses, xvi. 315; his experiments with variolous matter on cows, 316; on the combination of hydrogen and carbon, xxix. 516; observations on his theory, xxx. 81.

DAMART, M. method discovered by him of purifying vegetable oil, viii. 383.

DAMP, observations on the supposed injurious effects of, xvii. 54; cloth slippers recommended to prevent the effects of, xx. 477; cerecloth shirt a good preservative from, 563; on that of mines, xxviii. 328; Dr. Clanney's invention for preventing it in mines, xxx. 82; dreadful effects of one in Collingwood Main Colliery, Northumberland, 427; institution for preventing it in mines, xxxi. 341.

DAMPIER, William, the Voyager, his remedy for rabies canina, xx. 271.

———, Henry, his case of intestinal protrusion per anum, xxxi. 164.

DANCER, Dr. Thomas, his account of a negro turning white, viii. 97; observations by him on the contagious nature of yellow fever, xiv. 385; letter to him from Dr. Brown on the same subject, 389; explanation in reply to, xv. 560; his critical observations on Dr. Grant's Latin treatise on yellow fever, xvi. 410; reply to his opinions on contagion, xvii. 505.

DANCING, remarkable practice of it among the Aborigines of Cuba, xxv. 480.

DANDELION, *(leontodon officinalis,)* the juice of it beneficial in cutaneous affections, xix. 72; botanical description of the, 166; its diuretic qualities, 167; an antiscorbutic, ibid.; its nutritious virtue, ibid.

DAPHNE ALPINA, experiments on the bark of the, xxix. 519.

DAPHNE LAUREOLA, or spurge laurel, description of the, xiv. 545; beneficial in rheumatism, ibid.; an excellent vermifuge, ibid.; its active qualities, xxi. 13.

DAPHNE MEZEREUM, the basis of many quack medicines, i. 158; medicinal uses of its bark, v. 278; botanical description of the, xiv. 543; beneficial in ulcers, 544; and in syphilis, ibid.; its corrosive qualities, 545; danger of it, ibid.; obscurity of its operation, xxi. 11.

DARAN, M. observations on his method of removing strictures, xxi. 373.

DARBEY, Dr. his recommendation of musk in sphacelus, ii. 13; observations on his claim to that practice, 342.

DARLINGTON, (Durham,) a sulphuric spring discovered at, xiv. 191.

DARNEL, (*lolium*,) its deleterious effects in bread, i. 423; on the different species of it, ii. 105; description of it, xi. 529; on its intoxicating qualities, ibid.; fatal instances of using it by mistake in flour, xxviii. 186.

DARRACQ, M. his experiments on the reciprocal action of the earths, viii. 474.

DARTMOOR, remarkable instance of mortality in a family resident on, xxviii. 23.

DARTMOUTH COLLEGE, (New Hampshire,) account of a remarkable bilious fever at, ii. 568.

DARTON, William, on the efficacy of the yellow gum of Botany Bay, vi. 530.

DARTRE, description of two species of eruptions under that name, with the efficacy of hepar sulphuris as a remedy, xxxii. 165; farther observations on the same, xxxiv. 257.

DARWIN, Dr. Charles, vindication of his experiments on pus and mucus, iii. 374; his experiments to establish a criterion between mucilaginous and purulent matter, iv. 49, 108, 203; his recommendation of salted meat as a remedy in corpulency, xxv. 451; his observations on the urine and blood in diabetes, xxvi. 465; on the action of the absorbents, 467; his theory of the regurgitation of the fluids in the absorbent vessels, xxxii. 43.

———, Dr. Erasmus, his early adoption of the Brunonian doctrine, i. 125; his observations on pestilential air, ii. 181; his peculiar opinions on sensorial power, 277; on the spirit of theorizing, 422; his observations on fever, 424; on matter produced by suppuration, 427; on the causes of hydrocephalus, iii. 117; observations on his opinions, iv. 238; defence of his Zoonomia, 139; character of that work, 183; remarks on his doctrine of fever, v. 388, 577; on his theory of animal impregnation, vi. 254; anticipation of his poem on the loves of the plants, 415; his opinion on the stimulating power of opium, vii. 126; on tympany, 234; on apoplexy, 363; confirmation of his opinion on the suspension of diseases, viii. 31; his advice to gouty patients, 355; on his adhesive plaster for ulcers, ix. 116; his suggestion respecting the printing of medical works, x. 337; on the treatment of convulsions, xv. 53; on the efficacy of digitalis in anasarca, xvi. 354; his doubts on the virtue of that plant in hydrothorax, 356; his observations on the salutary effects of cold as a remedy in diseases, 368; remarks on his theory of fever, xvii. 448; his treatment of the gout, xviii. 168; his peculiar opinions on consumption, xix. 265; observations by him on sea-sickness, 297; his doubts on the efficacy of digitalis, xx. 525; on the four kinds of temperaments, xxi. 199; animadversions on that opinion, 211; on the treatment of the scirrhous contracted rectum, xxii. 287; on his theory of the retrograde action of the absorbents, xxiii. 332; commencement of his acquaintance with Dr. Beddoes, xxv. 174; his case of the efficacy of tobacco in hæmorrhage of the rectum, 225; successful imitation of his style, 260; his correspondence with Dr. Beddoes, 262; on the character of his Zoonomia, 264; objections to his theory of sensation, xxvi. 284; on a French translation of his poem, 296; excellence of his practical rules, xxvii. 103; his Zoonomia severely criticised, xxviii. 324; his comparison of hydrophobia and tetanus, 356; observations on his case of tetanus, xxix. 273; his opinion on theorizing, xxx. 109; his method of treating typhus, xxxii. 183.

DARWIN, Dr. (of Shrewsbury,) his treatment of the inflexibility of the articulations, xviii. 6.

DASHWOOD, Charles, his case of the efficacy of cold affusion in tetanus, xv. 496.

DATISCA CANNIBA, its virtues in intermittents, i. 188; method of administering it, 189; its effects on the digestive organs, 190.

DATURA STRAMONIUM, (thorn-apple,) its singular effects as a stimulant, i. 84; farther observations on the medicinal properties of the, 278; an extract prepared from its seeds, ibid.; its diuretic qualities, ibid.; its effects on the nervous system, 279; successful in phrenitis, ibid.; superior to opium as a narcotic, iii. 438; botanical description of, xii. 131; different species of the, xxv. 380; long in use among the Chinese as a narcotic, 383; on its revival as a medicine, xxvi. 21; used for nefarious purposes in the east, 22; caution on its use, 23; description of the datura metel, xxvii. 324; analysis of it, xxviii. 71.—See *Stramonium*.

DAUBENTON, M. his observations on indigestion, xvi. 569; on the aliments proper for human food, xxi. 512.

DAUCUS, (wild carrot), botanical description of the, xii. 231; its efficacy in calculous complaints, 232; externally applied in cancerous sores, ibid.; beneficial in the gravel, ibid.

DAVIDGE, Dr. on the autumnal epidemic of America, i. 105.

DAVID'S, (St.) observations on the country about that promontory, xxviii. 236.

———, Mr. his introduction of vaccination in Holland, vii. 252.

DAVIDSON, James, description of his improved forceps for midwifery, xxvi. 510.

———, Dr. George, his experiments with the eudiometer in the West Indies, ii. 386; observations by him on the yellow fever, ix. 100.

———, William, on the beneficial effects of abstinence in consumption, i. 20; remarks on his opinion, 21.

DAVIE, L. on the advantage of emetics in ague, xii. 499; on the benefit of uva ursi in consumption, xv. 346.

DAVIES, Rev. Mr. description of his portable bed for the conveyance of drowned persons, xxiii. 338.

———, Dr. of Carmarthen, his observations on the influenza, x. 103.

———, D. H. on the good effects of calomel in a case of obstinate vomiting, xxv. 308; on the benefit of calomel and opium in rheumatism, xxvi. 210; farther instances of the same, 277; remarks on his practice, 440; his case of poison by arsenic, xxviii. 344; remarks on his case, 405, *note*; on the action of mineral poisons in the circulation, xxix. 133; his account of a remarkable tumour of the nose, 391; on the efficacy of mercurial action in diseases, xxx. 446.

———, Henry, on a case of adhesion of the placenta after delivery, iii. 6; observations on his case, 221; his reply and explanation, 333; Dr. Squire's remarks on his case, 459, 464; observations on the elucidation of his case, 516; his answer to Dr. Squire, 536; farther remarks on his case by Dr. Syer, iv. 4; his observations on the doses of medicine, 160; his case of hydrothorax, 384; his case of mortification from the use of cold water, v. 534; his cases of apoplexy, vii. 74; observations by him on the controversy in regard to the cause of apoplexy, 259; remarks on his cases, 400; his case of pertussis cured by the solution of bark, 506; on the treatment of tinea capitis, ix. 60; on the benefit of opium in rheumatism, xvi. 495; his form of a liniment for that complaint, 496; his observations on medical controversies, xviii. 256; answers to him on that subject, 315, 420; remarks on his method of treating rheumatism, 430; his answer to Mr. Ring on the vaccine contest, 537; reply to him on the same, xix. 53; his observations on obstetric cases, xxiii. 18; remarks on his cases, 290, 461; his case of spontaneous ptyalism without mercury, xxvi. 452; observations on the case, xxvii. 276.

DAVIES, Hugh, his account of native plants in the Isle of Anglesea, xxxiii. 409.

———, Robert, on the efficacy of cerussa acetata in hæmorrhage, xix. 8; on the benefit of the same in chronic mænorrhagia, 11.

———, Mr. of Bishop's-castle, his observations on the influenza, x. 105.

———, T. L O. his case of puncture of the urinary bladder, xiii. 10; his case of bronchocele, 13.

DAVIESIA LATIFOLIA, an elegant shrub of New Holland described, xxvi. 512.

DAVIS, Dr. George, his observations on the Barbary coast, x. 381.

———, Dr. J. B. his cases and remarks on hydrocephalus, viii. 98; on hepatic diseases, 238; on a hospital for incurables, xvii. 362; account of his history of Nice, xviii. 385; his account of the fever among the troops in the Walcheren expedition, xxiii. 418.

———, Dr. J. F. his remarkable case of petechiæ, i. 420; on the different species of darnel, ii. 105; on vaccine inoculation, 106; his complaint against Mr. Cuff, viii. 96; his conduct animadverted on with regard to vaccination, ix. 66.

———, Timothy, his observations on rabies canina, xvi. 437.

DAVY, Sir Humphrey, his experiments on the excrements of fowls, i. 505; account of his chemical and philosophical researches, iv. 263, 360; his intrepidity in experiment on respiring hydrocarbonate, 361, 451; his invention of the eudiometer, vi. 10; his galvanic experiments, 214; discovers an acid in a new fossil, xvi. 191; his observations on some chemical effects of electricity, xviii. 93; his new theory of fairy rings, 197; on the influence of electricity in the mineral kingdom, 198; his discovery of the decomposition of the alkalies, xix. 93, 191; on some important chemical agencies of electricity, 190; his experiments repeated and confirmed, 286; farther particulars of his grand discovery of the decomposition of the alkalies, xx. 95; his experiments on ammonia, 96; his observations on the earths, 97; description of his galvanic battery, 254; on the decomposition of the earths, 383; his experiments verified in France, 568; observations on the importance of his discovery, xxi. 29; vindication of his claims as an original discoverer in chemistry, 129; first brought forward by Dr. Beddoes, 189; his experiment in decomposing potash, 264; his method of charging the galvanic battery for the decomposition of the alkalies, 350; improvements made in his apparatus, 527; summary view of his discoveries, xxii. 11, 14; his definition of galvanism, 323; continuance of his chemical pursuits, xxiv. 36; his observations on muriatic acid, 345; on the combinations of oxymuriatic gas and oxygen with the metals, xxv. 83; his method of operating on the metals, ibid.; his experiments on the oxymuriatic gas, 180; proposes to call that gas chlorice, ibid.; his observations on the air in the lungs, ibid.; original anecdotes of him, 264; on the gaseous combination of oxymuriatic gas and oxygen, 361; gives the name of zuthine or zuthic gas to that compound, 362; on the conversion of the alkalies into metallic substances, 363; his account of a meteoric stone found in Ireland, 545; derives those bodies from planetary ruins, 546; his indefatigable diligence, xxvi. 18, 19; advantageous application of his discoveries to manufactories, ibid.; prize question on his discoveries, 168; his paper on the combination of the oxymuriatic gas and oxygen gas, 298; observations on his experiments, xxvii. 15; on combinations of phosphorus and sulpher, xxviii. 157; his experiments on sulphuric acid, ibid.; observations on a passage in his elements of chemical philosophy, 357; his theory of heat supported by experiments, 512; account of his Elements of Agricultural Chemistry, xxx. 13; observations on his number of metallic sulphurets, 81; on the combination of phosphorus with chlorine, 83; his observations on a new detonating compound of chlorine and azote, 424; on the composition of the fluor spar and its acid, 425; his account of a new gas from carbonate of soda, xxxi. 235; his farther experiments on fluorine, 337; his observations on silica, ibid.; on chlorine, 338; his experiments and observations on iodine, xxxiii. 37; on the combustion of the diamond and other carbonaceous substances, 121; on the action of acids on hyper-oxymuriate of potash, xxxiv. 79.

DAVY, Dr. John, his observations on explosions in the combination of the gases, xxvi. 298; on the action of oxide of carbon and oxymuriatic gas when exposed to the sun, xxvii. 340; on the combinations of metals with chlorine and oxygen, ibid.; his experiments on silicated fluoric gas, xxviii. 157; on the gaseous compound of carbonic oxide, and chlorine, 195; on animal heat, 339; his opinion on the extraction of caloric from the blood, xxxii. 223; his experiments on animal heat, xxxiii. 113.

DAWES, John, his observations on the economy of the liver, xviii. 293; remarks on the physiological errors contained in his paper, 426.

———, Rev. Mr. his account of the plague at Aleppo, vi. 365.

DAWLISH, Devonshire, meteorological observations at, xxx. 131, 133, 134.

DAWSON, G. P. his case of procidentia uteri, xiii. 129; on the use of mercury in venereal affections, 549; approbation of his plan of treating the prolapsus uteri, xiv. 98; on a singular case of delivery, 155; on recent depressions of the scull, 161; reply to his observations on a case of delivery, 242, 246, 417; his case of incipient procidentia uteri, xv. 21; observations on the value of his communications, 235; on a case of diseased bladder, xvi. 253; replies to him on the case, xvii. 35, 39; his answers to Mr. Thomas, 258; farther reply to him, 527; his concluding observations on the case, xix. 13.

———, James, his case of popliteal aneurism, xviii. 475; his exposure of the statement of Dr. Carson on a case of poison, xxi. 336, 431; on his practice of vaccination, xxv. 308

———, Dr. on the use of opiates in hydrocephalus, ii. 255.

DAY, on the relation between some animal functions and the time of the, xxxiii. 225.

———, Dr. a remarkable empiric in London, original anecdotes of him, xii. 137.

DAZILLE, Dr. on the effects of opium in tetanus, xxvi. 404.

DEACON, Dr. letters from Dr. Mead to, x. 48.

DEAD BODIES, effects of arsenic in preserving them from putrefaction, xi. 192; farther observations on the same, xvi. 95; on the changes produced in them, 302; on the contagious effects of their effluvia, xxiv. 422.

DEAFNESS, observations on that of blacksmiths, vii. 423; effects of galvanism in the cure of, viii. 255, 324, 526; method of applying galvanism in cases of, ix 133, 142, 145, 290; x. 59; 61, 333; caution in the treatment of infants supposed to be affected with, xiii. 451; efficacy of garlic in, xiv. 65; on the application of galvanism in congenital, xvi. 472; various causes of, xviii. 5; cured by perforation of the tympanum, xxii. 191; successful treatment of a case of, xxvii. 377; on the claim of Dr. Wallis to the invention of teaching the deaf and dumb to speak, xxviii. 193, 194; cured by a simple process, xxxi. 279; cured by puncturing the tympanum, 346; case successfully treated by the removal of a splinter of wood from the meatus externus, xxxiii. 226.

DEAN, Mr. his case of the cure of uterine cancer, viii. 432.

DEATH, on the use of vomits in apparent, i. 59; means of relief in sudden danger of, ii. 497; remarkable case of the recovery of an infant in a state of apparent, iii. 223; physiological researches on life and, v. 476; observations on natural, 479; influence of the death of the heart on the brain, 563; on that of the lungs, 565; on that of the brain, ibid.; directions for the recovery of persons in a state of apparent, vi. 83, 318; on the cause of the arteries being found empty after, 379; effects of galvanism in apparent, viii. 256; danger of premature interment in cases of apparent, ix. 73; principal signs of violent, 73, 75; advice in cases of it by suspension, 76; case of sudden, from a morbid affection of the brain, xii. 391; galvanism a certain criterion of, xiii. 86; case of resuscitation in supposed, xiv. 7; machine invented to distinguish real from apparent, xv. 295; on the changes produced in the body by, xvi. 302; case of sudden death by the inspiration of nitrous gas, xvii. 188; case of a man who died of a mild punishment, xviii. 176; case of transudation after, xxii. 31; remarks in consequence, 32; on the opinion of Bichat with respect to life and, 254; on the digestion of the stomach after, xxiii. 399; observations on violent, 407, 428; case of diseased action producing sudden, xxiv. 178; analogy between that of animals and vegetables, xxvi. 8; how produced by vegetable poisons, 129; case of sudden, xxvii. 179; on that caused by the wind of a ball, xxix. 63; xxx. 246; on the appearance of the stomach after, xxxi. 177; case of it suddenly after delivery, xxxii. 135; on the mode by which it is produced by hanging, 469.

DEATHS, annual list of mortality in London for 1799, i. 336; ii. 320; iii. 111; for 1800, iv. 474; v. 302; for 1801, vii. 112; at Portsmouth, in New Hampshire, in 1801, xii. 94; at the same place in 1802, 190; at Vienna, xiv. 382; in London, during 1805, xv. 205; in the same for 1806, xvii. 96; in Russia for 1806, xviii. 193; in London for 1807, xix. 96; for 1808, xxi. 87; in New Hampshire, 523; in London for 1809, xxiii. 87; at Portsmouth, in New Hampshire, xxvi. 127; proportion of them in child-bed to births, xxv. 213; on the number of, in Saxony, 244; by the small-pox in London for the twelve years preceding vaccination, xxvi. 509; by the small-pox in 1813, xxix. 525; xxx. 522; annual report of them in different counties of Great Britain for 1811, xxx. 140; within the bills of mortality for 1813, xxxi. 84, 258, 347, 434, 522; the same for 1814, xxxii. 82, 170; report in London for 1814, xxxiii. 165; comparison of the number of them from measles at Manchester, 419.

DEBAUCHERY, case of inflammation of the kidneys and bladder occasioned by, xxxi. 371.

DEBECK, Dr. his method of expelling the tape-worm, xi. 434.

DEBILITY, account of the Brunonian theory of, i. 127; on the causes of it in the human system, 202; on direct and indirect, iii. 49; observations on cases of, v. 119; on that produced by venereal complaints, viii. 8; on the defect of sensibility in diseases of, 89; benefit of the common masterwort in disorders of, xii. 371; a defect in the system rather than the cause of disease, xiii. 179; efficacy of muriate of lime in, xiv. 80; remarkable instance of nervous, 477; caused by profuse perspiration, xvi. 80; objections to the Brunonian doctrine of, xviii. 464; method of treating a case of general, 478; benefit of the cold bath in cases of, xix. 112, 304; on the stimulant effect of excessive, xx. 395; the diabetes not owing to, 547; not a predisposing cause of dysentery, xxi. 18; observations on that of the female organs, 31; the dropsy after fever not the effects of, xxii. 300; follows an impeded pulmonary transmission, xxiii. 117; the decay in trees analogous to that of the human frame, xxvi. 8; objections to the Brunonian theory of, 36; method of treating diseases of, xxvii. 145; case of transparent mucous discharge depending on, xxxii. 342.

DECANDOLLE, M. his description of the senniebria pinnatifida, i. 304; his observations on the physiology of plants, xx. 16; his account of a new species of aloes, xxv. 370.

DECAPITATION, on supposed sensations of the head in, i. 51; effects of galvanism on a body after, x. 576.

DE CARRO, Dr. his zeal in the cause of vaccination at Vienna, v. 157; his letter to Dr. Jenner narrating the success of that discovery in Germany and Italy, 351; communication from Dr. Jenner to him on the inoculation of dogs, vi. 95; the founder of vaccination on the Continent, vii. 110; his observations and experiments on the cow-pox, 185; his account of the progress of vaccination in Austria and the East, viii. 193; his method of transmitting vaccine virus, ix. 128; his letter to Dr. Jenner on the progress of vaccination in the East, 450; his letter to Dr. Marcet on the success of vaccination at Vienna, x. 242; his account of that practice at Constantinople, 474; his narrative of the progress of it in India, xii. 122; account of his history of vaccination, 447; his remarks on Mr. Goldson's pamphlet, 462; letter from Dr. Christie to him on the state of vaccination in Ceylon, xiii. 122; letter from the hon. Governor North to, 128; on the opponents of vaccination, 249; on the language of controversy, 250; his account of the effects of vaccine inoculation in the case of herpetic eruptions, ibid.; sends vaccine virus to America, xiv. 283; on the insusceptibility of children to the small-pox, 408; his exertions in promoting vaccination, xv. 390; writes the history of that discovery, 516; on the opponents of vaccination, xvi. 320; letter to him on the grease in Arabian horses, 322; on the guinea-worm and the stings of scorpions, xvii. 167, 173; his case of plica polonica, xxvi. 388; his zeal for vaccination, xxix. 158.

DECOMPOSITION, that of oxygen by carbon and hydrogen, i. 168; on that of calcareous muriate of lime, 194; on that of vegetable bodies, 264; on that of sea-salt and sal ammoniac, 301; on that of muriatic acid, ii. 78; acidulated phosphat of lime obtained from that of bones, 79; on that of the oxyd of tungsten, iii. 87; on that of the carburet of lime by phosphorus, 374; on that of water by electricity, iv. 122; that of red lead in nitric acid, 374; that of the nitrous acid, vi. 12; of the sulphat of barytes, 467; of the muriatic acid, xv. 294; the spontaneous decomposition of animal substances, xvi. 103; discovery of that of the alkalies, xix. 93, 101; xx. 95, 96, 383; confirmation of that discovery in France, 568; on the importance of that discovery, xxi. 29; summary view of the discovery, xxii. 11, 14; account of that of the fluoric and muriatic acids, xxiii. 292; of the fulminating powders of mercury and silver by charcoal, xxv. 457; of vegetable or animal substances by heat, 508; question concerning that of the acids and alkalies, xxvi. 168; neutral sulphate of potassia obtained from the solution of the triple compound of potassium in sulphuric acid, xxxiii. 40; that of silica in glass by potassium, 126.

DEDIER, Antoine, account of his Dissertation de Morbis Veneriis, xxiv. 25; his character as a theorist, ibid.

DEERING, Dr. his opposition to variolous inoculation, xvii. 237.

——————, John, his case of the fatal effects of the accidental use of white lead, xxv. 60.

DEFORMITY, remarkable instance of the uterus, vi. 351; memoir on that of the tongue, 353, 356, 435, 522; remarkable instance of cutaneous, viii. 508; extraordinary cases of it in one family, x. 173; on the marks produced by the imagination of the mother, xv. 249; on that of the eye, xix. 313; instances of hereditary, xxi. 235, 236; on that of the Cretins, xxii. 9, 10; of the face and throat from burning, xxvi. 158.

DEFOUR, Dr. on the plica polonica, xvi. 235.

DEFOURNELLE, Dr. dies at the age of one hundred and twenty, xxiv. 431.

DEGENER, John Hartmann, his description of the dolor facici, vi. 158.

DEGIO, Mathias, inoculates himself with the plague, vi. 366.

DEGLUTITION, on the obstructions of, ix. 293; on the use of the epiglottis in, xxxi. 318; case of impeded deglutition occasioned by derangement of the œsophagus, 392; farther remarks on the use of the epiglottis in, 463.

DEGREES, remarkable circumstance in the conferring of, i. 88; regulations in America concerning, ibid.; statutes of universities on medical, vi. 306; abuses in the grant of, 338; prevalence of those abuses in Scotland, vii. 13; method of conferring them at Edinburgh, 43; necessity of putting an end to imposition in, 161; attempt to vindicate honorary or surreptitious ones, 171; farther observations on the practice of granting them in Scotland, 366; regulations at Glasgow respecting, xii. 286; xiv. 284; on the irregular methods of obtaining, xv. 260; regulations at Cambridge concerning, 295; explanation concerning the Cambridge Statute, xvi. 344; report of the University of Edinburgh on, xviii. 190; on those granted by other universities in Scotland, xix. 466; xxii. 306; no authority obtained by virtue of them, 493; xxiii. 48; a reform necessary in the practice of bestowing them, 64; on the precedence conferred by them, 136; proposed regulations for the granting them, 188; extracts from the statute concerning, xxiv. 495; regulations at Glasgow concerning, xxviii. 338; on the scandalous prostitution of them, xxix. 2; method of graduation at Oxford and Cambridge, xxx. 266; at Edinburgh, ibid.; at Glasgow, 267; at Aberdeen and St. Andrew's, ibid.; at Dublin and at foreign universities, ibid.; conferred at Glasgow, 347; defence of the statutes of the English universities in regard to, xxxii. 323.

DEIMAN, Dr. his use of the suroxygenated muriatic acid in cutaneous diseases, x. 576.

DELAFERRONNAYS, Baron, on the virtue of the lichen islandicus, x. 379.

DELAMETHERIE, J. C. on the influence of the temperature in the phenomena of respiration, xxxii. 132; his report of the progress of science in France, 222.

DELAPORTE, M. on operations for aneurism, xxviii. 248.

DELARIVE, Dr. his account of a case of chorea, i. 21; on the virtue of cuprum ammoniacum, 22.

DELAROCHE, Dr. on the effect of high degrees of heat in the animal economy, xxiii. 307; xxix. 518; his observations on radiant heat, xxx. 396; on the determination of the specific heat of the different gases, 399.

DELAUNAY, M. his method of making mercurial ointment, iii. 581.

DELILE, M. his botanical researches in Egypt, i. 400; his account of the bohan upas, or poison tree, xxiv. 174; xxvi. 147; experiments on the effects of that poison, xxxii. 343.

DELIRIUM, benefits of opium in, i. 440, 442; remarkable case of sudden, iii. 238; appearances on dissection, 239; case proceeding from religious fanaticism, iv. 106; observations on, 258; remarks on that which is common in fevers, v. 198; efficacy of the digitalis in cases of, 442, 444; on the use of cold water in cases of, vi. 108; observed in the plague, 267; remarkable instance of it in consequence of hemiplegia, 277; on the use of blisters in, 571; in autumnal fevers, vii. 323; remarkable instances of, viii. 311; observations on, 313; on the different degrees of, ix. 72; extraordinary cases of, xiv. 16; on that observed in fever, 204; a case in which it supervened pulmonary inflammation, 391; voluntary abstinence in a case of religious, xv. 510; on the various gradations of, xvii. 87; causes of, 91; occasioned by a burn, xviii. 152; arising from intoxication, 153; occasioned by disorders of the stomach and bowels, xix. 94; utility of the actual cautery in, 432; progress of the symptoms of, xxi. 498; remarkable instances of, xxii. 210, 212, 213, 215, 216; on that produced by stramonium among the Indians, xxv. 383; remarkable case of, 384; on that produced by datura stramonium, 500; remarkable histories of, xxvii. 515; description of the disease named *delirium tremens*, xxx. 17; effects of opium in it, 19; its identity with brain fever, 77; farther observations on that complaint, 333; cases of, 334, 335; xxxi. 40; produced by religious enthusiasm, xxxi. 373; observations on that case, 464; explanation of the same, xxxii. 32; observations on the symptoms of, 336.

DELIVERY, difficult case of parturition successfully treated, i. 154; case of premature, iii. 3; on that of the placenta by manual assistance, iv. 320; opinions of the ancients on the subject, 321; the aid of art necessary in, 322; cautions for it, 323; on flooding preceding, 325; causes of the retention of the placenta by spasmodic affection, 326; on spontaneous, 487; on the advantages and disadvantages attending premature, v. 40; advantage of hastening it in a distorted patient, 253; instance of the advantage of premature, 312; Dr. Sims on difficult cases in arm presentation, vii. 481; on difficult ones of shoulder presentation, and where the arms present with the head, viii. 213; successful division of symphisis pubis in a difficult case, ix. 57; case of premature, 403; observations on that case, 404, 408; directions with respect to that of the placenta, x. 433; remarks on that paper, xi. 63; defence of it, 328; propriety of hastening it in particular cases, 496; history of a singular case of, xiv. 17; observations on that case, 155; reply to the observations, 242; farther remarks on the same, 416; on the use of the crotchet in, xv. 1; case of premature, xvi. 4; unusual circumstance attending one, 53; origin of the practice of burning the placenta, 54, *note*; cases of retroversion of the womb, with observations, 385, 402; extraordinary history of, xvii. 492; on hæmorrhage after, xviii. 284; on blood-letting in difficult cases of, 337; observations on cases of, 417; case of ruptured uterus, xix. 209; necessity of speedy delivery in such cases, 214; on bleeding in difficult cases of, xxi. 179; a new instrument for extracting the fœtus, xxii. 103; on dilatation in difficult cases of, xxiii. 14; an extra-uterine case, 462; case of uterine hæmorrhage in, 493; an extraordinary twin case, xxv. 29; an unusual case of hydrocephalus internus in, 206; singular case of twins, 311; remarks on that case, 386; case of embryulcia, 387; observations on the management of twin cases, 480; on delay in such cases, 485; xxvi. 115; on the use of the forceps and vectis in difficult cases of, xxvi. 111; farther remarks on twin cases, 282; xxvii. 367; xxviii. 104; use of opium in the pains of, xxx. 303; account of a rupture of the uterus in, 316; account of an abdominal pregnancy, 318; on the use of ergot in promoting, xxxii. 94; case of sudden death succeeding, 135; bad effects of ergot, 192; history of a rupture of the uterus during, 194; case in which a mass of hydatids and a placenta were brought away, xxxiii. 346; case of arm presentation, 514.

DELORME, M. his cases of hydatids closing the glottis, xxii. 140.

DELPECH, M. his memoir on the putrescency of hospitals, xxxiii. 399.

DELPHINUM CONSOLIDA, or wild larkspur, botanical description of the, xvii. 71; blue dye extracted from it, ibid.

DELRIO, Martin, on the magical effects of the vapour of irritated toads and vipers, vi. 441.

DELUNEL, M. on the different extracts of opium, i. 398; on the distilled waters of inodorous plants, x. 95.

DEMACHY, M. his method of preserving bitter extracts, iii. 581.

DEM-EL-MONIA, a remarkable disorder prevalent in Egypt, viii. 452.

DEMENET, M. on the convulsions incidental to pregnant women, vi. 280.

DEMERARA, account of the progress of vaccination at, ix. 65.

DEMMENIE, M. on the solution of copal in oil of turpentine, xi. 93.

DENMAN, Dr. Joseph, death and character of, xxviii. 162.

———, Dr. Thomas, his account of a remarkable abscess formed after child-birth, ii. 17; on a case of dropsy of the ovarium, 20; his case of polypus uteri, iii. 1; his mode of stating cases recommended, ibid.; his case of premature delivery, 3; his observations on the cow-pox, 292; the value of his judgment, 441; on the extraction of the placenta, 463; on the vaccine security, iv. 1; letter of the Earl of Derby to, 2; his advice followed with success, v. 312; on the dropsy of the perinæum, vii. 411; on delivery in arm presentation, 482; on the delivery of the placenta, xi. 65; on the danger of cutting the frænum of the tongue, xiv. 97; observations on his paper, 257; on a case of retroversion of the womb, xvi. 391; letter to him on the case of Anne Moore, xxi. 61; his observations on the corpora lutea in the early stage of pregnancy, 404; on the management of twin cases, xxv. 482, 483; his description of a polypus vaginæ, xxvi. 40; on the application of the vectis, 112; his opinion on the improvement of midwifery contested, 116; his objection to bark in puerperal fever, 335; on the structure of cancerous parts, xxvii. 366; on the term Quickening, 443; his case of polypus protruded at the time of labour, 467; on his knowledge of medical history, 470; on arm presentation, xxxiii. 515; on the new mode of treating cancers, xxxiv. 384; his death and character, 526.

DENMARK, progress of vaccination in that country, xvii. 295.

———, Alexander, his case of gastritis, xvi. 535; reply to his observations, xvii. 158; defence of him from an illiberal attack, xviii. 58; his farther vindication, 61; his case resembling tic douloureux produced by a wound in the radial nerve, xxxi. 499; his case of abscess in the brain, xxxiii. 60.

DENNETT, Christopher, his case of vaccine inoculation, with observations, vi. 236; on the treatment of vaccination, ix. 363; cases of small-pox, 365; on the influenza, 366; his method of preserving vaccine virus, x. 41; letter to him on the superiority of his vaccinators, 159; his case of inflammation after inoculation for the cow-pox, 321; his opinions controverted, 421.

DENTARIA DIPHYLLA, or the pepper root, description of the, xxviii. 163.

DENTITION, survey of several theories respecting difficult, iv. 75; that of Dr. Hecker, 76; that of Dr. Brandis, 77; that of Dr. Wichmann, 171; symptoms of it, 173; observations on the incision of the gums, 267; application of leeches recommended in, 268; the existence of the difficulty considered as a morbid phenomenon, v. 567; on the sensibility of the gums, 568; history of the formation of the teeth, 569; inflammation occasioned by teething, ibid.; two periods of the process, 570; general observations on it, 572; practical directions on the treatment of the gums in, viii. 561; erroneous practice of giving cathartics in, xii. 108; in hereditary diseases of, xxi. 281; on the morbid effects of, xxix. 51.

DENTIFRICE, an excellent one made from the root of the water-dock, xiv. 71; utility of the krameria triandria as a tooth-brush, 128; the cinque-foil useful for loose teeth, xvi. 265.

DEPLETION, its good effects in epidemical fever, ii. 178; caution concerning the use of it, 179; strictures on the practice, iii. 226; in rigidity of the os externum, xviii. 337; beneficial in hydrocephalus internus, xix. 217; efficacious in a case of poison, 220, 222; extraordinary case of, xxiii. 175; its success in a case of convulsions, 371; carried to an uncommon extent in the fevers of camps and hospitals, xxvi. 37; observations on that practice, 108; bold extent of it in scarlatina, 447; its utility in continued fever, 473; its efficacy in diabetes, 497; on that by the lancet, xxvii. 89; improper dread of it, 90; too long postponed in hospital practice, 91; on the proper use of it, 93; fatal case of the neglect of it in visceral inflammation, 189; necessary in croup, 207; its efficacy in the cure of pneumonia, 284; on its use in rheumatism, 286; and in dysentery, 294; efficacious in diabetes, 332; case of puerperal convulsions cured by, 345; successful in hydrophobia, xxviii. 352; in typhus, 476; other cases of its efficacy in hydrophobia, xxix. 24, 35; observations on the practice in that disorder, 37; farther remarks on the same, 43; other cases of the same, 105, 491; beneficial in yellow fever, 412; on its use in hydrophobia, xxx. 19; on the introduction of it in the treatment of tropical fever, 76; ineffectual in hydrophobia, xxxi 124; bad effects of it in a case of poison by opium, xxxii. 271; on the propriety of it in intermittents, 345; unsuccessful in hydrophobia, xxxiii. 12; its efficacy in a peculiar disease of the stomach, 27; cases in which it was beneficial, 28, 31; its use in hydrophobia defended, 178; directions for its use in that case, 226; on the treatment of fever by, 332; on the necessity and utility of it in continued fever, xxxiv. 249; hydrophobia successfully treated by, 426.

DERBISHIRE, Mr. his account of a remarkable American plant called Boneset, xxvii. 428.

DERBY, account of the influenza in the town of, x. 212; meteorological table kept at, xxix. 292.

——————, Earl of, his letter on vaccination, iv. 2.

DERBYSHIRE, on the bronchocele in, xvi. 207; account of a peculiar species of lichen in, xx. 161; mineralogical productions of, xxi. 14; on the salubrity of that county, 95.

——————————, (a practitioner of,) cases of paraphymosis related by, xvi. 140; the same on amputation, xvii. 537; on a demonstration of the brain, 539; on infantile diseases, ibid.; on a case of aneurism, xviii. 48; a reply to his paper on aneurism, 156; his vindication, 309; answer to him, by Crito, 374; his rejoinder, 432; his case of croup, xix. 214; another of fungus hæmatodes, 215; reply of Crito to him, 234; his answer, 503.

DEROSNE, M. his experiments and observations on the oily matter of opium, xii. 117.

DERVISES, in Turkey, their practice of circumgyration in religious exercises, xix. 302.

DESCEMET, M. on the irritability of the sorrel thorn, ii. 80.

DESCROIZILLES, M. on the use of the pickle of violets as a chemical reagent, xxiii. 351.

DESESSARTZ, M. on the physical education of children, i. 310; on the necessity of bleeding infants with large heads, 390; his extraordinary history of somnambulism, xxvi. 483.

DESFONTAINES, Dr. account of his Flora Atlantica, i. 97, 201; his case of diseased liver, ix. 126; his case of an insect in the liver, xv. 26; his memoir on jalap, xxi. 392; additions to that memoir, ibid.; his treatise on champignons, xxiii. 68.

DESGENETTES, Dr. denies the plague to be contagious or infectious, xv. 42; his observations on the effects of cold on the brain and nerves, xxxiii. 246; his account of the state of medicine in France, 298.

DESGRANGES, M. his observations on the use of the mineral acids, i. 500; on the use of tobacco smoke in asphyxia, xxv. 226.

DESKS, directions for placing writing, xiv. 12.

DESMANTHUS NATANS, a Coromandel plant described, xxv. 550.

DESMOND, W. his translation of Fourcroy's Chemical Philosophy, xviii. 513.

DESPARANGES, M. on the good effects of acetic ether in rheumatism, vi. 273, 274.

DESSAIGNES, M. on spontaneous phosphorescence, xxiii. 387.

DESSAULT, M. his death, i. 80; on diseases of the urinary passages, 411; his instrument for lithotomy, iii. 193; his case of luxation of the fermur, x. 495.

———, Pierre, ascribes syphilis to animalculæ, for which he recommends mercury, xxiv. 25.

DETONATING FULMINATIONS, experiments for the production of, i. 195; account of the detonating compound of chlorine and azote, xxx. 424.

DEVAUZ, M. on the mode of purifying melasses, i. 505.

DEVENTER, Dr. his opinion on the obliquities of the womb, xvi. 393.

DEVEZE, M. on a new method of treating effusions under the scull, vii. 216.

DEVONSHIRE, on the salubrity of the climate of, xi. 284; observations on the colic peculiar to, xiii. 21; success of vaccination in that county, xv. 527; prevalence of scarlatina in, xvi. 552; causes of the colic of, xviii. 12; frequency of consumption in, xxii. 106; that statement contested, 239; account of exotic plants growing in the open air in, xxv. 248; mildness of the atmosphere in, 249; oranges and lemons cultivated in, 250; obstinate case of the colic of, xxvi. 42; efficacy of the nitric acid in that complaint, 45; remarkable instance of mortality in, xxviii. 23; meteorological report for, xxix. 291; on the mildness of its climate, xxx 132, 134; meteorological report in, xxxiii. 188.

DEW, remarkable noxiousness of, xix. 494; essay on its nature and appearances, xxxiii. 232; on the circumstances influencing its production, ibid.; the formation of it proportioned to the clearness of the sky, 233; on the degree of cold connected with its formation, ibid.; on the theory of it, 234; peculiar bodies that attract it, 235; effect of the sun-beams on the atmosphere, 236.

DEWAR, Mr. his account of the influenza, x. 118; his observations on diarrhœa and dysentery, 279; on the virtue of cold ablution in fever, xi. 21.

DEWEES, Dr. on the use of the warm-bath in laborious parturition, i. 288; his essay on superfœtation, xvii. 489; on the efficacy of bleeding in rigidity of the os externum, xviii. 337; observations on his case of midwifery, 417; his examination of Dr. Osborn's opinions on parturition, 450, 545.

DEWITT, Dr. on the effects of datura stramonium, i. 84; on the mineral waters of New York, 86.

DEYEUX, on the benefit of salt of tartar in puerperal fever, iii. 165; his method of filtering water, xii. 96; obtains sugar from the red beet-root, xxv. 363; on the composition of writing inks, xxvii. 224.

DIABETES, general view of the nature of the diabetes mellitus, i. 102; efficacy of the acids in that complaint, with successful cases in the Royal Artillery Hospital at Woolwich, 103; observations on the symptoms of the disease, 145; another successful case of it treated on the dietetic plan, 218; historical sketch of the disease, with a remarkable case, ii. 87; dietetic regimen recommended in the complaint, 88; fatal case, with appearances on dissection, 209; five cases of the efficacy of nitrous acid in, iv. 205, 211; on the nature and treatment of the disease, v. 56; cases of it, 57; whether the quantity of urine voided is in proportion to the sustenance taken, 59; experimental observations on the matter produced in, 61; on the occurrence of phymosis in, 66; on the loss of substance occasioned by the complaint, 67; correction of an error of the press concerning, 164; degeneracy of both kidneys in, 303; occasioned by the transition of tartarous into saccharine acid, vi. 475; uncertainty of the disorder, vii. 66; sanative virtue of lime-water in, 67; case of it, 107; general remarks on the two species of that disorder, 108; on the use of opium in, 504; chemical experiments on the urine of diabetic patients, ix. 12; farther observations on the urine in cases of diabetes mellitus, xi. 174, 338; on the changes observed in the urine, 416; the successful use of the nitrous acid in a case of diabetes mellitus, xiii. 152; account of the quantity of urine voided in, 154; morbid appearances in two cases of, xiv. 268; successful case of, xv 276; two cases, with observations on the different states of the disease, 569; advantages of animal food in, xviii. 279; effects of nitric acid in, 407; on a supposed case of, xix. 162; history of a case successfully treated by animal diet and bark, 181; not a disease of debility, xx. 547; impropriety of the division of the disease into mellitus and insipidus, 548; on the saccharine nature of the urine, ibid.; state of the blood in, ibid.; effects of depletion in, 549; carbonated ammonia efficacious in that complaint, ibid.; history of a remarkable case, 550; effects of bleeding and blistering in, 551; an uncertain case, 552; another, in which the disorder was converted into diarrhœa, ibid.; on the complexion of the blood in this disease, 553; case in which it was treated on a plan of abstinence, xxii. 110; utility of the depleting system, 111; cases in which that plan was found efficacious, 250; farther observations on its utility, 252; a fatal case, xxiv. 157; on the increased action of the kidneys in that disorder, 159; abstemious diet recommended, 160; experiments to ascertain the presence of sugar in the urine, xxv. 277; on the non-existence of sugar in the blood of diabetic patients, xxvi. 462; farther observations on the same, 467; notes on the diabetes mellitus as it occurs in Ceylon, 491; effects of excessive bleeding in, 497; benefits arising from venesection in a case of, xxvii. 332; observations on diabetes insipidus, xxx. 167; cured by preparations of iron and the warm-bath, 168; a new theory, in which the disorder is ascribed to diseased action of the extreme vessels, xxxi. 146; two cases of diabetes mellitus, 152; tables of the state of urine and drink in those cases, 153, 154, 155; appearances on dissection in a fatal case, 155; on the use of venesection in diabetes mellitus, xxxii. 128; benefit of emetics in that disorder, 446; case of the effects of animal food in, xxxiii. 519.

DIAGNOSIS, on the Brunonian method of ascertaining the cause of diseases, iv. 270; on that of fever, 354; for distinguishing the rheumatic headache from other complaints, v. 397; farther observations on the method of discovering symptoms of disease, vi. 173; on that of syphilis, xv. 22; on the imperfection of knowledge in ascertaining diseases by symptoms, xxi. 443; galvanism a valuable test in particular cases, xxii. 322; on the imperfections of it in the definition of disease, xxvii. 482; on that of cataract, xxx. 70; on that of hydrocephalus internus, 240; on that of angina pectoris, xxxi. 161; on that of tic douloureux, 183; on that of the black cataract, xxxiii. 313.

DIAMOND, experiments on the combustion of the, xxxiii. 121; carbonate obtained from it, 124; chemical difference between that and other carbonaceous substances, 128; Mr. Tennant's discovery of the nature of the, xxxiv. 209; its identity with charcoal, 210.

DIAPHORETICS, the balm of Gilead an excellent one, i. 34; improper in fevers, 287; effects of the leaves of the holyoak, ii. 467; in what respects they are serviceable in puerperal fever, x. 16; hurtful in scarlatina, xxi. 253.

DIAPHRAGM, effects of electricity on the, ii. 11; case of hernia through the tendinous orifice of that of a cat, with an engraving, v. 106; on the obliquity of it and the adjacent parts, vi. 83; on its agency in dilating the thorax, xi. 84; history of a wounded, xxvii. 333; appearances on dissection, ibid.; remarkable appearance of it in hydrothorax, xxxiv. 114.

DIARRHŒA, observations on the prevalence of it in London, i. 208, 216; removed by lenient purgatives, 237; effects of ledum palustre in, iv. 98; terminates in dysentery, 300; frequent in London in 1800, 473; description of that which attends hectica, v. 485; injudiciously checked by astringents, vi. 198; fatal in the plague, 270; cause of the autumnal, 390; on the bilious, vii. 189; benefit of alkalies and absorbents in, viii. 289; produced in two brothers by opium, 292; efficacy of alkalies in cases of, 293; method of treating it in puerperal fever, 511; on its appearance in the army of Egypt, x. 279; a useful effort of nature at certain seasons, 378; how to be treated, 379; the radix lopez an excellent remedy in the colliquative, 477; use of salep in, xi. 442; fatal case of, 461; observations on that case, 518; aperients unnecessary in, xii. 102; formularies for, 103; method of treating it in children, 104; efficacy of rhubarb and nutmeg in, 105; stale malt-liquor a cause of, 106; occasioned by cathartics after eruptive diseases, 108; efficacy of the comfrey in, 126; benefit of verbascum in, 130; on the mistura cretacea in, 219; a new remedy for, 357; its connexion with dysentery, 468; on a fatal case of, xiii. 517; the prima sylvestria said to be serviceable in, xvi. 167; on the diet proper for bilious diarrhœa, 206; the cinque-foil a remedy in, 265; benefit of the tormentilla in, 266; astringents and opiates improper in, 283; ipecacuanha recommended in cases of, 467; caused by eating fruit, xviii. 372; success of calomel and jalap in, 566; on bleeding in measles when attended with, xix. 146; Dr. Cullen on the treatment of, 147; utility of exciting artificial, 424; chamomile hurtful in, 459; an incurable one in cows produced by corn horsetail, 540; spleenwort useful in, 542; owing to substances lodged in the stomach and intestines, xx. 379; submuriate of mercury and asafœtida given in, 380; severity of it at Edinburgh, 477; conversion of diabetes into, 552; on its occurrence in scarlatina, xxi. 255; observations on its occurrence in measles, 363; case of violent, 406; fatal result from, xxii. 428; brisk cathartics successful in, 437; severe cases of, xxiii. 434; topical bleeding serviceable in, 435; anodyne glysters used in, ibid.; case attended with anasarca successfully treated, xxvi. 184; on its power in carrying off the gout, 196; impropriety of astringents in, 512; on mercury in, xxvii. 183, 370; observations on chronic diarrhœa arising from ulcerated intestines, xxxii. 374; observations on puerperal fever with, 461; occasioned by inflammation of the mucous membrane of the alimentary canal, xxxiv. 18; efficacy of the cretaceous mixtures with ipecacuanha and opium in, 351.

DIAZ, Francis, an early Spanish surgeon, his discoveries, ii. 59.

DICK, Captain, on the good effects of the arctopus echinatus, xxii. 261.

——, Mr. his account of a toad found in the body of a tree, xxxiv. 169.

DICKSON, Dr. his observations on yellow fever, xx. 473; on the nature of fever, xxii. 99; on the utility of stimulants in the treatment of ulcers, xxiii. 392; his case of apoplexy cured by bleeding, xxvi. 181; on the extensive use of purgatives in fever, xxviii. 446; his observations on pemphigus, xxxii. 397.

DICTAMNUS ALBUS, on the sensible perspiration of that plant, xxv. 520.

DICTIONARIES, account of Dr. Hooper's medical, v. 482; description of Mr. Nemnich's nosological one, 587; account of Dr. Milne's botanical one, xiii. 564; account of Dr. Nysten's dictionary of medicine, xxxiii. 303; of Dumas's on the muscles, 306.

DIEMAN, Dr. on his doctrine of mental power, vii. 461.

DIEMERBROECK, Dr. his anatomical discoveries, ii. 165; on a recurrence of small-pox, xiv. 195; on the influence of cold in nasal hæmorrhage, xxii. 327; on supposed impurities in the blood, xxiii. 28; preserved himself from the plague by smoaking tobacco, 118; on his partiality to that practice, xxv. 135.

DIET, on the attention necessary to be paid to it in diseases, i. 106; recommended as proper for hypochondriacs, 149; its effects in the cure of diabetes, 218; odorous exhalations produced by, 244; rules for preserving health by, 260; rules for the aged in regard to, 494; its effects in diabetes, ii. 88; practical observations on, 301, 393, 493; on the different kinds of, iii. 65, 153, 249; recommended to be used with the digitalis, 120, 202; its effects on the physical state of nations, 250; its effects on animal life, 283; on coffee and its substitutes, v. 371; account of a practical treatise on, vi. 178; recommended to persons weakened by mercury, viii. 11; on that proper for gouty persons, xv. 177; that of vegetables recommended in some cases of diarrhœa, xvi. 206; effects of living on cold water in cancer, 383; abstinence from fermented liquors recommended in gout, ibid.; animal food serviceable in diabetes, xviii. 279; hydrocephalus caused by improper, xix. 30; an attention to it beneficial in consumption, 47; successful case of diabetes mellitus treated by, 181; moderation in it necessary at sea, 423; the form used in diet-drinks, 542; a generous one recommended in rheumatism, xx. 250; that of milk improper in some kinds of phthisis, xxi. 292; effects of an aquatic one in cancerous complaints, 508; on the benefit and reasonableness of a vegetable, xxi. 510; advantages of that regimen in schirrhous tumours, xxii. 79; fever prevented by generous, 231; effects of a proper one in diabetes, 252; observations on the nutritive qualities of bread, 403; on the use of fermented liquors, xxiii. 332; a spare one recommended in diabetes, xxiv. 160; proper attention to it necessary to prevent disorders, 250; on that favourable to corpulence, xxv. 451; effects produced on the stomach by an abstemious, 472; reasons for abstaining from animal food, xxvi. 310; that practice contested, xxvii. 47; an abstemious diet necessary to the cure of cancer, 72; defence of the vegetable regimen, 312; Suvorof's rules for, xxix. 49.

DIGESTION, efficacy of the datisca in defective, i. 190; observations on it in different persons, 397; on that of birds of prey, ii. 79; observations on that of various animals, 467; use of opium in promoting, ibid.; means of promoting a good, 494; on diseases in the organs of, iv. 15; on that of the stomach, vi. 81; remedies in a defective state of the organs of, viii. 9; inquiry into the causes of, x. 319; observations on the power of the human stomach in, xi. 44; affected by the state of the teeth, xv. 291; on the economy of nature in the process of, xvi. 205, 207; on disorders of the organs of, 272; experiments to ascertain the operation of it in a dog, 292; on the functions of the liver in, 294; on disorders of the organs of, xviii. 21; on the economy observed by nature in the progress of, 296; effect of mustard in promoting, 368; benefit of magnesia and rhubarb in, 478; case proving the sole power of the gastric juice in, xix. 162; observations on, xx. 2; on the acid formed in the organs of, 193; on the structure and uses of the spleen in, 211; its process in converting animal and vegetable matter into nutriment, 339; on the functions of the bile in, ibid.; on the importance of the liver in, 341; on diseases resulting from disordered state of the organs of, 342; causes of diseases in, 345; on the uses of the spleen in, xxi. 7; on that of the stomach, 428, 430; on the digestive qualities of meal and farinaceous seeds, xxii. 403; on acetous fermentation produced by various substances in, ibid.; on diseases in the organs of, xxiii. 247; on that of the stomach after death, 399, 428; origin of the scrofula in the organs of, xxiv. 148; definition of its process, 239; on the action of the bile in, 242; treatment adapted to strengthen, 250; on irregularities producing disorders in, 251; ascribed to the muscular trituration of the stomach, xxx. 433; on the properties of the gastric juice in, 517; on that of the stomach after death, xxxi. 3, 7; case of head-aches proceeding from defect in the organs of, 159; on that of the stomach after death, 178; experimental observations on, xxxii. 257; experiments on that of man, xxxiii. 420; uncertainty of the cause of it, 422.

DIGITALIS PURPUREA, (fox-glove), observations on the medicinal uses of, i. 56; its efficacy in dropsy, 57; caution respecting the use of it, 58; communications on its effects in consumption, 290, 383; ii. 35, 102; suggested as likely to be efficacious in aneurism of the heart, i. 331; historical facts relative to its use in consumption, ii. 70; combined with opium in pulmonary complaints, 102; observations on its virtues, 113; account of some remarkable effects of it, 118; mode of preparing and exhibiting it, 120; observations on its operation, 123; on its use in dropsy and consumption, 175; its virtues known in the seventeenth century, ibid.; proper mode of administering it recommended, 178; how collected, 239; cases of its effects in consumption, 240, 267; cases of its inefficacy in that disorder, 314, 430; observations on its supposed failure, 417; soil favourable to, 421; vindication of its powers in phthisis, iii. 120; preparation of the tincture of, 124; its beneficial effects in hydrothorax, 127; its failure in tubercular consumption, ibid.; its mode of operation, 128; remarks on its utility in phthisis, 129; case of its complete success, 130; observations on the case, 133; effects of full doses of, 134; benefit of the tincture of, 135; on the habitudes of the plant and its virtues, 151; on the formulæ of it as a remedy in consumption, 152; beneficial effects of the tincture, 153; its effects in dropsy, 202, 206; correction of mis-statements respecting its natural history, 305; necessity of guarding against an indiscriminate use of it, 307; cases of its efficacy when moderately taken, 308; directions for the cultivation of it, 309; on the preparation and preservation of the leaves, 310; benefit of its tincture in consumption, 311; on the diversity of opinion relative to its curative powers, ibid.; causes of its occasional failure, 312; improper modes of administering it, ibid.; cases of its success in consumption, 313, 315; its efficacy in strangulated hernia, 330; beneficial in uterine affection in pregnancy, 513; on the extension of its use, 514; adopted in female complaints, ibid.; on the use of it in croup, iv. 19; successful in spasmodic affection, 47; the medicinal effects of it defended, 61; objections to drying it in the sun, 63; case of hæmoptysis relieved by, ibid.; in asthma and fever, 64; on the extravagant encomiums passed upon this remedy, 127; moderation recommended in regard to it, 128; originally introduced into practice at Sudbury, by Dr. Maclean, 130; his account of its failures, 131; the manner of preparing and administering it, 133; quantity given in a day, ibid.; its benefit in measles, 134; manner of drying it, 135; its efficacy in a case of tinea capitis, ibid.; on the places where it thrives, 139; its appearance in the natural state, 140; free exposure to the sun necessary for it, 141; particulars of the cultivation of, 235; observations of various writers on its natural history, 236; proper for a garden, 237; on the action of the remedy, 241; its influence on the kidneys, 242; botanical remarks on the plant in its natural state, 243; employed generally in Yorkshire, in cases of increased vascular action, 308; observations on its high character, 309; its effects in pneumonic inflammation, ibid.; a fair trial recommended, 310; cases of its efficacy in pulmonary complaints, 311, 317; another instance of its efficacy in croup, 420; expectations of its virtue in consumption fully realized, 521; and in dropsy, 523; the tincture preferred to the powder, ibid.; cases of its success, by Dr. Drake, 524, 536; the Rhododendron Chrysanthemum a substitute for it, 537; on the different species of it, 538; on discovering its sedative powers, 539; its effects in hydrocephalus, v. 129; employed to arrest the progress of disease, 133; on the disputes relative to its antiphthisical powers, 201; instances of its success, 203; mode of administering it, 208; its effects on different constitutions, 209; its action on the kidneys, 211; table of cases in which it was used at the Royal Hospital, 215; remarkable instance of its efficacy in purulent phthisis, 260; several cases of dropsy cured by it, 348, 406; on its uncertainty, 411; its powers in epilepsy, 437; in cases of delirium, 441, 444; in pneumonic inflammation, 445; inefficacious in consumption, 446; successful in dyspnœa, 447; unsuccessful in ischuria, ibid.; not to be relied on in consumption, vi. 90; its application in surgical cases, 133; efficacious in strangulated hernia, 134; its operation in that complaint, 135; its use in hernia invalidated, 322; cases of its success in dropsy, 418; the safety of this remedy when judiciously applied, vii. 155; its success in high inflammation, 156; its use in the discharge of serous fluids, 197; in tympanites, 228; in hydrothorax, 401; caution necessary in giving it to aged patients, 403; beneficial in venereal complaints, viii. 8; two cases of phthisis pulmonalis successfully treated with, 82; benefit of combining the cicuta with, 85; supposed efficacy of it in the immoderate action of the uterus, 216; successfully applied in hydrops pectoris, ix. 94; its success in scarlatina, 249; and in phthisis, 263; beneficial in hydrothorax, 418; instance of its efficacy in phthisis pulmonalis, x. 551; two cases in which it proved of no service in the same complaint, 554, 556; remarks on those cases, xi. 59; form of preparing an excellent tincture of, 93; on its supposed effects in consumption, 149; successfully employed in a case of incarcerated hernia, 191; three cases of pulmonary consumption cured by, 286; beneficial in pneumonia, xii. 113; efficacious in dropsy, 116; successfully employed in a case of phthisis pulmonalis, xiii. 323; its virtue in hydrocephalus internus, xiv. 238; recommended in consumption, xv. 32, 461; inefficacious in chronic croup, 509; on the neglect of it as a medicine, xvi. 229; benefit of the tincture of, 230; cultivation of the plant, 231; successfully employed in consumption, ibid.; observations on its powers, ibid.; its effect on the pulse, 232; case of consumption cured by it, 233; proper directions for its use, 234; its effects in hydrothorax, 353; why inefficacious in dropsy, 356; method of administering it in hydrothorax, 356; cases of its success, 357, 358, 360, 361; objections to it answered, 424; its effects in strangulated hernia, xvii. 56; recommended in abortion, xviii. 23; botanical description of the, 172; its virtues in scrofulous tumours, ibid.; in epilepsy and phthisis, ibid.; first employed by Dr. Withering in dropsy, ibid.; its effect on the pulse, ibid.; different effects of it according to the posture of the patient, 176; recommended in hydrophobia, 401; successfully employed in hydrocephalus internus, 481; experimental inquiry into its effects on consumption, xix. 268; its efficacy in allaying excessive action of the sanguiferous system, 420; principally used in pain of the chest, 422; observations on its powers, ibid.; case of pulmonary consumption cured by it, 511; practical remarks on its use, 515; on its laxative effects, 517; how to correct its nauseating operation, ibid.; observations on its action in particular animals, xx. 13; experiments made with it by Dr. Baildon, 14; its use vindicated, 328; its influence on the heart, 331; on its exciting properties, 494; opinions of various authors on its powers, 525; its virtues questioned, 526; its mode of operation undetermined, xxi. 11; vindication of the moderate use of it, 325; Dr. Withering's observations on it, 327; examination of different opinions on its use, 328; varieties in the mode of administering it, xxii. 92; difficulty of ascertaining its effects in consumptive cases, 93; on the decay of its credit as a remedy in phthisis, 124; on the consequences of its removing hectic fever, xxiii. 241; its mode of operation in dropsy, 335; cases of its efficacy in hydrothorax, 377, 379, 384, 385; formularies and combinations of it, 386; its success in a case of ascites, xxiv. 141; cautions in gathering and preserving it, 160; effects of taking an extraordinary quantity of it, xxvi. 155; its effects in typhus, xxvii. 461; its efficacy in hydrothorax, xxviii. 134; effect of it in nervous affections and chorea sancti viti, xxix. 415; applied by friction in anasarca, xxxi. 432; not a new remedy in dropsy, xxxii. 13; useful in hæmorrhages, ibid.; its effects in a case of subclavian aneurism, xxxiii. 380.

DIJON, proceedings of the Academy of Sciences at, ix. 371; prize essay of the academy at, xxi. 332.

DILATATION, case of artificial one of the female urethra, xxii. 155; on that of the heart, 509; necessity of it in difficult parturition, xxiii. 14; case of it in the male urethra, xxiv. 524; directions for that of gun-shot wounds, xxx. 459.

DIMNESS, remarkable case of, xiv. 15; benefit of eyebright in, xviii. 170; the same remedy recommended, xxiii. 105; practical observations on, xxv. 67; causes of it, 68; remedies to be employed, 69. See *Eye*.

DIMSDALE, Baron, on variolous contagion, v. 146; his opposition to the Suttonian method of inoculation, vii. 496; his observations on the confluent small-pox, xiv. 311; on the infectious nature of inoculated small-pox, xxvi. 117.

————, Dr. W. P. his cases of the success of cold affusion in typhus, ix. 206, 282.

DIOGENES, his death occasioned by eating raw flesh, iii. 155.

DIONÆA MUSCIPULA, on its peculiar properties in entrapping insects, xxvii. 396.

DIOSCORIDES, on the virtues of hyoscyamus as an anodyne, iii. 405; his observations on the medicinal properties of the Anthemis Pyrethrum in ague and palsy, v. 330; his character as a naturalist, xxvi. 296; on the virtues of the colchicum autumnale, xxxii. 207; his account of poisoned honey near Heraclea Pontis, 393.

DIPLOMAS, copy of that granted to Dr. Jenner, xiii. 427; no authority conferred by academical, xxii. 493; xxiii. 66; statute of Henry VIII. concerning medical, xxiv. 495; remarkable one granted by Archbishop Sheldon to Lilly the astrologer, xxviii. 129.

DIRUF, Dr. remarkable proceedings at Heidelberg on his inauguration, i. 88.

DISCHARGES, case of an early one of the liquor amnii, viii. 260; on the diseases of females attended with, xxxii. 161, 259, 336; on the transparent mucous one from the vagina, 342.

DISCOVERIES, history of that of the cow-pox, i. 1; narrative of that of Galvani, 55; observations on those of Mayow in chemistry, 220, 249, 298, 326, 417; ii. 98; iii. 335; history of those made in surgery in the 16th century, ii. 54, 155, 278, 364, 453; history of the principal ones in anatomy, 60, 160, 281, 368, 459; iii. 72, 156, 256, 556; iv. 340; that of a new acid, iii. 583; observations on that of vaccination, iv. 381; v. 505; of a new fixed alkali, iv. 466; of a new cure for venereal disease, 469; on the nature of the nervous fluid, vi. 175; of the gadolinit, a new earth in Sweden, 188; on the claims of moderns to new, 406; on those of Bacon, 409; of Galileo, 410; of Newton, ibid.; Descartes, ibid.; that of the circulation of the blood, 411; that of respiration, 412; in chemistry, 413; Dr. Priestley's, 414; on those of Galvani, 416; historical statement of the same, vii. 70, 159; for rendering the poison of serpents harmless, 478; evidence on that of Dr. Jenner, 489; viii. 17, 141, 144, 152; of a youth in a savage state, viii. 376; vindication of Mr. Henry's claim to that of aromatic vinegar, ix. 307, 448; of a new metallic substance, xi. 479; of a chalybeate spring in Berkshire, xii. 240; a new vegetable acid in the white mulberry tree, 92; on the claim of Paré to that of the ligature in amputation, xiii. 97, 391; xiv. 40, 103; on that of the circulation of the blood, xiv. 74; on the early use of the needle and ligature, 111; of a sulphur spring at Darlington, 191; of a new metal called Nicolanium, xv. 390; of a large fossile animal, 391; of a worm-shell, ibid.; of a mammoth in Virginia, 486; of a new mineral in Cornwall, xvi. 95; of fluoric acid in the human teeth and ivory, ibid.; of a new acid, 191; account of Mr. Knight's, in vegetable physiology, xvii. 196; new ones in galvanism, 295; opposition made to that of Dr. Jenner, xviii. 24; of the tooth of a mammoth, 95; remarkable ones of ancient remains in America, 483; of the decomposition of the alkalies, xix. 93, 171, 286; xx. 95; a complete mammoth found in Siberia, xix. 287; of the pyrosoma atlanticum, a phosphoric zoophite, 479; of a composition to resist fire and water, ibid.; of the Jenite, a new mineral in the isle of Elba, xx. 97; of the muriatic ether, 125, 130; of the production of fire by the composition of air, 287; observations on those of Mr. Davy, xxi. 29; his discoveries vindicated, 129; of a sea-snake in the Orkneys, 262; of a mineral spring at Dudley, 439, 465; of membranous substances found in marble, ibid.; sketch of those of Mr. Davy, xxii. 11, 14; a new method of detecting arsenic, 87; of a new varnish from copal, 351; new tallow for candles, xxiii. 262; of a peculiar property in the abrus precatorius, 350; of new tests for arsenic, 448; xxiv. 145; of an uncommon cranium, xxiv. 87; of a new mode of producing artificial cold, 347; of a new blistering fly, 430; of a medicine for the gout, ibid.; of a medical spring at Cartmeal, in Lancashire, xxv. 84; test for detecting sulphuric acid in vinegar, 85; of the fossilized remains of a crocodile, 97; of a remarkable skull, 100; of a new species of urinary calculus, 254; of the transformation of the alkalies, 363; a permanent red colour for glass, ibid.; of a new salt from phosphoric acid and potass, 456; of the colouring matter of the acetate of potass, 518; of an uncommon tree, 546; of the properties of life in plants, xxvi. 8; of the foot of an extraordinary quadruped, 17; report on Dr. Davy's, 18; farther account of the same, xxvii. 14; that of Dr. Priestley on the connexion between the atmosphere and animal and vegetable life, 17; of an ink that resists chemical agents, 226; of the middle lobe of the prostate gland, xxviii. 6; of a new island thrown up by an eruption, 200; new varieties of alcyonia, 237; in the interior of Africa, 515; of a fossil crocodile, 516; on that of the middle lobe of the prostate, xxix. 238; on that of veins in mines, 248; account of those of Mr. Cavendish, 405; of new properties of light, 426; a new genus of plants in India, 429; new species of shells, ibid.; vegetables in minerals, 430; chromium in chlorite, 431; of the freezing of alchohol, ibid.; of fossils in the neighbourhood of Brentford, 507; an apparatus for condensing gases, ibid.; of a fossil turtle, 511; of fossil beds in France, 520; new salts of lead, 523; of a lamp for preventing explosions in mines, xxx. 82; of pure alumina, 85; of fossils at Highgate, with a resinous substance, 343; of a new detonating compound, 424; of a new comet, ibid.; of a new gas, xxxi. 255; of a fossil human skeleton, 338; of fossil alcyonia, 340; of a new bitumen, xxxii. 81; of a new remedy for the gout, 200; comparison of those of the ancients and moderns in surgery, 111, 297; of the iodine, 347; xxxiii. 37; account of those of Dr. Black, xxxiv. 41; history of that of Mr. Tennant on the nature of the diamond, 209; of a new metal, 212; on that of the proper capsula of the crystalline lens, 453.

DISEASE, odorous exhalations indicative of, i. 243; observed among the troops at the Cape of Good Hope, 453; alterations effected in the bones by, ii. 183; remarkable one among cats, iv. 373; on the state of the poor as contributing to contagious, v. 289; account of an endemic one in Norway, vi. 13; description of an uncommon one of the windpipe, 94; singular one of the great intestines, vii. 33; observations on that case, by Dr. Baillie, 104; fatal one engendered in a crowded ship, 383; singular case of an eruptive, viii. 106; circumstances which have a peculiar influence on, 109; on the endemic causes of, 221; on the nosological characteristics of, xix. 1; on the climacteric, xxxi. 163; observations on general and local, xxxiii. 459.

DISEASES, nosological table of, i. 11; on the Brunonian division of, 42; effects of abstinence in preventing, 43; effects of abstinence in acute, ibid.; history of pestilential, 79; method of preventing, in long voyages, 91; on the dietetic regimen in the cure of, 106; observations at sea, on board the Astrea, 91, 135, 236, 340, 347; on the Brunonian treatment of, 127; application of pneumatic chemistry in the cure of, 143; difficulty in the classification of, 146; on those of Jamaica, 226; on incurable and mortal, 331; on those of seamen, 407; of the army in the West Indies, 409; importance of anatomical knowledge in the treatment of, ii. 142; on those of the bones and teeth, 183, 380, 464; observations on bilious, 194; on febrile, 300; on pre-disposition to, iii. 48; on those of seamen, 94; and casualties in London for the year 1799, 111; analogous ones to be treated differently in different climates, 167; effects of the imagination in the cure of, 277; dangerous effect of cutting the hair on recovering from acute, 367; on those of the poor in London, 410; on the bestial origin of, 438; on those of women and children, 489; influence of the imagination in curing, 507; iv. 100; brief history of epidemical and pestilential, iii. 559; observations of Dr. Willan on those of London, 297; iv. 471; v. 482; observations on infantile, iv. 41; effect of the imagination in the cure of, 100; review of the ancient practice in acute,

151; on the proper doses of active remedies in chronic, 160; on the Brunonian diagnosis of, 270; on those of the eyes, 358; on the influence of the air in causing, 449; observations on a singularity in nervous, v. 196; on those of the tongue, 247; on those of the kidneys, 284; casualties for 1800, 302; on the influence of incurable, 377; on the medical use of alkalies in several, 470, 557; reports of those of London from 1796 to 1801, 482; observations on acute, 483; on chronic, 484; periodical, ibid.; effects of the mind on, 510; advice against contagious, 582; observations on those of children at Vienna, 586; on those of London, vi. 10; on the diagnosis of, 173; the effects of irregularities, 179; on those of the poor, 196; summary of those in London for 1801, vii. 112; on those of the bones, 415; account of a singular eruptive one, viii. 106; effects of the habits of the poor on, 110; on the want of a correct nosology of, 111; commentaries on the history and cure of, 179; on the endemic causes of, 221; effects of galvanism in certain, 250, 315; efficacy of alkalies in those of the alimentary canal, 293; difficulty of managing those of the bones, 300; remarkable ones, 302; cases proving the coincidence of, ix. 38; cautions in respect of feigned and concealed, 72; on those of children, 89; inflammation in chronic, 91; on those of seamen, 185, 282; observations on the epidemical ones prevalent in London, 385; description of a remarkable one at Paris, called the gripe, 419; remarkable one in the Bursæ Mucosæ, x. 69; description of those of Africa, 85; on the re-appearance of contagious ones, 177; account of some cured by the extraction of decayed teeth, 431; history of a case where the skin assumed a blue colour, 575; pathology of those of warm climates, xi. 158, 200; xiii. 481; essays on those of children, xi. 184; a synoptical table of, 379; on the spasmodic ones of the East Indies, 483; on those which resemble the venereal disease, xii. 176, 266; observations on those of the Highlanders, xiii. 28; connexion of the atmosphere with, 105; on retaining the ancient names of, 376; on those of India, 481; on a peculiar one of the lymphatics, xiv. 50; on the pneumonic ones of the poor, 83; a remarkable one at Gibraltar, 137; account of a clinical history of, 272; on those of the poor, 276; description and treatment of cutaneous, 362; epitome of infantile, 374; on the epidemics of Ireland, 574; one produced by an insect in the liver, xv. 26; on the identity of some, 48; on the origin and cure of constitutional diseases, 74; an extraordinary one among the labourers in a coal-mine, 93; xvii. 472; on the conversion of, xv. 131; on the radical diversity of the vaccine and variolous, 219; on those incidental to Indian seamen, 289; on those of the teeth, 371; cure of an obstinate hepatic one, 381; on those of the abdominal viscera, 481, 573; on those of the stomach, 576; on those which follow the loss of the spleen, xvi. 197; on local ones, 272; effects of cold on, 366; influenced by the weather, 382; on those of the stomach, 422; on those of the eyes, 562; xvii. 79; on those of the mind, xvii. 91; malignant one at New York, 97; on those of Rome, 122; application of galvanism in, 190; on those of the bladder, 386; on infantile, 539; on those of the ear, xviii. 6; ambiguity in the symptoms of, 20; observations on hereditary, 182; those so called promote population, 183; on the propagation of particular ones, 184; observations on those of infants, 311; case of one produced by taking corrosive sublimate, 347; on the Brunonian distinction of, 465; on some convulsive ones in Scotland, 579; on the nosological characteristics of, xix. 1, 4; on those of the bones, 79; on those of the joints, 84; observations on secondary ones subsequent to measles, 133; cases of the same, 134; how treated, 140; on connected or corresponding diseases, 148; account of an extraordinary one in the stomach, 157; on inflammatory, 183, 274; on a particular one incidental to children, 189; on the proximate causes of, 198, 334; on the seasons favourable to epidemical ones, 317; on an eruptive one of children, 344; success of carrot poultice in that complaint, 349; on that of sea-sickness, 395, 422, benefit of the actual cautery in some, 429; particular ones affected by temperature and climate, xx. 7; new arrangement of, 44; definitions of, 48; on remedying those consequent upon child-bearing, 73; on the insidious forms of, 93; new classification of, 102; description and treatment of cutaneous, 273, 371; effects of changes of the air in, 366; whether any are engendered in the atmosphere, 367; on the transmission of those of men to brutes, 368; on the causes of epidemics, 370; on those of the generative organs, 429; inquiry concerning local, 480; an extraordinary one in a child, 484; historic sketch of epidemics, 558; on those which resemble lues, 561; directions for inquiring into local, xxi. 15; on those of the Swedish seamen, 17; on those of the female organs of generation, 29; on those incidental to Europeans in hot climates, 71; on those of the East Indies, 74; on extraordinary cases of, 95; on those of particular professions, 98; new theory of the proximate causes of, 102; proofs of the theory, 105, 296, 449; xxii. 36, 227, 296, 458; xxiii. 116, 281, 469; whether two can exist in the same time and person, xxi. 109, 120; on one in the hair of children, 228; observations on hereditary or family ones, 229, 281; on the transmission of chronic, 232; questions of the Royal Society of Medicine at Paris on hereditary ones, 233; on an eruptive one at Sherborne, 250; remarkable instances of the prevalence of particular ones, 289; on embarrassing ones, xxii. 47; connexion of the atmosphere with pestilential, 105; new ones generated by war, 116; account of an organic one of the heart, 137; on those of the eyes, 167; observations on nervous ones, with remarkable cases, 208; the cold-bath a preventive of, 230; on that of dogs, 240; on those of aged seamen, 373; historical account of those which have borne the name of leprosy, 407; account of those among the soldiers who returned from Corunna, 428; a cutaneous one in Norway and Sweden, 432; intermittents form an order of themselves, 496; on those of the heart, 505; how conveyed by infected air, 514; memoir on a contagious one in France, xxiii. 33; food an agent in propagating, 35; on those of the urinary organs, 45; remarkable one of the testicles, 59; efficacy of opiate friction in certain, 71; on the general causes of, 121; report of those of the army in Spain, 155; a singular one in the West Indies, 166; on those communicated from one race to another, 169; report on that of Walcheren, 183; a singular one of the heart, 242; surgical observations on local ones, ibid.; history of organic ones of the heart, 265, 273; history of contagious, 281; on convulsions as a consequence of, 369, 480; history of an acute eruptive one of the penis, 441; on those which require the use of purgative waters, 510; on those of the biliary character, 512; ascribed to animalcula in the atmosphere, xxiv. 24, 25; arrangement of those of the heart, 27; effects of the vapour-bath in curing, 34; on those of the brain, 52, 89; on the natural method of curing, 67; on the danger of curing particular ones, 121, 214; on the various causes of inflammatory, 230; on those of the stomach, 237; case of one supposed to arise from poison, 373; an uncommon one of the throat, 425; a fatal one at New Orleans, 435; on the mortal one at Walcheren, xxv. 1; how treated, 4, 6; observations on those of troops in a long voyage, 13; on those diffused by the atmosphere, 145; on particular ones of the eye, 161; on those of the testicle, 175, 265, 360; a peculiar one among the South American Indians, 242; account of those of Mexico, 239; history of a fatal one in that country, 244; another in Saxony, ibid.; account of those of Madeira, 427; a remarkable one of the skin, 429; on general and topical bleeding in inflammatory ones, 546; on those of plants, xxvi. 8; remarkable one in America, 217; observations on those of Sicily, xxvii. 65; account of popular treatises on, 116; on those of the Icelanders, 1[illegible]; on those of the generative system, 139; on the treatment of those of debility, 145; their dependence on the state of the atmosphere, 264; effects of the solar and lunar influence on, 398; remarkable one in Devonshire, xxviii. 23; observed on board the Bellequeux, in a voyage from China, 269; on compressing the blood vessels in, 368; on the origin and treatment of local diseases, 375; on those attributed to mercurial action in the human system, 508; remarkable one in America, xxix. 14; new classification of, 250; peculiar one of the testes, with observations, 419; on those of the serous and synovial membranes, xxx. 73; account of one that affects persons employed in silvering mirrors, 78; on the division of them into general and local, 180; on the efficacy of mercurial action in, 446; general doctrine of chronical, 520; on those of the Laplanders, xxxi. 51; on those of the pancreas, 94; Indian method of curing, 209; observations on peculiar ones incidental to the sexes, 210; essay on those of the heart, 217; on those of Spain, 236; on those of the bladder, 241; new method of treating those of the spine, 245; on the pestilential one of Gibraltar, 258; a peculiar one of the skin at Aleppo, 282, 372; singular one among the crew of Sir Thomas Cavendish, 306; on those of the liver, 425; account of those of Tripoli, 478; report of those in Christ's Hospital, 479; on the comparative prevalence, mortality, and treatment of different ones, 501; on those of the cornea, 504; of the brain, 506; on the hereditary properties of, xxxii. 140; on those of the bladder, 155; on those of females, attended with discharges, 161, 239, 336; an extraordinary one, by which the sexes are changed among the Nosgay Tartars, 165; report at Paris for 1814, 255; organic ones of the heart, 305, 308; description of one affecting the meatus urinarius of females, 427; a remarkable hæmorrhagic one, xxxiii. 9; singular one in North America, 27; account of epidemical one in New York and New England, 92; on those of the toes and fingers, 151; severe one among horned cattle, 161, 401; on those of the rectum and anus, 236; report of natural ones at the Royal Military Asylum at Chelsea, 418; on those which affect the synovial membranes of the joints, 508; observations on Mr. Hunter's opinions respecting, xxxiv. 66; on the uncertain date of some, 125; on those of the arteries, 129; on those of veins, 149; on those resembling syphilis, 226, 265, 454; a remarkable one at Gibraltar, 301; on mercury in febrile, 323; on that of hospitals, 395, 467; report of those in Andalusia, 406, 498; observations on nervous, 427; remarkable one among the horses in Tartary, 437; on the epidemic ones of hospitals, 487; on those of the brain, 519.

Diseases, reports of, at the Westminster Hospital for 1799, i. 110, 134, 233, 335, 427; ii. 15, 111, 292, 319, 415; iii. 16, 112; for 1800, 208, 303, 408, 505; iv. 13, 107, 201, 474; v. 16, 320, 416; vi. 9, 198, 391; vii. 15.

———, reports of those in London, by Dr. Willan, for 1799, i. 112, 134, 234, 335, 428; ii. 16, 112, 318, 408, 410; iii. 15; for 1800, iii. 297.

———, in an eastern district of London, i. 111, 207, 235, 334, 426; ii. 14, 111, 208, 319, 413; iii. 109, 110, 207, 302, 407, 504; iv. 13, 103, 201, 472, 473; v. 14, 15, 197, 301, 319, 411, 509; vi. 8, 111, 197, 296, 389, 490; vii. 15, 111, 287, 403, 468, 556; viii. 14, 112, 202, 363, 396, 496; ix. 16, 191, 275, 375, 469, 570; x. 76, 180, 276, 378, 456, 566; xi. 83, 180, 478, 557; xii. 90, 112, 262, 382, 468, 572; xiii. 89, 186, 282; xv. 580; xvi. 93, 187, 283, 466; xvii. 77, 186, 292, 389, 482, 581; xviii. 93, 287, 373, 432, 572; xix. 92, 189, 285, 370, 477, 573; xx. 94, 192, 285, 381, 479, 564; xxi. 84, 261, 349, 434, 522; xxii. 86, 172, 259, 349, 437, 524; xxiii. 171, 349, 436; xxiv. 81, 171, 255, 347, 429, 516; xxv. 82, 178, 283, 369, 548; xxvi. 84, 256, 348, 511.

Diseases, in the Finsbury Dispensary, iii. 391, 409, 506; iv. 14, 105, 202, 298, 393, 490; v. 97, 194, 298, 392, 413; xiii. 375, 476, 567; xiv. 14, 183, 275, 378, 476, 569; xv. 90, 193, 291; xxv. 178.

———, reports of cases admitted under the care of the surgeon of the Finsbury Dispensary, viii. 108, 203, 300, 398, 493; ix. 13, 188, 272, 373, 467, 568, x. 74, 178, 275, 376.

———, Dr. Fothergill's reports of, xv. 386, 483, 578; xvi. 90, 185, 280, 380, 467, 571; xvii. 74, 183, 289, 387, 482, 579; xviii. 93, 182, 285, 371, 482, 569; xix. 90, 186, 283, 368, 475; xxx. 438, 527; xxxii. 88, 176, 353; xxxiii. 166, 254, 341, 431, 519; xxxiv. 87, 175, 263, 351, 528.

———, average of those of London in 1814, xxxi. 171, 257, 346, 433, 522; xxxii. 82, 170.

———, Mr. Want's reports of, xxxii. 352, 441. See *London.*

———, reports of those observed in the Liverpool Dispensary, v. 117; xv. 388.

———, in the Bath City Dispensary, vi. 195, 364, 388, 491; vii. 14, 189, 302, 553; viii. 15, 207, 364.

———, in the Bristol Dispensary, in 1801, v. 317, 318, 406, 411, 508; vi. 4, 112, 196, 364, 389, 491; vii. 14, 304.

———, at the Pneumatic Institution, Bristol Hot-wells, vii. 301.

———, in the parish workhouse of Sunderland, x. 454.

———, in the Westminster General Dispensary, xv. 386, 483, 578; xvi. 90, 185, 280, 380, 467, 571; xvii. 74, 183, 289, 387, 482, 579; xviii. 91, 187, 285, 371, 480, 569; xix. 90, 186, 283, 368, 475.

——— and casualties in London in 1805, xv. 200; in 1806, xvii. 96; 1807, xix. 96; for 1808, xxi. 87; for 1809, xxiii. 87.

———, account of those in the public dispensary at Lancaster, xvi. 188.

———, reports of those of Edinburgh, xviii. 565; xix. 182, 273, 278, 361, 470, 569; xx. 89, 190, 283, 378, 477, 561; xxi. 82, 168, 255, 332, 431, 517; xxii. 84, 169, 257, 346, 433, 522; xxiii. 81, 170, 257, 347, 433, 521; xxiv. 83, 172, 252.

———, report of cases in the general hospital at Nottingham, xxii. 244; xxiii. 154; xxiv. 157.

DISLOCATION, case of that of the jaw, iv. 504; remarkable one of the shoulder, v. 521; on the dangerous consequences of mistakes in supposed cases of, ix. 202, 203; remarks on, x. 89; on the treatment of it, with a case, 466; remarkable instance of the luxation of the femur, 494; benefit of sal ammoniac and vinegar in cases of, xiii. 508; how to treat that of the shoulder, xix. 290; on that of the limbs, and a new method for their reduction, xxiv. 468; instance of that of the shoulder, 469; opinion of Dr. Hunter on forcible extension in, 470; case of dislocation of the atlas, xxx. 341; of the jaw, xxxi. 187; of the spine, 271; observations on that of the jaw, 356; on that of the femur, xxxii. 473; on compound, 474; case of injury of the hip confounded with, xxxiii. 469; two cases of that of the thumb, xxxiv. 64.

DISPAU, M. P. his report of experiments on the gaseous oxide of azote, xvii. 51.

DISPENSARIES, proposals for a new one for the eye, xii. 576; proposal of one at Halesworth, in Suffolk, xv. 549; advantages resulting to medical students and practitioners in attending them, xix. 188; plan for their improvement, xxi. 86, 166; account of one for the application of heat, xxiv. 359.

DISPENSATORIES, account of the Prussian one published at Berlin, iii. 93; observations on that of the Royal College of Physicians in London, xii. 57; corrections in that of London, 219; advice with regard to alterations in, 357; remarks on modern ones, xiii. 217; observations on that of Edinburgh, 226; account of the American one, xvi. 570; remarks on that of Dublin, xviii. 541; alterations in that of London, xx. 137; short historical notices of old ones, xxii. 265; examination of those of London and Dublin, xxv. 431; critical account of Thompson's Dispensatory, xxvi. 321, 394; remarks on that of London, xxxii. 10. See *Pharmacopœias*.

DISPOSITION, on the use of that word by John Hunter, ii. 86; whether there be any disposition to disease, iii. 48; observations on the medical use of that term as applied to constitutional diseases, viii. 415; on that of different organs in the human body, xvi. 518; account of a remarkable one to bleeding in members of the same family, xx. 69; another of a similar character, 72.

DISSECTIONS, of an uncommon subject, i. 332; in cases of puerperal fever, 382, 387; of a person who died by phosphorus, 412; in a remarkable case of hydrocephalus internus, ii. 133; of a fatal case of cholera morbus, 155; in a case of diabetes, 211; in a case of hydrocephalus, 253; observations on the appearances in that disease, 329; in a case of uncommon tumour of the abdomen, 362; in concussion of the brain, 407; of a subject where the pericardium adhered to the heart, 480; of apes, ibid.; necessity of attention in conducting, iii. 17; remarkable ones of children, 20, 22; of a child where the brain was destroyed without any apparent cause, 70; in hydrocephalus internus, 117; in a case of sudden delirium, 239; in hydrocephalus, 325; in a case of dyspnœa, 522; remarks in the case of persons who died of angina pectoris, iv. 32, 223; in a case of hydrocephalus, 220; in gastrodynia, 229; in a case of diseased bladder, 392; in cases of dysphagia, 477, 479; in a case of induration of the vesiculæ seminales, 507; of a maniac, 509; that of a cat, v. 105; in apoplexia hydrocephalica, 127; in a case of abscess of the groin, 225; in diseases of the kidneys, 284; account of Bell's system of, 373, 579; vi. 80; directions concerning, v. 374, 376; vi. 80; appearances in a case of ischuria, v. 449; in a case of poison by opium, 491; of the abdominal muscles, 580; directions to be observed with regard to the stomach and spleen, vi. 80; on the situation of the liver, 81; on the obliquity of the diaphragm, 83; method of preserving anatomical preparations in, 274; of an uncommon tumour, 310; of dogs, 315, 466; after an unsuccessful performance of the Cæsarian operation, 349; of one who died by drinking spirits, 507; in a case of aneurism, vii. 102; of a woman who died of the dropsy in a state of pregnancy, 198; in cases of catarrhus suffocativus, 387; in a case of lumbar abscess, viii. 47; in a case of hydrocephalus internus, 102; of one who had a monstrous appetite, 285; of a female monster, 382; in a case of malformation of the heart, 499; in an extraordinary case of diseased liver, ix. 127; in remarkable disorders of the bowels, 183; in a case of diseased kidneys, 441; in a case of pregnancy, 470; of a monstrous fœtus, x. 172; in a case of tumour of the eye, xi. 199; in cases of aneurism, 307; in hydrocephalus internus, 402; in cases of severe dysentery, xii. 22, 24; in cases of aneurism of the aorta, 235; of dropsical enlargement of the abdomen, 251; in a case of sudden death from morbid affection of the brain,

392; appearances in a case of hydrophobia, xiii. 161; in an extraordinary appearance of the stomach, 164; in a case of chorea sancti viti, 404; in malformation of the heart, 120, 429; remarkable observations on the head in a case of, 490; of a female with a fungous tumour in the urinary organs, xiv. 78; in a case of tetanus, 134; observations on the state of the trachea in that case, 135; of a youth whose abdomen contained a fœtus, 158; in a case of diseased brain, 249; in two cases of diabetes mellitus, 268, 270; in a case of tinea capitis, 305; account of a peculiar structure in the membrane of the urethra, 469; of a blue girl, 471; in a case of fungous ulcer, 515; in a fatal case of parturition, xv. 3; of a case in which an insect was found in the liver, 27; in hydrocephalus internus, 29; in chorea sancti viti, 123; case of dolor facici where that of the nerve had no effect, 230; in a case of typhus fever, 241; in elephantiasis, 561; in stricture of the urethra, xvi. 108; in a case of retroversion of the womb, 386; appearances in a case of diseased spleen, 471; in cases of peritoneal inflammation, 514, 516; in a case of gastritis, 538; of a boy who died of tabes mesenterica, xvii. 76; in disease of the kidney, 182; on the appearances in cases of hydrophobia, 221, 222, 350; in a case of mollites ossium, 311; in a case of poison by opium, 422; changes found to have taken place in several organs, xviii. 36; of a malefactor, with appearances of the brain, 157, 310; in a case of hydrocephalus internus, 234; in a case of ruptured aorta, 391; another of ruptured liver, 392; in a case of pleuritis, 393; in a case of tetanus, 442; in a remarkable case of tuberculated liver, 488, 490; of a case of diseased liver, 525; in hydrocele, 533; in inflammation of the spinal marrow, 571; a moveable body found in the cavity of the vaginal coat of the testis, 577; in the measles, xix. 134, 135, 136; in the yellow fever, 139; benefits arising from the practice of, 154; outline of a proposed treatise on, ibid.; increase in the practice of, 156; case of an extraordinary disease of the stomach, 159; in a remarkable enlargement of the liver, 161; of no advantage in ascertaining the nature of scarlatina anginosa, xx. 123; in an instance of condensed lungs, 178; in cases of hydrophobia, 198, 201, 209; in diseases of the urethra, 314; of a little boy who died of the hydrophobia, 358; of professor Porson, 390; particulars of several detailed by Dr. Beddoes, with the cases, 411, 427, 540; of a dog supposed to be rabid, 469; remarkable appearances in the body of a child, 483; of an extraordinary tumour, 486; in a case of hydrophobia, 533; another of the same description, xxi. 58; case of hydrops medulla spinalis, 66; in a case of lacerated urethra, 218; in an abscess of the lungs, 266; in hydrophobia, 270; hydatids in a herniary sac, 279; remarks on the relations of, 297; in a case of hydrothorax, 445; proofs of the proximate causes of diseases from, 449, 456; of the ourang-outang, 511; in a case of death occasioned by pumping of rum, xxii. 31; in malformations of the heart, 71, 73, 74; of an organic disease of the heart, 139; in a case where hydatids completely closed the glottis, 142; in an organic injury of the intestines, 150; account of appearances in three cases of sudden death, 158; in a case of tumour of the brain, 162; in a case of hæmatemesis with mælena, 182; in tetanus, 188; in a case of fractured vertebræ, 250; in hydrophobia, 251; in two cases of carditis, 252; in hydrocephalus internus, 380, 382, 383, 386; in a case of strictured intestine, 394; in a case of poison by arsenic, 427; in dysenteric fever, 431; of horses bitten by a mad dog, xxiii. 5, 8, 9, 10, 12; in a contagious disease, 36; in inflammation of the intestines, 55; case of suppuration of the brain, 92, 192; in dysenteric fever, 162; in a case of coup de soleil, 199; in amenorrhœa and phthisis pulmonalis, 239; in an organic disease of the heart, 271; in a case of diseased uterus, 300; in abscess of the brain, 377; in an accident from gun-shot, 440; case of perforation of the intestines, 408; in a uterine case, 468; in a case of convulsions, 490; in a case of tetanus, 518; in an affection of the brain, xxiv. 58, 90; in apoplexy, 92, 96; in a case of metastasis, 180; in a case of wounded neck, 261; in a case of hæmatocele, 290; case of the man who was bitten by a rattle-snake, 399; on those of the rectum and anus, 415; in diseased bladder, 464; in a case of carditis, 478; in croup, 479; after castration, 524; appearances observed in mortal cases at Walcheren, xxv. 9, 12; in a case of obliterated iliac artery, 40; in an eruption of the gall-bladder, 185; of an uncommon fœtus, 279; of a singular case of carditis, 397; of fungus hœmatodes, 448; of two rabid dogs, 494; in a case of amaurosis and cophosis, 511; in a case of polypus vaginæ, xxvi. 40; in a case of poison by corrosive sublimate, 156; of enlargement of the heart, 157; in spotted fever, 221; of intususceptio, 250; of aneurism of the aorta, 251; in a case of ruptured spleen, 338; another of ruptured liver, 339, 340; of fractured spine, with an engraving, 372; of the medicinal leech, 412; appearances in apoplexy, xxvii. 153, 155; in a case of sudden death in pregnancy, 180; of a mad dog, 241; of rabid animals, 242, 244; in a case where the Cæsarean operation was performed, 331; in a case of wounded diaphragm, 333; in a case of torpor, where no satisfactory symptoms appeared, 418; extraordinary articles found in the stomach of a man, 454; on those of rabid animals, xxviii. 16; in cases of gutta serena, 28; in the inflammation of spinal marrow, 29; in a curious case of hernia cerebri, 59; in a wound of the heart, 60; in elephantiasis, 61; in extensive ossification of the semilunar valves of the aorta, 82; in cases of poison, 128; in intususceptio, with observations, 130; in a case of scirrhous stomach and stricture in the colon, 139, 140; in a case of dysphagia, 151; in a case of hernia of the brain, with an engraved representation, 186; in cases of apoplexy, 212, 215; appearances in the duodenum in cases of ulcers, 247; in a case of poison by arsenic, 349, 350; in hydrophobia at Calcutta, 351; another case of hydrophobia in London, 451; in a case of encysted tumour of the brain, 503; in aneurism of the aorta, xxix. 54; in diseases of the lungs, 55, 57; in an affection of the spine, 190; that of Isaac Casaubon, 246; in cases of tetanus, 273; in hydrophobia, 274; in a fatal case of extra-uterine pregnancy, 450; in hydrophobia, 489, 491; of an albuminous concretion in the cavity of the thorax, xxx. 73; in a case of subpericarditis, 143; of adhesion of the pericardium to the heart, 145; case of induration of the muscular tissue of the heart, 147; contraction of the left auriculo-ventricular aperture, 148; of osseous induration of the heart, 150; of carditis, 151; of rupture of the heart, 153; of perforation of the septum of the ventricles, 155; of ovarian dropsy, 163, 164; of cynanche laryngea, 166; in apoplexy, 194, 197; in a rupture of the uterus during labour, 316; in a case of abdominal pregnancy, 319; in a case of diseased uterus, 337; in one of cancerous stomach, ibid.; in a case of hæmatirrhœa, or petechia without fever, 340; in apoplexy, 374, 376; contracted intestinum rectum, 407; in a case of ruptured uterus, 445; on a case of ischuria, 485; further particulars of the same, 496; in a case of hæmatemesis, 513; in scirrhus of the intestines, 515; on the appearances of the stomach, xxxi. 7; in hooping-cough, 61, 65; in a case of hydrocephalus, 71; in a singular case of stricture, 72; in a case of apoplexy, 93; in diseased pancreas, 95, 97; in a case of diabetes mellitus, 155; in a case of inflammation of the kidneys, 157; in a case of angina pectoris, 161; in a case of hydrophobia, 166; in a case of death occasioned by laudanum, 195; in a disease of the heart, 218; particulars on opening the body of Mirabeau, 224; of a rabid dog, 235; in inflammation of the pericardium, 275; in inflammation of the pleura, 278; in a case of dropsy, 294; observations on that of white swelling of the joints, 314; in diseased liver, 354; of the brain, in a case of phrenitis, 358; in a case of abdominal inflammation, 372; in diseases of the liver, 426, 430; in a case of enteritis, 481; of a dog, to ascertain the influence of the nerves on the secretions of the stomach, 488; in a remarkable bilious fever, 494; of the arm, in a wound of the radial nerve, 500; in a case of diseased brain, 507, 510; in fungus hæmatodes, xxxii. 24; of a subject where a foreign body was lodged in the heart, 37; in cases of croup, 48, 54; in a case of abscess of the liver, xxxii. 120; in hydrothorax, 134; in cynanche laryngea, 151; in cases of laryngitis, 166; of the heart, 168; of ruptured uterus, 197; in organic diseases of the heart, 306, 308; of Joanna Southcott, 310; in peripneumony, 360; in abscess of the liver, 373; of ulcerated intestines, 375; in aneurism of the heart, with perforation of the walls of the auricle, 390; of a singular fœtus, 434; in a supposed case of hydrocephalus, 444; in aneurism of the heart, followed by ascites, 448; of malformations of the heart, 515; of a still-born fœtus, 521; in a case of death by tape-worms, xxxiii. 8; in diseases of the heart, 50, 52, 53; in abscess of the brain, 60; in laceration of the stomach and duodenum, 64; of a woman who carried a child in her womb fifty-two years, 66, 147; in congenital hernia of the heart, 72; of a white man whose skin became black, 74; in an epidemic fever, 99, 100; of Joanna Southcott, 137; in a case of phrenitis, 140; of the morbid state of the brain, 143; of a child born without brains, 152; of the brain in epileptics, 182; of an affection of the brain, 199; in cases of aneurism, 221, 222; in aneurism of the aorta, 270; in tetanus and hydrophobia, 317, 329; of a placenta without a fœtus, 333; in hooping-cough, 358; of ruptured uterus, 360, 362; on that of living animals, 402; of a tumour in the abdomen, 427; of obstructed aorta, 506; in chronic peritonitis, xxxiv. 16; in diarrhœa produced by inflammation of the alimentary canal, 19; of an enlarged head, 45; on diseases of the liver, 73, 76: of a fœtus found in a child, 80; in a case of inflammation of the alimentary canal, and malformation of the heart, 101; in diseased uterus, 102; in ovarian dropsy, and hydrothorax, 103; of a child born without a brain, 104; in a case of axillary aneurism, 121; on that of the brain, 311; history of a fœtus found in the abdomen of a young man, 317; in entero-mesenteric fever, 341; in a case of poison by arsenic, 442; in a case of crural hernia, 481; in disease of the brain, 520; in apoplexy, 521.

DISTANCES, on the means by which the eye accommodates itself to different, ii. 331; observations on the effect of objects in regulating motion respecting, xix. 297; on the mechanism of the eye in adjusting itself to, xxxiii. 157, 243.

DISTEMPER, on that of dogs, xv. 582; farther remarks on the same, xxii. 240; different from rabies, 242.

DISTICHIRIASIS, an imaginary disorder of the eyes described, xvi. 564.

DISTILLATION, disadvantages attending the common method of it in the making of vinegar, i. 80; on that of animal substances, 85; improvement in the process of, iii. 86; farther observations on that of vinegar, iv. 411; on that of oxygenated pomatum, 470; on that of the juice of gooseberries, xi. 414; experiments on fermentation in, 417; effects of it on health, xvi. 105; an acid obtained from that of wood, xxii. 439; evil attending that of ardent spirits, xxiv. 497; an ardent spirit obtained from milk, xxv. 256; substances found in that of wood, and their analogy to bitumen, xxviii. 235; means of producing a double one by the same heat, with an engraved representation of the apparatus, xxxiv. 114.

DISTORTIONS, case of one of the abdominal viscera, occasioned by whalebone stays, ii. 47; description of Mr. Watts' machine for curing limbs in a state of, iv. 93; claim to that invention, asserted by Mr. Sheldrake, 192; observations on those of the feet, 493; reply to Mr. Sheldrake on that subject, 498; on the cure of those of the legs and feet, with cases, v. 9; remarks on the same, 119; advantage of bringing on premature labour in a distorted patient, 253; farther observations on those of the legs and feet, 264; answer to Mr. Sheldrake on the same, 426; successful management of those of the feet, with an engraving, 456, 467; on distortion of the pelvis, xxx. 168; occasioned by predisposition, xxxiii. 160.

DIURETICS, account of a purgative one, i. 21; on the operation of the digitalis, 58, 292; the datura stramonium one of the most powerful of them, 84; farther observations on the same remedy, 278; additional notices on the diuretic properties of digitalis, ii. 119, 182; efficacy of hepatised ammonia in incontinence of urine, 288; their utility in dropsy, iv. 214; inquiry into the action of digitalis on urinary secretion, 241; ineffectual in a case of ischuria, 332; benefit of the digitalis in dropsy, v. 348; utility of blisters in retention of urine, vi. 526; experimental observations on, vii. 474; cases of their failure, x. 423; form of preparing one from the digitalis, xi. 93; efficacy of valerian, 446; use of the bryony, 447; benefit of dog's-grass, 530; goose-grass a good one, 532; general description of, 565; madder considered among the, xii. 28; and plantago, 29; the pellitory, 31; benefit of the nettle, ibid.; use of the seeds of the violet, 223, 224; black currants, 225; rupture-wort, 227; sea eryngo, 231; common master-wort, 371; fennel, 555; pimpernella, 556; parsley, 557; dwarf elder, 556; common black elder, 567; purging flax, xiv. 63; asparagus, 66; common sorrell, 71; meadow saffron, 169; climbing birthwort, 170; benefit of the arbutus, xv. 261; use of the asarum, 268; benefit of strawberries, xvi. 264; benefit of the Malvern waters, xvii. 83; use of the water germander, 468; ground ivy, 562; white horehound, 564; use of the antirrhinum, xviii. 171; the digitalis, 172; scurvy-grass, 270; horse-radish, 273; erysimum, 364, 365; common mustard, 368, 369; juniper, 370; common mallow, 459; use of tar-water, 460; common broom, xix. 74; dyer's weed, 75; St. John's wort, 164; dandelion, 167; wild lettuce, 168; burdock, 259; wormwood, 356; corn horsetail, 539; on the diuretic qualities of the digitalis, xxi. 327; diuretic properties of elaterium, xxvi. 193; description of a liniment for retention of urine, 422; on the utility of them in ulcers, xxviii. 141; benefits of the Melksham chalybeate, xxx. 302, 383; on the virtues of the pyrola umbellata in secretion of urine, xxxii. 163; xxxiii. 507; benefit of nicotiana in retention of urine, xxxii. 520.

DIVING MACHINE, experiments with a new one at Paris, xxxii. 522.

DIXON, Dr. (of Whitehaven,) his account of the influenza in that neighbourhood, x. 110; his case of hydrophobia from the bite of a cat, xxv. 61.

———, Captain, his account of the intoxicating nature of the ava root, xxix. 108.

DOBBYN, W. H. on the spontaneous origin of rabies, xiii. 478.

DOBEIMER, M. his method of making white lead, xiv. 383.

DOBSON, Dr. suggested the use of mercury in hydrocephalus, ii. 255.

DOCK, botanical description of four species of, xiv. 70, 71; efficacious in dysentery, 70; a cure for the scurvy, 71; beneficial in rheumatism, ibid.; antiscorbutic qualities of the, 72.

DOCKER, Thomas, on the beneficial effects of opiate frictions, iii. 102; farther observations on the same subject, vii. 258.

DOD, Dr. Pierce, his opposition to the practice of inoculation for the small-pox, iv. 7; his case of secondary small-pox, xvii. 237.

DODDER, (*Cusucala Europæa,*) botanical description of the, xii. 37; celebrated formerly for its cathartic qualities, ibid.

DOGBERRY-TREE, (*Cornus Sanguinea,*) botanical description of the, xii. 30.

DOG'S GRASS, (*triticum repens,*) botanical description of the, xi. 530; its diuretic qualities, ibid.; made into bread, ibid.

DOG'S MERCURY, (*Mercurialis perennis,*) botanical description of, xv. 71; used in the Hebrides to bring on salivation, ibid.; its deleterious qualities, ibid.

DOG'S ROSE, (*rosa canina,*) botanical description of, xvi. 261; conserve formed from it, 262; the leaves a substitute for tea, ibid.

DOGS, instance of the artificial impregnation of one, i. 47; remarkable disorder among, 79; effects of carbonic acid gas on, ii. 53; savage nature of, iii. 68; inoculated with small-pox, vi. 95; on the receptaculum chyli in, 221; effects of vaccine inoculation on, 314; on the distemper in, 315, 466; on the vaccine inoculation of, vii. 179; viii. 194; small-pox communicated to, 271; experiments to ascertain how long they may be kept without food, x. 22; affected by the influenza, 217; on the intestines of, xi. 375; effects of opium on, xii. 42; observed frequently to die on Mount Vesuvius, xiii. 109; recipe for the bite of mad ones, 297; experiments on them with the black vomit, 378; spontaneous origin of the rabies in, 478; recipe in Colthorpe Church for the bite of mad ones, xiv. 31; observations on it, 33; method of injecting the lymphatics in, 470; remarkable history of the bite of a mad one, 540; no mad ones at Constantinople, xv. 115; on the distemper in, 582; experiment to ascertain digestion in the stomach of one, xvi. 292; another to determine the effects of hanging on, 530; alarm of rabid ones in London, xvii. 183; go mad chiefly in hot weather, 184; signs of madness in, ibid.; on the remedies in cases of it, 185; one killed by inhaling nitrous gas, 189; effects of the bite of mad, 219; on ascertaining the symptoms of madness in, 286; two kinds of madness in, 287; benefit of the volatile alkali in the bite of mad ones, 293; remarkable instances of mad dogs, 345, 353; the abdomen of a live one opened, to ascertain the difference in the arterial and venous blood, xviii. 294; causes of rabies in, 397; hydrophobia produced by the bite of one not mad, 476; copy of an old recipe for the bite of mad dogs, xx. 10; on the structure and use of the spleen in, 212, 217; efficacy of the lichen on rabid, 271; their fondness for truffles, 355; why mad ones do not bark, 361; particulars of a mad one, 400; on a disease resembling rabies among, 468; dissection of one, 469; case of one poisoned by the ranunculus arvensis, xxi. 12; inhuman treatment of, 335; analysis of calculi taken from the bladder of one, 369; experiments with the saliva of mad ones, xxii. 64; effects of upas upon, 97; necessity of discrimination in diseases of, 112; tartrite of antimony a remedy for, 113; the spinal marrow of one divided, to ascertain whether the nerves would be paralised on either side, 164; observations on the distemper in, 240; account of horses bitten by one, xxiii. 1, 13; experiments to inoculate them with rabies, 43; precautions taken in Paris respecting, 525; on the lymphatics of one, xxv. 47; on the sense of, 137; dissection of two rabid, 449; experiments on them, to shew the influence of the brain on the action of the heart, xxvi. 58, 60; on the aorta descendens in, 83; experiments with vegetable poisons on, 134, 136; on wounds in the intestines of, xxvii. 58; dissection of a dog in a rabid state, 241; caution to be used in the diseases of, xxx. 20; dissection of a rabid one, xxxi. 235; dissection of dogs to ascertain the effects of poison, 487; similarity between the saliva of men and, xxxiii. 294. See *Hydrophobia.*

DOLÆUS, his recipe for fever, vii. 404.

DOLICHOS PRURIENS, or cowhage, its medicinal virtues, iv. 567; its efficacy as a vermifuge, xiii. 87; advice on its use, 88; farther observations on its virtue, xxviii. 413; xxix. 335.

DOLLING, W. his account of the cow-pox, viii. 154.

DOLOR FACEI, observations on that complaint as described by Fothergill, ii. 465; treated with belladonna, 466; varieties noticed in that complaint, iii. 575; remarks on the history and nature of the disease, vi. 158; writers who have treated of it, 159; observed in both sexes, 160; appearances of it, 163; on the exanthemata in that disorder, 243; cases of it, 244, 246; successful operation in, x. 420; description of the complaint, xiii. 367; remarkable case of it, xiv. 387; efficacy of aqua ammoniæ in, 388, 579; benefit of cicuta in, xviii. 136; case occasioned by diseased teeth, xxvii. 281; relieved by arsenical solution, 282; efficacy of tar externally applied in, xxxii. 219; relieved by belladonna, 433.

DOLORES POST PARTIUM, efficacy of the oil of sweet almonds in, vi. 71.

DOMEIER, Dr. on the cure of polydipsia, with a case of the complaint, iv. 304; on the appearance of the tongue as indicative of disease, 422; on the origin of the epidemical fever in Spain, xiii. 103; observations on his paper, xiv. 337; his remarks on the climate, manners, and amusements of Malta, xxv. 64.

DOMINGO, account of the endemic fever at Saint, i. 229; on the diseases prevalent at, 481; peculiar mode of treating fevers there, xix. 432; singular method of dancing among the old inhabitants of, xxv. 480; description of platina found there, 509.

DONOVAN, Edward, remarks on his museum, xviii. 434; his observations on the volcanic appearance of Cader Idris, xix. 382; his correct figure of the acarus coleoptratorum, xxi. 8; his memoir on malic acid, xxix. 173.

DOOLIE, a particular description of carriage used in India, i. 354.

DORIA, Andrew, said to have been born by the Cæsarean operation, ii. 457.

DORMICE, observations disproving the assertion of their torpidity, xix. 383.

DORR, Jonathan, his account of remarkable symptoms in an injury of the spine, xx. 111.

DORSEY, Dr. J. S. his account of a case of wounded brain, xviii. 359.

DOSES, those of medicine to be regulated by the state of excitability, i. 130; on gradually augmenting those of an active nature in obstinate diseases, iv. 160; on the advantage of giving large ones in particular cases, xviii. 55.

DOUBLE, Dr. on the treatment of cancerous ulcers, xxiii. 500.

DOUCHES, account of the establishment of them at Paris, xxx. 223.

DOUGLAS, Dr. Andrew, on ruptured gravid uterus, xiii. 238; on the muscular coat of the bladder, xxvii. 381.

————, J. C. his case of convulsions, xvii. 548; on the efficacy of bleeding, 550.

————, Dr. J. his account of a remarkable chalybeate spring near Reading, xii. 240.

————, Lieutenant, his intrepid and exemplary conduct on board the Chichester store ship, ix. 581.

DOUGLASS, Dr. William, his violent prejudices against inoculation at Boston, xxvi. 88, 215; vindication of his opinions on contagion, 117, 207; his observations on the danger of inoculation for the small-pox, 490, 492.

DOVER, (in Kent,) on the appearance of the coast at, i. 254; account of a hog buried under the cliff of, xxxiv. 171.

DOVER'S POWDER, observations on the use of, ii. 206; efficacious in consumption, vi. 95; beneficial in peripneumonia notha, ix. 387.

DOW, Dr. his case of diseased kidney, ix. 438.

DOYLE, Dr. his account of the influenza at Ross, in Ireland, x. 192.

DRAGON'S BLOOD, its virtue in staining wood, i. 201.

DRAKE, Dr. Nathan, on the virtue of digitalis in consumption, i. 290; ii. 70, 117; observations on his theory, 124; his cases of consumption, and the effects of digitalis in that disorder, 267; farther observations by him on the virtue of that medicine, 417; remarks on his opinions, iii. 124; answer to his essay on digitalis, 150; his correction of misrepresentations on the natural history of digitalis, 305; reply to him on the same, iv. 127, 141; farther statement by him of the efficacy of digitalis in consumption, 521; his form of using it, 523; cases of its efficacy in his practice, 524, 537; resolutions proposed by him at the Suffolk Benevolent Medical Society, xvi. 94; observations on his partiality to digitalis, 230, 231; his case of diseased spleen, 471; confirmation of his opinion on the virtue of digitalis, xxi. 330.

DRALET, M. his description of the inhabitants of the Pyrenees, xxxiii. 446.

DRAN, M. le, on the operation for the crural hernia, xx. 513.

DRANUNCULUS, or Guinea-worm, cases of it, with observations, xvi. 79; method of cure used by the Hindoos, 177; observations on the treatment of it, xvii. 168; opinion of the Hindoos concerning, 169; the effect of it, 170; benefit of assafœtida in, ibid.

DRAY, Thomas, his case of mortification of the bladder, iii. 456.

———, W. D. his defence of venesection, iii. 326; on the different modes of treating diseases, 327; on the uncertainty of medical practice, 328; his case of the efficacy of bleeding in ague, 329; reply to him on the benefit of venesection, 540.

DREAMS, on the causes of, iii. 283; none in sound sleep, xxi. 500; how allied to madness, 502; considered as indicative of disease, xxiv. 284.

DRENNAN, Dr. William, on the contagiousness of yellow fever, ix. 103.

DRESS, advantages of a flannel one, i. 39; advice concerning that of children, ibid.; cautions in regard to, ii. 394; necessity of attention to it as a preventive of diseases, xiv. 380; injurious consequences of wearing too much flannel, xxx. 411; observations on the necessity of attending to it, xxxi. 370.

DREW, Rev. Herman, his account of the cow-pox, viii. 153, 155; observations on his correspondence, ix. 483.

———, Dr. his cases of tumours in the pelvis, xiii. 178.

———, Walter, his cases of secondary small-pox, xiv. 536.

DRIBURG, in Germany, on the medical springs at, iv. 85; beneficial in the scurvy, ibid.

DRIESSEN, Professor, his experiments on ether, ii. 82.

DRINK, not to be given in large quantities to children, v. 294.

DROGHEDA, account of the influenza at, x. 527.

DROITWICH, account of the brine springs at, xxviii. 74; compared with those of Cheshire, 75.

DROP, reasons for substituting the word minim in the room of, xxxii. 15; disadvantage of measuring by the, 16.

DROPSY, on the efficacy of digitalis in, i. 57; caution in the use of that remedy, 58; cured by sulphur after the repulsion of the itch, 191; decoction of green broom efficacious in, 383; on that of the ovarium, ii. 20; on the use of digitalis in, 175; a particular species of, 182; use of the belladonna in, ibid.; on the effusion of water in, 261; spontaneous discharge through the navel in a case of, 330; success of blisters in, 469; on that of the brain, iii. 72, 117; effects of digitalis in dropsy, 202, 206, 309; observations on the cure of, iv. 212; pernicious effects of the debilitating treatment of it, 214; cases of the benefit of the invigorating treatment of it, 215, 218, 385; cured by sand, 570; efficacy of digitalis in, v. 129; in the kidneys, 285; two cases wherein the cure was effected by digitalis, 348; several other cases of the power of that medicine, 406; case of morbus maculosus hæmorrhagicus attended with, 489; cases of the success of digitalis in, vi. 418; fatal case of it in a pregnant woman, vii. 193; analogy between tympany and the, 234; on the use of mercury in, 401; cases of the efficacy of scarifications in, 406; observations on the cases, 410; on that of the perineum, 411; observations on that case, viii. 230; an ovarian cured by incision of the cyst, 387; on the dropsy in females, 388; case of mistake by enlarged ovaria, 444; water extracted in a case of ascites, ix. 212; successfully treated at Bamberg, 271; a young woman cured by tapping, x. 498; on displacing the uterus in dropsy of the ovarium, xi. 73; roots of the bryony serviceable in, 447; efficacy of digitalis in, xii. 115, 116; utility of the marsh trefoil in, 127; efficacy of Scotch scurvy-grass in, 129; bark of the elm beneficial in, 230; case of abdominal enlargement, 249; appearances on dissection, 251; benefit of common master-wort in, 371; roots of dwarf elder a remedy in, 560; an external application in, xiii. 288; efficacy of garlic in, xiv. 65; the colchicum autumnale a cure for, 169; advantage of tapping at the navel, 418; observations on the effects of intemperance in producing, 571; on that of the brain, xv. 122; benefit of pepper stone-crop in, 265; efficacy of the warm bath in, 502; case of encysted ascites, xvi. 79; external use of oil in, 81; why the digitalis sometimes fails as a remedy, 356; on its prevalence in London, xvii. 75; on that of the eye, 83; on tapping at the navel, 209; farther observations on that operation, 351; infusion of the leaves of antirrhinum good in cases of, xviii. 171; on the first employment of the digitalis as a remedy for, 172; the scurvy-grass of service in, 271; horse-radish useful in, 273; juniper berries recommended in, 370; extraordinary quantity of medicines taken in a case of, 406; milk-wort a remedy for, xix. 73; common broom said to be effectual in, ibid.; but questioned, 74; confirmation of its powers, ibid.; efficacy of the wild lettuce in, 166; virtue of dandelion in, 167; benefit of the burdock in, 259; on its frequency in London, 478; simple method of performing the operation of tapping, xx. 262; description of an improved canula for, 264; said to be sometimes hereditary in families, xxi. 285, 286; on the use of digitalis in, 331; insidious progress of the disease, 522; not the effect of debility when it succeeds fever, xxii. 300; attends an organic disease of the heart, xxiii. 275; efficacy of digitalis in, 276; benefit of the lytta vittata, or potatoe fly, 328; on the occasional causes of it, 332; efficacy of kali præparatum in, 334; æther and its preparations, ibid.; elaterium, 335; on the operation of digitalis in, ibid.; efficacy of the vapour bath in ascites, 501; case of ascites in which tapping was ineffectual, xxiv. 85; efficacy of digitalis in a case of ascites, 141; in the pericardium, 160; its frequency among the troops at Walcheren, xxv. 2; efficacy of gamboge and elaterium in, 7; tapping fatal in, ibid.; case of suppuration of the liver resembling ascites, 164; surprising effects of tobacco in a case of, 232, 234; the lizard a remedy in, 428; observations on a case of, xxvi. 388; the effects of a change of diet in, xxvii. 315; case where tapping was performed through the vagina, 484; effects of mercury in, xxix. 212; remarkable case of ovarian, xxx. 162; case of difficult parturition occasioned by, 163; remarkable appearances of the urine in a case of, 478; on fluids discharged from the stomach in, 487; success of elaterium in a case of general dropsy, xxxi. 144; successful case of ascites in the practice of a hospital physician, xxxii. 26; tapping for it practised by Hippocrates, 115; caustics used in it by the ancients, 303; case of aneurism of the heart terminating in, 448; extraordinary quantity of fluid extracted from a female, xxxiii. 426; fatal case of ovarian dropsy mistaken for pregnancy, xxxiv. 103.

DROPWORT, (*spirea filipendula*,) botanical description of the, xvi. 171; a substitute for flour in bread, ibid.

DROSERA, round-leaved sun-dew, botanical description of, xiv. 64; its efficacy in destroying warts and freckles, ibid.

DROWNING, objections to emetics in cases of, i. 60; method of recovering persons in a state of, ii. 497; farther instructions for the recovery of persons from, vi. 84; method of removing the water from the windpipe and its branches in cases of, 93; on ascertaining whether it is accidental or wilful, ix. 75; cause of the blood becoming dark and the countenance livid in, xvi. 531; method of restoring suspended animation in cases of, 534; advice for preventing accidents by skaiting, xix. 283; successful process in recovering persons from, xx. 235; a portable bed for persons in a state of, xxiii. 338; on the benefit of tobacco in cases of, xxv. 286; causes of the stagnation of the blood in the vessels of the lungs and brain in, xxxii. 279.

DRUGGISTS, on the practice of sending prescriptions to, vii. 370, 371; proposed regulation in the profession of, xvi. 346; their encroachments on medical practice, 456, 458; their deception in the article of angustura bark, xxii. 144; on the preparation of prescriptions by, xxiii. 24; their practice defended, 138; objections to them, 139; proposed regulation in the trade of, 189; on the increased number of retail ones, 210; evil attending their compounding of medicines, xxviii. 405; observations on, xxx. 269; evil practices of them in neglecting the regulations of the college, xxxiii. 101; complaints made by them, xxxiv. 188; clauses in the statute respecting, 345.

DRUGS, impropriety of mixing too many in one formula, i. 395; on the compositions of, iii. 93; on the exhibition of powerful, xviii. 56; on the sophistication of, xx. 20; London prices of, xxx. 437, 526; xxxi. 87, 174, 262, 348, 349, 435, 436, 523, 524; xxxii. 83, 84, 171, 172, 260, 348, 437; xxxiii. 76, 77, 342, 429, 517; xxxiv. 173, 261.

DRUMMOND, Dr. Monro, on the virtue of oil in preventing contagion, xvi. 51.

DRUNKENNESS, effects of it observed in India, i. 354; human combustion the effects of, iv. 44; fatal case of, vi. 507; tubercles of the liver common in habitual, viii. 246; observations on the evil of, x. 382; a philosophical and medical essay on, xi. 568; history of the resistance of cold in, 570; chemical effects of alcohol on the body in, xii. 86; instances of combustion in, 87; on the moral acts of persons in a state of, 89; the dropsy produced by, xvii. 75; its frequency in women, 76; case of paralysis from suddenly leaving off drinking, xviii. 154; promoted by gin-shops, 287; the cause of apoplexy, xxviii. 216; produced by the ava root, xxix. 108; on the brain fever produced by, xxx. 77; cases of the same, 338; fatal case of inflammation of the abdominal viscera caused by, xxxi. 372; observations on the treatment of that case, xxxii. 89; instance of spontaneous combustion occasioned by, 164; another case of spontaneous combustion in a confirmed drunkard, xxxiii. 425.

DRYANDER, John, anatomical professor at Marburg, account of the works of, ii. 64.

——————, Mr. the botanist, his death and character, xxiv. 441; farther particulars of, xxvi. 441.

DUBERNARD, M. on the use of volatile alkali in the bite of mad dogs, xvii. 293.

DUBLIN, mortality among horses at, xiii. 216; report of the Cow-pock Institution at, 379; annual account of vaccination at, xv. 319, 321; number of hospitals at, 357; regulations of the College of Surgeons at, 361; account of the Surgical Hospital at, xvi. 510; report of the College of Physicians at, xviii. 103; another of the College of Surgeons, 110; on the pharmacopœia of, 540; report of vaccination at, xix. 452; the test of re-inoculation adopted there, xxiv. 33; report of the Cow-pock Institution at, 518; success of vaccination in the Foundling Hospital at, 522; account of the College of Surgeons at, xxv. 89; account of the School of Physic at, ibid.; remarks on the dispensatory of the College of Physicians of, 440; account of an eruptive disease at, xxvi. 20; success of vaccination at, 35; report of the Vaccine Institution at, xxvii. 191; Kirwanian Society established at, 519; memoir of that society, xxix. 173; report of the College of Physicians there on vaccination, xxx. 114; and of that of Surgeons, 118, 120; medical lectures in the university at, 261; method of conferring medical degrees at, 267; regulations of the College of Surgeons at, 270; foundation of the physical professorship at, 279; examinations at, 280; medical practice at, 281; character of the College of Surgeons at, 282; state of the apothecaries at, 286; proposed change in the College of Surgeons, 291; proceedings of the Kirwanian Society at, xxxii. 77; account of the Lock Hospital at, 229; cases of puerperal fever in the Lying-in-Hospital at, 406; new method of operating for popliteal aneurism at, xxxiii. 158; account of the Medical Society in the university of, xxxiv. 81; address of the medical students to Dr. Macartney, 82; observations on the pharmacopœia of, 488.

DUBOIS, Rev. Mr. his account of the guinea worm, and the method of cure, xvi. 177; xvii. 169; his observations on the bite of scorpions, xvii. 170; letter from Dr. Anderson to, 171.

——————, Dr. on the virtues of cuprum ammoniacum in epilepsy, i. 23; on the efficacy of moxas in cases of aphonia, ix. 389.

DUBREE, M. his formula for the composition of unguentum nutritum, xi. 176.

DUBUC, M. his experiments on opium, xii. 38.

DU BURGA, M. his experiments on the effects of charcoal on vegetable liquors, viii. 478.

DU CERF, a noted empiric, anecdotes of, xxxii. 229.

DUCK, Nehemiah, his cases of crural hernia, which terminated in gangrene, xix. 501; remarks on his cases, xx. 63; his reply, 251.

DUCTS, on the discovery of those of the sinus pelcosi, ii. 161; discovery of the thoracic, 463; on those of the liver and gall-bladder, iii 74; on the valves of the ductus choledochus, ibid.; discovery of the lachrymal, 78; on spasms in the choledochus, viii. 240; peculiarity observed in the same, xvi. 454; on the non-conducting power of the thoracic, xxv. 361; epiphora caused by obstruction of the ductus ad nasum, xxvi. 232; case of dilated foramen ovale, with an open ductus anteriosus, xxxii. 516; observations on the same case, xxxiii. 49; calculous concretion in the salivary, xxxiv. 172.

DUDAIM, observations on the plant bearing that name in the Bible, xxvii. 349.

DUDLEY, progress of vaccination at, xiv. 433; analysis of mineral waters found near, xxi. 439; farther account of the spring, 464.

DUFRESNE, M. his discovery of calculi in the bladder of a dog, xxi. 369.

DUFRESNOY, M. on the nature and cure of ring-worm, i. 308; on the virtues of the rhus radicans in palsy and hepatic diseases, i. 308; vi. 273; farther observations on the same remedy in erysipelatous eruptions, x. 480.

DUGARD, Mr. his account of the influenza at Shrewsbury, x. 215, 217.

DUHAMEL, John, a French physician of the sixteenth century, his hostility to surgeons, ii. 159.

————, M. on the ascent of the sap in plants, xiv. 209, 211.

DUMAS, Charles Louis, his observations on pulmonary consumption, ii. 50; account of his system of the muscles, iv. 71; on the cause of hunger and thirst, x. 20; his case of variety of intermittent fever attended with hydrophobia, 541; his physiological observations on the transformations of the organs of the human body, xvi. 301, 450, 517; remarks on his observations on that subject, xviii. 36; on the effects of dividing the eighth pair of nerves, xxvii. 10; character of his works, 38; on the physiognomy in hydrocephalus, 39; on the physiognomic character of epileptics, 40; his observations on arterial blood, xxix. 149; his general doctrines of chronical diseases, xxx. 520; death and character of, xxxiii. 304; account of his writings, 305.

DUMBNESS, success of galvanism in cases of, viii. 526; efficacy of moxas in particular kinds of, ix 389; cautions to parents in supposed cases of, xiii. 451; farther instances of the efficacy of galvanism in, xxii. 321; historical account of the art of teaching the dumb to speak, xxviii. 193; benefit of electricity in a case of, xxxi 37; another case in which that remedy was ineffectual, 39; occasioned by calculous concretion on the tongue, xxxii. 169; farther account of the same, xxxiv. 172.

DUMENIL, M. his plan of anatomical nomenclature, ii. 479; on new articular cavities of the bones, ii. 482; his description of extraordinary calculi extracted from the fossa navicularis, ix. 248.

DUMFRIES, sulphur collected at, ii. 297; account of a mineral spring at, 354.

DUNCAN, Dr. Andrew, sen. his treatment of chronic catarrh, i. 21; adopts the practice of vaccination, ii. 225; on the inflammation of a vein in bleeding, 348; on the internal use of lead, xviii. 13; his case of gout in an African negro, 578; his case of jaundice occasioned by a large hydatid in the liver, xix. 556; xxvi. 363; his report of diseases at Edinburgh, xxvii. 166; on the benefits of friction in the swelled knee occasioned by scarlatina, 421; his observations on the medicinal uses of the garden lettuce, xxviii. 130, 133, 135; xxix. 17.

DUNCAN, Dr. Andrew, jun. his translation of Sprenghel's History of Medicine, i. 83; his account of malformation of the urinary organs, xiii. 180; his analysis of a bone in the eye, xiv. 471; on the general treatment of lunatics, xix. 545; his analysis of the gall bladder, xxviii. 5; his improvement of the pharmacopœia, xxxiv. 496.

————, Drs. their annals of medicine analysed, i. 413; iii. 482, 552; their observations on the influenza, x. 133, 405; their experiments on cinchona, xi. 266; on Gum Kino, 278.

————, Francis, on a peculiar affection of the bowels in the East Indies, ix. 183.

————, Thomas, his case of tetanus cured by injections of tobacco smoke, xxxiv. 64; his case of convulsions after delivery, 248.

DUNDAS, Dr. David, his account of a singular disease of the heart, xxii. 73.

————, Mr. his observations on rheumatism of the heart, xxxiv. 85.

DUNN, John, his case of the efficacy of digitalis in phthisis pulmonalis, x. 551; remarks on his account, xi. 147; on the internal use of arsenic acid in obstinate intermittents, 494; his form of administering it, 498; on cases of extraordinary abstinence, xxx. 103.

————, Sir Patrick, his medical professorship in Trinity College, Dublin, xxx. 279.

DUNNING, Richard, his case of strangulated hernia, iii. 330; letter of Dr. Remmett to, 332; his observations on the vaccine discovery, 436; on the bestial origin of diseases, 438; queries on the inoculation for the cow-pox, 440; his attestation on the value of that discovery, 441; answer to him on measles after vaccination, 549; his definition of the cow-pox, iv. 146; his observations on vaccination, 171; his description of the medal voted to Dr. Jenner, v. 155; on the success of vaccination in the West of England, 156; case of premature determination of the uterus, vii. 1; on the misrepresentations of vaccination, 2; proposal for a national establishment to promote that discovery, 3; on the extirpation of the small-pox, 4; letter from Mr. Fuge to him on that subject, 7; on the aura vaccinæ, 8; on the extinction of small-pox by vaccination, 191, 2; his observations on the influenza, x. 129; his description of remarkable insects, 137; on epidemics, 139; his meteorological table, 146; letter to him on the goat pock, 339; his account of a disease among poultry, xi. 431; his experiments to ascertain the security of vaccination, xii. 264; on cutaneous affections occurring after vaccine inoculation, xiii. 417; his reply to Dr. Walker on breaking the vaccine vesicle, 542; observations on incomplete vaccination, xiv. 54; on the state of that practice at Plymouth, 56; letter from Mr. Magin to, 57; another from Mr. Little, 58; from Mr. Smith to, 60; his account of cases of incomplete vaccination, 308; on the prevalence of the small-pox, 310; his objections to variolous inoculation, xv. 60, 61; his cases of spina bifida, 236; on the danger of rupturing the vaccine vesicle, 238; reply of Dr. Walker to, 548; his letter to Dr. Clarke on ruptured vaccine vesicles, xvi. 260; on cases of failure in vaccine inoculation, 379; the result of his observations on vaccination, 574.

DUODENUM, observations on the distension of the, xvi. 302; farther remarks on the same, xviii. 36; cases of ulcers and other morbid appearances in the, xxviii. 247; case in which it was lacerated by vomiting, xxxiii. 64.

DUPIN, M. his memoir on a contagious disease in France, xxiii. 33.

DUPORTAL, M. his memoir on certain preparations of gold in medicine, xxvii. 156.

DUPREY, M. his experiments to ascertain the principle of respiration in animals, xx. 98.

DUPUYTREN, M. his description of a female monster, viii. 382; his observations on the veins of the bones, xii. 551; on the formation of the larynx in eunuchs, 575; his remarks on vaccination, xii. 337; his account of a fœtus found in a youth, xiv. 157; his experiments on respiration, xx. 98; his observations on the organs of absorption, xxv. 46; observations on his experiments on the nerves, xxix. 148.

DURA MATER, case in which it was successfully punctured, viii. 505; case of extensive fracture of the cranium, with laceration of the, xxxi. 485; its appearance in a case of diseased brain, 507; case of fractured cranium in which it was wounded, xxxii. 525; its appearance in an abscess of the brain, xxxiii. 62.

DURANDE, M. on the virtue of vitriolic ether and turpentine in biliary calculi, i. 394, 499; efficacy of his dissolvent remedy for the stone, xxxii. 169.

DURANTI, Castor, his eulogium on tobacco, xxiv. 452; extract from his Latin poem on the virtues of that plant, xxv. 319; anecdotes of him, 320.

DURHAM, analysis of mineral waters found near, xvii. 479; case of fever in which cold affusion was used effectually in the hospital at, xxiii. 159; mineralogical observations on peculiar strata in, xxviii. 76.

DURR, Dr. observes the effects produced on the pulse by solution of muriated barytes, v. 303; his observation of sudor pedum after the cessation of the menses, ibid.; his remarks on the rheumatic head-ache, 397.

DUTCH, causes of the prolific character of the, iii. 66; on the fish diet of the, 69.

DUTCH PINK, extracted from Dyer's weed, xvi. 75.

DUTREUIL, M. on the irritability of plants, ix. 291.

DUTROCHET, M. his observations on the gestation of the viper, xxx. 518; his researches on the human fœtus, xxxiii. 396.

DUVAL, Dr. his unsuccessful experiments on the efficacy of gastric juice in fever, ii. 46.

DUVERNOY, M. on the salivary glands, xii. 548; on circular fibres in the iris, xvii. 403.

DWIGHT, Dr. Benjamin, on the origin and progress of yellow fever at Catskill in the province of New York, xiii. 304; farther observations on the same, xiv. 312.

———, Dr. Nathaniel, on the use of vegetable acids in sick head-ache, i. 286.

DYCE, Dr. William, on the extraction of teeth, with an engraving representing a new instrument for that operation, i. 13; his description of an improved scarificator, 17; his account of a popular remedy for the tooth-ache, ii. 269; remarks on the same, iii. 404; his observations on a case of polydipsia, 509; his communication of a case of hydrocephalus, iv. 218; remarks on his case of polydipsia, 304, 403.

DYER'S WEED, (*reseda luteola*,)

botanical description of the, xvi. 74; Dutch pink extracted from the, 75; its efficacy in chlorosis, xxvi. 107.

DYEING, mordicants used in giving a red colour to cotton, i. 165; improvements in that of wood, 201; a green colour obtained from the privet, xi. 370; a black from the water horehound, 440; a yellow from the sweet willow, 443; the white willow gives a cinnamon colour, 444; a green obtained from the ash, 445; saffron useful in, 448; the common reed used for dying green, 529; a purple from the common heath, 530; scabious gives both green and yellow, 532; a good red from lady's bedstraw, 533; a purple from the dogberry tree, xii. 30; a yellow from the birch, 54; black from the alder, 55; a red from the bastard gromell, 124; the comfrey used in the process, 126; a good yellow given by the buck-thorn, 222; another from hops, 229; a fine green from the leaves of convallaria, xiv. 66; a yellow from the bark and root of the barberry, 69; root of the curled dock serviceable in, 70; a purple from the whortleberry, 173; a yellow from the erica, 542; use of oak bark in dyeing, xv. 70; an excellent scarlet colour for, 263; a beautiful yellow obtained from the resida luteola, xvi. 74; another from the pear-tree, 170; a violet colour from the burnet rose, 261; a red from marsh cinquefoil, 558; a yellow from meadow rue-weed, xvii. 372; a purple from marjoram, 565; the wild wood used as a basis in, xviii. 173; a green from genista, xix. 75; a red for wool, 164; ragwort gives a good green for wool, 361; a yellow from the marigold, 457; another from the anthemis, 460; a blue from the centaurea, 538; the lycopodium used to fix colours in, 541; a scarlet and purple from the lichens, xx. 160, 161, 162; an orange from the bearded lichen, 163; a yellow from the lichen vulpius, 267; a brown from lungwort, 269; an orange from lichen caperutus, 270; utility of the pyrolignite of iron in, xxii. 439; discovery of a new scarlet for, xxiii. 350.

DYSENTERY, emetics recommended in bilious, i. 63; observations on, at the Cape of Good Hope, 236; on the virtues of the alkaline salts in, 274; observations on it in the East Indies, 348, 461; causes of it, 351; effects of nitrous acid in, ii. 179; efficacy of tartarum solubile in, 181; plan for the prevention and cure of, 283; additional observations on that subject, iii. 232; effects of acids and alkali in, 233; benefit of nitrous acid and opium in, 413; effects of ledum palustre in, iv. 97; diarrhœa terminates in, 300; washing the belly with cold water in it recommended, v. 490; use of artificial musk in, 544; one attended with unusual symptoms, vi. 490; prevalent at Bristol, 492; discharge of blood in cases of, viii. 397; prevalent in Egypt, 450; benefits of the sesamum in violent cases of, 553; common in the plague, ix. 578; observations on the appearances of that disorder among the British army in Egypt, x. 280; benefit of swathing with flannel in, 281; on the treatment of it in India, xi. 204; use of jalap in, 442; observations on that complaint in a voyage to the East Indies, 508; xii. 17; comfrey beneficial in, 126; its affinity to diarrhœa, 468; efficacy of mercurial inunction in, 482; on the utility of bleeding in, xiii. 562; efficacy of the seeds of curled dock in, xiv. 70; observations on the simple, 557; analogy between it and the rheumatism, 559; its combinations with intermittent and remittent fever, 560; on its contagiousness, 561; on the utility of bleeding in, 564; on the diseases analogous to, ibid.; emetic substances used as an injection in, xv. 85; mode of their operation, 86; formularies for them, 87; virtue of houseleek in, xvi. 76; decoction of bird's cherry recommended in, ibid.; the spiræ ulmaria beneficial in, 171; fatal in the South Sea Islands, 174; root of the tormentil useful in, 267; the upland burnet used in, xvii. 70; prize question on a remedy for, 198; on the causes of, xviii. 372; cases of it after measles, xix. 135; observations on the causes and symptoms in those cases, 137; castor oil and testaceous powders serviceable in, 141; benefit of the hypericum in, 164; chamomile useful in, 459; spleenwort serviceable in, 542; the lichen islandicus a remedy in, xx. 268; scurvy a predisposing cause of, xxi. 18; on the symptoms of it at sea, 19; appearances on dissection in cases of, 451; acetate of lead recommended in, xxii. 350; mortality among the troops at Wallhajahad by, 428; treated with purgatives and emetics, 429; benefit of calomel and opium in, 431; inefficacy of cold affusion in, ibid.; on cold affusion in, xxiii. 160; benefit of cobweb in chronic, 161; account of an epidemic one at the Cape of Good Hope, xxiv. 161; fatal cases of it at Walcheren, xxv. 3; occasioned by putrid animal effluvia, 8; benefit of rhubarb and ipecacuanha in, ibid.; appearances on dissection, 12; observations on its occurrence in a long voyage, 16; cautions adopted to prevents its spreading, 20; power of calomel in, 21; opium found necessary, 22; ipecacuanha beneficial, ibid.; castor oil most efficacious, ibid.; its prevalence at Madeira, 430; observations on it at Nottingham, xxvi. 152; on the symptomatic fever characteristic of, xxvii. 292; objections to bleeding in, 293; method of treating the disorder at sea, 294; inflammation of the villous coat from, 506; on opium in, xxviii. 34; on the mercurial plan of treating it, 35; farther observations on the same, 67; utility of ipecacuanha enema in, 93; account of a new remedy for, xxix. 16; efficacy of mercury in, 215; good effects of ipecacuanha and laudanum in, xxx. 75; on the varieties observed in the, 232; extraordinary appearances in a person who died of it, xxxii. 167; on the use of ipecacuanha and opium in, xxxiii. 146.

DYSMENORRHŒA, a term applied to the suppression of the catamenia, xxi. 31.

DYSODONTOPHYSIS, remarks on a case of, xiii. 515.

DYSON, Theophilus, on the inflammatory progress of the vaccine inoculation, vi. 219; similar observations on variolous inoculation, 220; his case of inverted uterus after parturition, xv. 385.

DYSPEPSIA, on its combination with gout, i. 148; case of scorbutus connected with it, iv. 510; origin of its worst stages, vii. 425; how to prevent it, 426; caused by the diminution of the gastric fluid, x. 320; and by decayed teeth, 431; the centaury recommended as beneficial in, xii. 221; utility of carraway seeds in, 556; remarkable instance of, xiv. 477; cases supposed to be of a bilious character, xv. 194; degree of exercise to be used in, 273; produced by decayed teeth, 291; a remedy for, 364; benefit of peppermint in, xvii. 560; good effects of carbonated magnesia in, xxiii. 478; efficacy of Cheltenham waters in, 514; on the utility of emetics in, xxiv. 251.

DYSPHAGIA, remarkable case originating in a ravenous appetite, iii. 366; cases of it, with engravings, iv. 477; success of mechanical dilatation in, viii. 35; effects of mercury in, 36; benefit of the mechanical treatment of it, 38; observations on the complaint, 39; on the causes of, ix. 293; a remarkable case, attended with inflammation of the lungs, xix. 249; relieved by gum elastic introduced into the stomach, 255; case of, xxviii. 151.

DYSPNŒA, benefit of nitrate of silver in, i. 181; case of it, with observations, iii. 520; appearances on dissection, 522; benefit of artificial musk in, v. 138; effects of digitalis in, 444; benefit of cold bathing in, xviii. 165; history of a remarkable case of, xxii. 250; frequently observed in London, 438; on the paroxysms of, 507; its frequency in old age, xxxii. 576; case successfully treated with mercury, xxxiii. 174; a severe case treated as confirmed asthma, xxxiv. 175.

DYSTOCIA, or difficult labour, synopsis of the different kinds of, xxix. 424.

DYSURIA, causes and symptoms of the, i. 349; efficacy of opium in, ii. 8; use of salep in, xi. 442.

### E.

EACHARD, M. his method of preparing the tincture of opium, iii. 582.

EAGLAND, Mr. his description of a newly invented truss for the exomphalos, with a plate, xxv. 310.

EAGLE, observations on the organs of vision in the, xxix. 483.

EAR, restoration of a lost, ii. 157; history of the anatomical discoveries of the, 160; discovery of the muscles of the, 164; cause of singing in the, 231; description of acoustic tubes to assist the hearing, with an engraving, iv. 378; vi. 75; deafness cured by puncturing the tympanum of the, iv. 570; analysis of the wax of the, vi. 475; effects of galvanism on the, vii. 243, 247, 531; on obstinate cases of deafness, viii. 254; galvanism how serviceable in diseases of the, 255, 324, 526; method of applying galvanism in disorders of the, ix. 133, 134; cases of deafness and difficulty of hearing, 142, 145; improved method of relieving deafness by galvanic influence, 290; method of taking metallic casts of the, 551; on the application of galvanism to the organs of the, x. 61, 333; objections to the common methods used in that remedy, 62; mode of taking metallic casts of it not new, 171; reply on the invention, 357; the practice described, 358; claim to the discovery, 359; ear-ache common after measles, xi. 315; the seat of herpetic affections, xii. 96; proposed establishment for diseases of the, xiii. 96; garlic useful in diseases of the, xiv. 65; announcement of a treatise on the, 283; lucid description of the anatomical structure of the, xviii. 5; on peculiar diseases of the, 6; case of deafness from worms in the, 379; anatomy and physiology of the, xix. 385; on the muscles of the middle, 387; on the attachments and situation of the tensor tympani, 388; on the uses of the muscles, 391; view of the interior of the, 394; articulations and connexions of the ossicula auditus explained, 395; original theory of phonics and musical sounds, 402; outline of an original system of the physiology of the ear, 410; on the effects of the actual cautery in diseases of the, ear, 431; on conducting operations for the, 481; account of some beautiful models of the, xx. 3; on the restoration of hearing by perforating the tympanum, xxii. 191; remarkable case of fungus hæmatodes surrounding the, xxv. 493; remedy for excoriations behind the ears, 498; on the organ of hearing in the whale, xxvii. 168; analysis of lectures on the, xxviii. 517; anatomical division of the, xxix. 81; on the phenomena of sound, ibid.; on the different organs of hearing in animals, 82; peculiarity in the cuttle fish, 83; prevalence of empiricism in treating diseases of the ear, xxxi. 257; deafness cured by puncturing the membrane of the tympanum, 346; case of deafness cured by a singular process, 379; case of deafness successfully treated by the removal of a splinter from the meatus externus, xxxiii. 226; deafness cured by injections into the tympanum through the eustachian tube, 400, 525.

EARLE, Henry, his description of a new aneurismal needle, xxv. 32; his apology to Mr. Ramsden, 141; his claim to the invention of the aneurismal needle denied, 238; his case of diseased testicle, accompanied with disease of the lungs and brain, xxx. 164; on a disease affecting the extremity of the meatus urinarius of the female, xxxii. 427; his case of retention of urine relieved by the tobacco enema, 520; on contractions after burns or extensive ulcerations, xxxiii. 65; his reply to the review of Mr. Baynton on the crooked spine, 225.

——, Sir James, his essay on the means of lessening the effects of fire, iii. 191; his opinion on burns controverted, iv. 454; his observations on hydrocele, viii. 35; his observations on fractures of the lower limbs, xviii. 389; farther account of that work, xx. 4; case of suppuration of the brain, unsuspected during life, with an engraving, xxiii. 89; description of the plate illustrative of that case, 192; on his instrument for lithotomy, xxvi. 289.

EARNEST, Mr. his case of ascites, ix. 211; his account of the influenza at Sheffield, x. 387; case of phthisis pulmonalis, 554; his case of diabetes mellitus treated successfully with nitrous acid, xiii. 152; his case of incessant singultus, xiv. 514; case of fungus ulcer, ibid.; cases of chronic syphilitic complaints, 516; his case of acute rheumatism ending in paralysis, xv. 45; remarks on the same, 47; singular case of costiveness, 100; remarks on it, 101; his observations on typhus fever, 239; case of typhus, 240; observations on his case of costiveness, 336; his case of dropsy, 501; his case of lepra, xvii. 313; his observations on the proper treatment of burns and scalds, xviii. 405; on the effects of phlegmonous inflammations, 407; his judgment in favour of the oil of turpentine in burns, 408; case of scald so treated, 410; on the propriety of opening the blisters in scalds, ibid.; remarks upon his case, 413.

EARTH, contributions to the physical history of the, ii. 492; account of a new theory of the, iii. 380; on the pressure of the atmosphere on the, xiv. 213; experimental observations on the internal temperature of the, xvi. 192; granite not the nucleus of its surface, xxiii. 525; influence of the moon upon the atmosphere of the, xxv. 85, 150; on the changes produced by the deluge in the, 98; extinct races of animals in the, xxvi. 17; influence of the atmosphere on its productions, xxvii. 17, 19; extinct animals of the, 24; observations on the strata of the, 27; improvements in the knowledge of the, 28; on the fractures and fissures in the surface of the, xxviii. 75; on the formation of metallic veins in the, 76; description of the stratified rocks of salt and coal, 157; on the cause of metallic veins of the, xxix. 247; method of determining the density of the, 407; on the medicinal effects of climates, xxx. 129; consumptive persons relieved by the effluvium of fresh, xxxiii. 160; theory of dew, 234.—See *Geology*.

EARTH-NUT, botanical description of the, xii. 361.

EARTHQUAKES, observations on the frequency of, xiii. 110; account of several remarkable ones, xxiii. 282; dreadful one at Constantinople, ibid.; severe ones in England, A.D. 1000, 283; in Italy, in 1004, ibid.; throughout Europe in 1346, ibid.; in Sicily and Naples in 1693, 285; in South America in 1796, 287; observations on their effects, 470; an island swallowed up by one near the Cape of Good Hope, 526; farther particulars of the earthquake at the Cape, xxiv. 57; new island thrown up in the Western Ocean by an earthquake, xxvii. 81; account of a dreadful one in South America, xxviii. 161; phenomenon at Barbadoes from a volcanic eruption in the island of St. Vincent, 162, 251; narrative of a volcanic one off the island of St. Michael, 201; account of the eruption in the island of St. Vincent, xxix. 426, 427.

EARTHS, on the absorption of oxygen by simple, i. 302; discovery of a calcareous earth in a crystallized state, 505; affinity between the oxygenated muriatic acid and the, iii. 91; a new one found in the emerald, 173; vases made of porous earth, 174; on the vegetation of plants in different, 175; new one found in the beryl of Siberia, 587; another in the Saxon beryl, iv. 82; analysis of a new one found in Sweden, 185; on those found in the different species of chalcedony, 374; siliceous one in antimony, v. 199; analysis of that in plants, 468; account of one found in Sweden, vi. 188; analysis of the siliceous, 373; of the argillaceous, ibid.; of the sargon, ibid.; of the glucine, ibid.; of the calcareous, 374; of the strontian, ibid.; of the barytes, ibid.; new analysis of that found in the Saxon beryl, 473; on the separation of the argillaceous from the oxyd of iron, 474; on the earthy particles in corn, 568; on those found in urinary calculi, vii. 61, 64; one eaten by the inhabitants of New Caledonia, viii. 191; when combined, produce simple ones, 474; one said to have been found in the Saxon beryl, exploded, xi. 285; chemical analysis of the one found in a Swedish fossil, xii. 69; electrical experiments on the decomposition of the, xx. 383; not simple bodies, xxi. 27; on the alkaline nature of them, 28; farther observations on their elementary properties, 129; action of the liquid gum of Botany-bay, combined with the soluble alkaline earths, xxix. 478; on the changes produced by digestion in them, xxxiii. 514.

EASINGWOULD, (Yorkshire), account of the influenza at, x. 395.

EAST BOURN, (Sussex), account of an epidemic at, x. 127.

EAST, see *India*.

EATON, Thomas, his paper on the state of vaccination at Ceylon, xxi. 126.

EAU MEDICINALE, observations on that remedy, xxiv. 350; history of the discovery, ibid.; deleterious effects of it, 351; account of D'Husson, the discoverer, 352; analysis of the medicine, ibid.; its affinity to tobacco, 353; instance of its violent effects, 364; a similar remedy announced in France, called "eau de Pradier," 430; observations on its alleged efficacy in gout, 475; case in which it proved beneficial, xxv. 114; its similarity to the rhododendron, 183; the basis of it supposed to be the digitalis lutea, 184; remarks on the accounts of its efficacy, ibid.; case of its fatal effects, 207; history of the discovery, xxvi. 23; on its operation, 24; a preparation of the nicotiana, 25; experiments to ascertain its composition, 185; supposed to be opium and white hellebore, 186; cause of its violence, 187; said to be made from the elaterium, 193; letter from Mr. Moore to Dr. Jones on its composition, 224; suspected to be formed of opium and hellebore, 225; proscribed in France, 385; that nostrum severely reprobated, 480; two cases of its violent effects, 499, 500; analysis of the, xxvii. 33; supposed to have for its basis the eupatorium cannabinum, 429; French account of that remedy, xxix. 22; discovery of a remedy similar to the, xxxii. 77, 165; observations on that discovery, 198; the preparation of colchicum defended, 201; cases of its efficacy, 312; remarks on its alleged effects in gout, with farther observations on the virtue of elaterium, 380; cases of its utility, and mode of operation, 393; cases illustrating its operation, xxxii. 493; that it acts by its sedative powers, xxxiii. 3; its effects precarious, 4; its advantages as an evacuant, xxxiv. 8.

EBEL, M. account of his geological work on the Alps, xxiii. 525.

EBN SINA, an Arabian physician, his description of the leprosy, ix. 558, 560.

ECCHYMOSIS, singular case of it on the labia pudendum, occasioned by contusion, xi. 42.

ECHINODERMES, a peculiar species of fishes described, xxx. 430.

ECKEBERG, M. his account of a new earth found in Sweden, iv. 185; his description of a new mineral, viii. 573.

ECKHOLDT, Dr. his objections to the operation of œsophagotomy, vi. 99; answers to his objections, 100.

ECLECTICS, a chemical sect, account of their age and opinions, v. 174.

ECLIPSE, account of a solar one predicted by Thales, xxv. 453.

ECONOMICAL SOCIETY, prize question proposed by that of Batavia on the purification of corrupted water, i. 296.

ECONOMY, action of iron on the animal, i. 389; established law in the animal, ii. 11; considerations on political, vi. 25; effects of the carbonous gas on the animal, viii. 189; wisdom observed in the system of animal, ix. 177; view of that of the human body, 476; effects of high degrees of heat upon the animal, xxiii. 307; essay on medical, xxxi. 321; observations on the vascular, 460; defence of an essay on medical economy, 475; importance of the nervous system in the animal economy, xxxiii. 198.

ECTROPIUM, or corrosion of the eyelids, practical observations on that disease, xxix. 312; customary treatment of it, 313; new mode of operation, 314; description of a complicated form of the disease, 315; on contracted or obliterated pupil, 316; method of treating it, 318; on artificial pupil, 320; observations on various instruments for the operation, 322; treatment to be pursued after the operation, 324; success of the operation for the complicated form of it, xxix. 432.

EDDY, J. his observations and instructions on the use of trusses, iii. 387.

EDGAR, Thomas, his case of excrescence on the scrotum, xii. 204.

EDGEWORTH, Henry, his observations on the influenza in Ireland, x. 301, 302.

EDINBURGH, opposition there to the Brunonian system, i. 124; account of the practice of physic at, iv. 260; proposal for improving the surgical practice in the hospital at, 471; account of the Medical School at, v. 523; prize question of the Medical Society at, 588; remarks on the medical education at, vi. 301; history of the University of, 302; lectures there, 303; number of students, 304; professorships, ibid.; statutes of the University, 305; public examinations, 308; on the rejection of students at, 338; that University contrasted with others in Scotland, vii. 13; farther particulars of the course of education at, 42; account of the Medical Societies there, 43; character of the inhabitants of the city of, 45; Royal College of Physicians at, 47; comparison of that University with those of England, 48; account of the work entitled The Edinburgh School of Medicine, viii. 277; private medical classes at, 479; account of the Edinburgh practice of physic, x. 180, 277; account of the influenza at, 405; private lectures at, 576; criticisms on the pharmacopœia of, xi. 299; vindicated, 536; its nomenclature dangerous, 567; report on vaccination at, xii. 11; desultory remarks on the Pharmacopœia Edinburgensis, 55; account of the Edinburgh Medical and Surgical Journal for 1804, xiii. 177; proposals for the establishment of an Anatomical Museum at, 191; farther remarks on the Pharmacopœia of, 218; on the nomenclature of the College of, 219; address of the faculty there on vaccination, 286; review of the Medical and Surgical Journal, xiv. 78, 263, 469; xv. 271; xvi. 172, 470; oath administered there to medical practitioners, xvi. 456; Dr. Jenner elected a fellow of the Royal College at, xvii. 139; observations on the review of, 141, 156; analysis of the Medical and Surgical Journal, 187, 469; report of the Royal College of Surgeons at, on vaccination, xviii. 109; contents of the eleventh number of the Medical and Surgical Journal of, 175; degrees conferred in the University of, in 1807, 190; review of the twelfth number of the Journal of, 468, 577; report of diseases at, for October 1807, 565; observations on an epidemic ophthalmia at, 578; on the chemical practice of, xix. 176; account of the thirteenth number of the Medical Journal of, 179, 270; reports of diseases at, in 1807 and 8, 182, 273, 278, 361, 470, 569; description of the city, 362; on the filthiness peculiar to, 367; observations on that University as a Medical School, 465; on the frequency of the measles at, 473; the ophthalmia common at, ibid.; quantity of ardent spirits drank there, 474; account of the Medical and Surgical Journal, No. XIV. 545; observations on the ophthalmia at, 571; report of the diseases there in 1808, xx. 89, 190, 283, 378, 477, 561; xxi. 82, 168; its advantages for bathing, xx. 92; account of the fifteenth number of the Medical Journal of, 171; review of the sixteenth number of the same, 461; analysis of the seventeenth number, xxi. 146; meeting of the Wernerian Natural History Society there, 171, 262; on the medical education of that University, 185; reports of diseases for 1809, 258, 332, 431, 517; xxii. 84, 169, 257, 346, 433, 522; xxiii. 81, 176; account of the eighteenth number of the Medical Journal, xxi. 414; the same of the nineteenth number, xxii. 244, 333; practice of the bakers of, 406; account of the twentieth number of the Journal, 427, 513; prevalence of ophthalmia at, 522; analysis of the twenty-first number of the Journal, xxiii. 155, 237; on the bigotry of the inhabitants of that city, 172; report of diseases at, for 1810, 257, 347, 433, 521; xxiv. 83, 172, 252; difference among the medical professors at, xxiii. 212; account of the twenty-second number of the Journal, 428, 516; account of the twenty-third number of the Journal, xxiv. 157; on the pharmacopœia of the College of Physicians at, 340; analysis of the twenty-fourth number of the Journal, 422; proceedings of the Royal Society at, xxv. 181, 362; analysis of the twenty-fifth number of the Journal, 357, 441; meeting of the Wernerian Natural History Society of, 453; report of the Vaccine Institution at, 460; on the rocks near, xxvi. 18; account of the twenty-sixth number of the Journal, 151; proceedings of the Royal Society there, 168; of the Wernerian Society, ibid.; of the Royal Medical Society, ibid.; of the Harveian Society, 169; account of twenty-seventh number of the Journal, 337, 490; whimsical regulation respecting the surgeons at, xxvii. 116; review of the twenty-eighth number of the Journal, 159; report on the epidemic diseases there in 1810, 166; account of the twenty-ninth number of the Journal, 323, 418; analysis of the thirtieth number, xxviii. 137, 221; prize questions of the Harveian Society at, 160; proposed experimental enquiry of the Royal Society at, 161; analysis of the thirty-first number of the Journal, 494; xxix. 63; account of the thirty-second number of the same, 412; contents of the thirty-third number, xxx. 68; reports of the Colleges of Physicians and Surgeons there on vaccination, 118; temperature of, 131; contents of the thirty-fourth number of the Journal, 245, 337; reply of the Senatus Academicus to Dr. Harrison, 279; rules of the University in regard to medical graduation, 287; on the College of Physicians there, ibid.; contents of the thirty-fifth number of the Journal, 506; reply to the Edinburgh Reviewers, by Dr. Young, xxxi. 12; prize questions of the Medical Society at, xxxii. 76; answer to the Edinburgh Reviewers on their unfair treatment of Mr. Abernethy, xxxiii. 74; account of the fortieth number of the Journal, 145; contents of the forty-first number of the same, 221, 332; regulation of the Royal Medical Society at, 244; papers read to the Royal Society there, 248; prize question of the Royal Medical Society at, 514; contents of the forty-second number of the Journal, xxxiv. 57; analysis of the forty-third number, 243; analysis of the forty-fourth number, 421, 513.

EDLIN, A. his observations on the medicinal use of yeast, v. 422; his observations on measles, viii. 28; on petechia without fever, x. 444; his account of Mr. Baker's case, xii. 378; outline of his treatise on the art of bread-making, 479; observations on his account of Mr. Baker's case, xiii. 144; his narrative of that case, 274; accused of want of liberality, 360; reply to his cases of gout, 460; on his method of making yeast, xxii. 405.

EDMONSTON, Dr. Arthur, his account of the ophthalmia in the Argyleshire Fencibles, viii. 185; his observations on the contagious nature of ophthalmia, xvii. 317; account of his treatise on the varieties and consequences of ophthalmia, 379; extracts from his publication, xviii. 12; his division of the disease into two classes, 14; his observations on elephantiasis, xxiii. 516; his plan for diminishing the prevalence and fatality of hooping-cough, xxv. 359; his observations on the natural history of the Zetland sheep, xxvii. 216.

EDMONSTONE, H. his observations on the opinion that the venereal disease existed in Otaheite, xxviii. 494; answer of Dr. Adams to the same, xxix. 41; on the impropriety of returning the hernial sac unopened into the abdomen, xxxiii. 226.

EDUCATION, observations on that of children, i. 107; impropriety of bringing children up delicately, 260; plan for improving, 304; on physical education of children, 310; account of the Swedish house of, iii. 175; proposed treatise on medical, iv. 380; on the physical education of children, v. 293; advice to parents on, 296; necessity of uniformity in, 298; observations on medical, vi. 6; remarks on the state of it at Edinburgh, 301; necessity of it for medical practice, 366; account of that of a savage youth, viii. 376; on that of the Highlanders, xiii. 31; on the improvement of medical, xiv. 75; on the errors in medical, xvii. 577; outlines of a new plan of medical, xix. 467; hints on medical education in London, xxii. 302; Dr. Harrison's memorial to government on medical, xxiv. 303; on that of a surgeon, 343; exposition of the intended act for regulating medical education and practice, xxiv. 494; its improved state in Dublin, xxv. 87; remarks on the proposed plan for improving, 109, 216, 402, 492; advice concerning the study of medicine, xxvi. 68, 71; plan for extending medical, 420; regulations for pupils at St. George's Hospital, xxviii. 311; necessity of progressive improvement in proportion to the general advance of knowledge, 409; account of Medical Schools at Paris, 461; on those of London, 464; imperfection in the education of surgeons, xxix. 118; plan of study in the Medical School at New York, 253; remarks on medical education, 275; plan proposed for its improvement, 277; want of a reform in the public hospitals, xxix. 288; inquiry after a proper course of study for medical pupils, 290; on that of an apothecary, 393; on the negligence with which the apprentices of apothecaries are treated, 445; advice to them, 447; general remarks on medical study, 468; on the duties of youth apprenticed to the medical profession, 494; advice on the study of natural history, 502; statistical account of medical education in Great Britain and Ireland, xxx. 265, 296; account of the Medical School at Pavia, xxxi. 81; account of that of Pisa, 82; abuses in the education of apothecaries, 99, 105; singular regulation in Spain concerning medical practitioners, 239; plan for improving medical instruction, 324; defence of that of the English Universities, xxxii. 322, 326; state of it in Sicily, xxxiv. 186; abstract of the Bill for regulating that of apothecaries, 345; remarks on the same, 346, 347, 432, 466; advice to students, 433; account of the Medical Schools at Paris, 478.

EDWARD VI. King of England, account of his foundation of the royal hospitals, xxvii. 306.

EDWARDS, G. F. his case of inveterate scurvy, xvi. 114; his observations on umbilical hernia, xx. 405; on the mode of cure in that case, 406; his case of labour, with apoplexy and convulsion, xxxiv. 248.

————, J. his experiments with venereal virus, xxii. 312.

————, Sydenham, his extraordinary abilities as a painter of natural history, xxvii. 82; description of a plant called after him, Edwardsia Microphylla, 525; account of his engravings of English plants, xxix. 261.

————, W. his observations on the treatment of ulcers, v. 217; his case of inflamed intestines, xxiii. 54; case of ulcerated cornea, 57; case of hydrophthalmia, 125; remarks on the same, 126.

EDWARDS, Mr. of Dorchester, on the efficacy of cerussa acetata, xix. 12.

EEL, experiment to ascertain the muscular contraction of the, xxii. 459, 460; observations and experiments on the electrical one of Surinam, xxiv. 38; observations on the migration of eels, xxvi. 516; description of a new species of, xxvii. 377; farther particulars on the same subject, xxx. 433.

EFFECTS, necessity of observing them in various diseases, i. 133; observations on the effects of medicines, vii. 80; remarks on sedative effects, 522; whether effusion of the blood on the brain be the cause or an effect of death, viii. 78.

EFFLUVIA, method of correcting deleterious ones in the air, i. 46; bad effects of marsh, 94; remittent fever caused by, 139; observations on noxious, 403; investigation of the effects of pestilential, ii. 180; injury caused by azote, 181; on the medicinal application of, 469; on rendering odorous ones visible, iii. 89; on those exhaled by women in childbed, 166; cow-pox not communicable by, 248; effects of odorous ones on the brain, 260; on the noxious effluvia of marshes, vi. 499; on the effluvia exhaled in fever, vii. 255; diseases produced by noxious, 312; tar supposed to affect the virtue of vaccine matter, ix. 451; effects of those of marshes in producing epidemics, x. 139; on the effluvia from febrile animal bodies, 467; on the generation of contagious, xi. 81; the influenza occasioned by noxious, 492; difference between the effluvia of persons in health and sickness, xiv. 387; on those of manufactories, xvi. 101; noxious effects produced by septic gas on a family, xvii. 225; swarm of bees poisoned by the effluvia of sumach, 487; observations on endemic, xviii. 86; contagious effects of the effluvia of human bodies, 473; fevers produced by noxious, xx. 369; observations on deleterious, xxi. 91; diseases occasioned by dead human bodies, xxiii. 169; effects produced by the effluvia of different substances, 447; xxiv. 60, 223, 233; how the effluvia of dead bodies may produce pestilential diseases, 422; consumptive persons relieved by the effluvia of fresh dug earth, xxxiii. 160.

EFFUSION, on that of water in dropsy, ii. 261; that of blood upon the brain the cause of apoplexy, vii. 154; whether in that case the blood is arterial or venous, 207; new method of treating that of the skull, 216; apoplexy caused by that of blood on the brain, 279; the assertion controverted, 333, 363; on that of dropsy, viii. 12; maintained to be the cause of apoplexy, 69; doubtful whether a cause or an effect, 78; on the lungs after measles, xi. 315; poisonous effects of antimony in effusion into the head, xvii. 551.

EGAN, Dr. Thomas, his inquiry into the nature of gravelly and calculous concretions, xvi. 153, 221, 305, 497.

EGG, experimental observations on the formation of the, i. 505; on the fecundating principle of the, ii. 324; liquor amnii found in the unimpregnated, iii. 160; on the fecundation of the, 259; on the musky odour of the crocodile's, vi. 345; worms produced in sheep by vermicular eggs, vii. 241; medicinal uses of the eggs of fowls, xii. 471; quantity of albumen in the white of the egg, xiv. 263; the yolk composed of fatty oil and oxide of iron, xix. 191; memoir on the physiology of the egg, xxiv. 164; the eggs of oviparous animals divided into two classes, 166; description of the several parts of the egg, 168; correction relative to the publication of the memoir on the egg, 237; experiments on eggs, to ascertain the nature of vital heat, xxv. 278; farther observations on the physiology of the egg, xxvi. 7; changes produced in the arnica montana by the eggs of insects, xxvii. 407; on the membranes involving the fœtus in the egg, xxxiii. 396.

EGREMONT, the Earl of, his letters on vaccination, xiv. 574; xvi. 36.

EGYPT, method adopted to repel the plague in, i. 34; practice of beautifying by the women of, 35; on the plants of, 37; botanical discoveries in, 400; on the medical knowledge of the ancient inhabitants of, 437; ancient mode of embalming in, ii. 385; on the leprosy in, iii. 153; on the atmosphere of, vi. 13; observations on the plague in, 247; on the endemic ophthalmia of, 357; cause of ocular inflammations in, vii. 211; effects of opium on the inhabitants of, viii. 393; commencement of the hot season in, 449; diseases prevalent there, 450; description of the ophthalmia of, 555; chalky soil of, 556; the people healthy there when the Nile overflows, ix. 112; analysis of the natron of, 474; attempt to investigate the cause of the ophthalmia of, 575; observations on the diseases in the army of, x. 279; method of extracting the stone among the Egyptians, xi. 18, *note*; medical sketches of the expedition from India to, xii. 77; on the ophthalmia of, 79; observations on the plague in, 91; proposals for annihilating that disorder, 92; effects of the eating of the coriander seeds in, 369; difference of temperature between the days and nights in, 409; observations on a peculiar worm in, 434; on the use of the nitre of, 470; atrophy of the testicles observed in, 518; the flux brought originally from there, xiv. 62; remedy for diarrhœa used in, 69; antiquity of the plague in, xv. 114; quince seeds a remedy for the ophthalmia of, xvi. 283; benefit of opium in the same, 575; observations on that complaint as it appeared in the British army, xviii. 12; farther observations on the same, 474; causes of the epidemic diseases in that country, xix. 114; on the propagation of the ophthalmia of, xx. 10; on the endemics peculiar to that country, xxi. 107; observations on the Egyptian ophthalmia as it appears in England, 164; the plague ceases there with the summer solstice, xxii. 513; description of the sarcocele of, xxv. 289; causes of it in that country, 291; case of an enormous one, 292; why peculiar to men, ibid.; extirpation the only remedy, 293; cases of the complaint, 294, 296; on the Egyptian origin of the ophthalmia, 523; effect of the sands and hot winds in, 535; instance of the true Egyptian elephantiasis, xxviii. 60; observations on the ophthalmia of, xxxii. 487; destitute of trees, xxxiii. 392; burning heat of the climate, 393; the ancient inhabitants lived in caverns, ibid.; use of the pyramids as places of retreat, 394; generation of the plague there, 395.

EILSEN, in Germany, account of the mineral waters at, xx. 133.

EIMEO, in the South Sea, on the diseases prevalent in that island, xvi. 174.

ELASTIC GUM, on the impropriety of that appellation, i. 77; analysis of the substance as it appears in plants, ibid.; how to ascertain its appearance, 268; on its solution in ether, ii. 83; process of the solution, 84; method of separating it from vegetables, iii. 372; recommended in tympanites, vii. 235; description of the plants by which it is produced, with a coloured representation, xi. 398; inquiry concerning the proper method of dissolving it, xvii. 95; account of the solution of it in vitriolic ether, 454; mode of forming a varnish from it, 455; farther observations on the method of dissolving the substance, xxvi. 264, 382; query concerning the production of it, xxvii. 380; botanical description of the siphonia cahuchu from whence it is extracted, xxviii. 52; that tree supposed to be of the genus jatropha, 53; first account of the gum, ibid.; manner of its preparation, 55; only soluble in ether, 56; references to various authorities on the subject, 107; on the benefit of the elastic gum catheter, xxix. 375; xxxi. 170; the gum recommended as a proper covering for glasses containing anatomical preparations, xxx. 33; analysis of the caoutchouc of Thibet, xxxii. 434.

ELASTICITY, observations on that of the air, i. 27; on that of æriform fluids, v. 550; account of an elastic fluid extracted from atmospheric air, vii. 117; its production in marshy situations, 120; doubts of its presence in marshes, 121; necessary for animal life, xvi. 97; a power derived or an effect only, xxviii. 418; on that of water, xxix. 20.

ELATER NOCTILUCUS, a luminous insect, described, xxv. 422.

ELATERIUM, its medicinal properties as noticed by Hippocrates, xii. 470; how likely to be useful in hydrothorax, xxiii. 335; recommended in gout, xxvi. 191; the basis of the Eau Medicinale, 193; instances of its good effects in gout, 194; its efficacy in general dropsy, xxxi. 144; suffers a diminution of its power, 145; its action on the absorbents, ibid.; combined with opium, recommended in gout, xxxii. 199; form of using it in gout, 383.

ELBA, new mineral found in the island of, xx. 97.

ELDER, *(Sambucus)*, botanical description of the dwarf, xii. 558; an emetic and cathartic, 559; beneficial in dropsy, ibid.; description and virtues of the common black elder, ibid.; recommended as a cathartic, by Sydenham, 560; useful in erysipelas, ibid.; promotes expectoration, ibid; the leaves serviceable as a cataplasm, ibid.

ELECAMPANE, botanical description of the, xix. 455; its virtues in hooping-cough, ibid.; useful in dyeing, 456.

ELECTRICITY, remarks on the practical utility of metallic, i. 53; the beneficial advantages of it in various diseases, 54; on the temperature of the electric fluid, 126; fanciful theory on a sexual difference in, 176; its effects on vegetable fermentation and putrefaction, 200; successfully employed in destroying the tape-worm, 277; on the temperature of the electric fluid, 299; on the medicinal powers of, ii. 9; has no influence on the pulse, 10; cautions in regard to its application, 11; hydrocele cured by, 12; the electric fluid a powerful stimulant, 452; its connexion with heat and light, iii. 384; successful in amenorrhœa, iv. 16; on the nature of the electric matter, 83; observations on the galvanic, 115; its efficacy in a case of blindness from suppressed catamenia, v. 68; case of white swelling cured by, 346; success of it in epilepsy, 347; progress of galvanic, vi. 209; its efficacy in gonorrhœa, 277; its use in suspended animation, 319; on animal, 416; on atmospherical, vii. 71; historical account of animal, 159; the most powerful stimulant, 267; on the identity of the electric and nervous fluids, 324; experiments on the contact of metals, 478; experiments to ascertain whether fluids are conductors of caloric, 480; relation of the action of the galvanic battery to, viii. 315; less powerful than galvanism, 316; examination of the phenomena which seem to accord with the theory of two fluids, 477; on the isolated electric pile, 478; experiment for distinguishing the two fluids, 572; on atmospheric air, 573; essay on the medical application of, ix. 286; construction of a new instrument for, 291; compared with galvanism, ibid.; its effect on plants, ibid.; case of chorea sancti viti relieved by, 468; beneficial in an enlargement of the knee, 568; in another case of white swelling, 569; comparison between it and galvanism, x. 26, 331; case of amaurosis treated by, xi. 135; two periods in the history of, 524; necessity of examining and determining its theory, 525; ineffectual in hydrophobia, 539, *note*; useful in tumours of the breast, but not in cancer, xii. 75; new opinions on, xiii. 83; account of a new electrical machine, 28; observations on animal electricity, 85; its efficacy proved in gutta serena, xiv. 481; observations on the same, 482; on its effects in the cure of cataract, xvi. 1; on some chemical effects of, xvii. 93; its influence on the mineral kingdom, 198; causes of its inefficiency in medical practice, 267; defects in the instruments of, 268; directions for the application, 269; case of Mrs. Siddons cured by, 270; the clouds formed by, 485; new theory of galvanic, xviii. 245; serviceable in paralysis, 279; instance of voluntary electricity in the human body, 362; on its supposed efficacy in hydrophobia, 401; important discoveries in the agencies of, xix. 93; palsy cured by atmospheric, 120; important chemical agencies of, 190; asthma affected by negative, 327; observations on the difference of electrical shocks from a Leyden phial, xx. 287; description of a new electrical apparatus, ibid.; experiments on the decomposition of the earths, 383; practical essays on, 474; method of conducting it for medical purposes, 475; its effects on climate, xxi. 5; its effect on the alkalies, 131; potassium best procured by, 351; all chemical compounds dependent on the electrical state of their materials, xxii. 12; on the analogy of electricity and galvanism, 314; on the medicinal application of, 319; observations on the electrical eel, xxiv. 38; account of a new instrument, called the electric column, 56; effect of lightning upon a gold chain and the skin of the neck, 437; analysis of lectures on, xxv. 280; its effects on luminous animals, 515; necessity of improving the study of medical electricity, xxvi. 109; what is the best form of an electrical machine, 110; communicable to all substances, 324; beneficial in paralysis, xxvii. 101; accidents from the wind of a ball attributed to atmospherical electricity, 326; instances of persons killed by lightning, 327; on the different action of the electric fluid, 328; observations on chemical and electrical attraction, 403; on the want of a proper machine for medical purposes, 451; accidents from the wind of a ball not owing to, xxviii. 143; observations on the cylinder machine, 266; superseded by the plate machine of Van Marum, ibid.; account of Swift's machine, 267; description of an improved insulating stool, ibid.; limited knowledge of medical electricity, 268; its identity with galvanism, ibid.; farther observations on the effects of the wind of a ball, xxix. 63; account of the discoveries of Mr. Cavendish in, 406; action of electricity on the gas of marshes, 516; not the cause of accidents from the wind of a ball, xxx. 246; new theory of electricity, 249; ammonia spontaneously produced in some cases of electro-chemical decomposition, 260; description of a grand galvanic battery, by Mr. Children, 426; instances of the effects of medical electricity in restoring speech, xxxi. 37, 39; dangerous in some cases of convulsive affection, 148; its effect in trismus, xxxii. 31; phenomena observed on Mount Breven, xxxiii. 248; similar ones observed on Mount Etna, 249; galvanism and electricity said to be different principles, 337; successful in amaurosis, 469. See *Galvanism.*

ELECTROMETER, description of that instrument, vi. 209.

ELEMENTS, tables of the principal ones of vegetables, i. 75, 157; air and water not simple ones, 167; on the coercible ones which give peculiar forms to water, 195; on the formative ones in plants, 263, 377; chemical observations on the, 269, 272; of the physiology of living bodies, v. 370; the atmosphere not an homogeneous one, vii. 113.

ELEPHANT, on the coupling and gestation of the, v. 588; musky odour of the sweat of the, vi. 343; its existence in America ascertained, xv. 486; on the American, xxv. 99.

ELEPHANTIASIS, that disease occasioned by eating fish, iii. 153; a species of the disease produced by a scrofulous complaint, iv. 58, 60; on the etymology of the term, v. 25; account of a disease in Norway similar to, vi. 13; extraordinary case in a female, ix. 289; benefit of pressure exemplified in, 545; account of a singular case, 556; description of that of Barbadoes, xv. 561; remarkable instance in a negro, with an engraved representation, xvi. 363; appearances on amputation, 364; another case of a like kind, 365; effect of nitric acid in that complaint, xvii. 189; observations on that described by the ancients, xviii. 84; an hereditary disease, xxi. 287; said to be contagious, xxii. 409; particular inquiry into the nature of the disease, xxiii. 516; case of the Lepra Arabum, xxv. 84; case analogous to the elephantiasis, 400; description of its appearance in Madeira, 427; the lizard said to be a remedy for, xxvi. 25; its frequency in Iceland, and the causes, xxvii. 120; instance of the true elephantiasis, xxviii. 60; remarkable case in St. Bartholomew's Hospital, xxxi. 514; described by Aretæus, xxxii. 142; case of measles occurring with it, 524; observations on its appearance in England, xxxiii. 74; remarks on a recent description of the disease, 107.

ELETTARIA, description of that genus of plants, xxvii. 20; remarkable leeches peculiar to them, 21.

ELETTRATI, a peculiar term for gold crystallized by electricity, vi. 475.

ELFORD, Sir William, his correspondence on the discovery of the cowpox, viii. 155, 156.

ELGIN, (the Earl of,) his encouragement of vaccination while ambassador at

Constantinople, v. 352; his correspondence with Dr. De Carro, vii. 186; transmits vaccine matter over the East, ix. 536; causes his family to be vaccinated, xii. 447, 450; account of his travels in Greece, 448.

ELGIN, in North America, account of the botanical garden established there, xx. 18; catalogue of the indigenous and exotic plants growing there, xxvi. 162; statement of particulars relative to that institution, 163.

ELLICOTT, Andrew, his account of an epidemic in North America, xvii. 127.

ELLIOT, Mr. his account of a particular sensation of the eye, ii. 229.

ELLIS, Daniel, his enquiry into the changes of the atmosphere by vegetation, xix. 371; remarks on the theory of respiration, 547; account of his disquisition on the phenomena of vegetation, xx. 6; his reply to Dr. Bostock, 181; review of his hypothesis on vegetation, xxvii. 17; his theory on the agency of light, 18; on the nature and cause of accidents which are ascribed to the wind of a ball, 323; his opinion controverted, xxviii. 142; farther objections to his explanation of that phenomenon, xxix. 163; xxx. 246.

ELM, observations on diseases which affect that tree, v. 492; botanical description of the common, xii. 230; benefits of its bark, ibid.

ELMER, Dr. his case of mortification of the uterus, xviii. 344; account of a monstrous fœtus, 345; remarks on his case of mortified uterus, 419.

ELONGATION, remarkable case of that of the tongue, vi. 353, 435, 522.

ELYMUS ARENARIUS, or sea lime-grass, botanical description of the, xi. 529; useful in making cordage, 530.

EMBALMING, account of the mode of performing it among the ancient Egyptians, ii. 385.

EMBROCATION, formula of an excellent one for the hooping-cough, i. 84; how improper in hernia, iv. 40.

EMBRYO, on the origin of sensorial power in the human, ii. 277; on the principle of life in the, 324; its vitality different at certain periods, 458; on the living filament in the, iii. 139; liquor found in eggs on the evolution of the, 160; on the allantois of the, 161; anatomical representations of the human, iv. 354; account of the progressive increase of it in the ovum, 555. See *Fœtus.*

EMBRYOTOMY, account of the method of delivery by, iv. 168; advantages of premature delivery to supersede the necessity of the operation, v. 40; on the danger of the operation, xxv. 387; history of a remarkable case in which it was successful, ibid.; the process described, 389.

EMERALD, new earth discovered in the, iii. 173; chemical analysis of the, ix. 475.

EMETICS, recommended in spasmodic affections of the stomach, i. 50; objections to them in cases of apparent death, 59; serviceable in hooping-cough, 84; necessary in dysentery, 274; successful in puerperal fever, 389; useful in spasmodic asthma, ii. 291; efficacious in a case of ascites, 469; beneficial in a bilious malignant fever, iii. 568; new method of preparing emetic tartar, 582; useful in difficult dentition, iv. 77; on the action of them, 224; efficacious in phlegmatia dolens, v. 96; preparation of emetic tartar from glass of antimony, 199; efficacious in a case of œdema fugax, vi. 27; advantageous in pulmonary consumption, 90; objected to in apoplexy, 549; new form of one, vii. 81; their use vindicated, 90, 151; tend to aggravate that disease, 153; impropriety and danger of them, 208; explanation on the use of them, 259; necessity of giving them to children in some cases, 288; authorities in favour of them in apoplexy, 329; reasons for their use, 330; in what respects they may be safely adopted, 398; justification of them, 456; beneficial in a case of apoplexy, 514; recommended in affections of the intestines, viii. 13; on their effects, 67; their use in apoplexy questioned, 79; vindicated, 81; method of preparing emetic tartar, 92; fatal in apoplexy, ix. 411; their beneficial influence in puerperal fever, x. 15; how far serviceable in the fevers of hot climates, 38; serviceable in catarrhal fever, 130; the early exhibition of them in typhus recommended, 186; useful in the influenza, 193, 196, 198; Dr. Cullen in favour of the use of them in apoplexy, xi. 79; curious effect of emetic tartar injected into a vein, 190; found useful in epidemic fevers, 313; in diarrhœa, xii. 52; the roots of the primrose a safe one, 127; new method of preparing emetic tartar, 189; the berries of the spindle tree an emetic, 223; roots of the violet both an emetic and cathartic, ibid.; useful in poisons, 387; beneficial effect of them in the malignant fever at Gibraltar, xiii. 195, 203; their utility in an epidemic at Canterbury, 348; their powerful action in fevers, 474; how to treat patients after taking, 475; efficacious in the catarrhal fever at Canton, 489; their use in chorea, xv. 122; the pepper stone-crop operates as an emetic, xv. 265; benefit of the asarum, 268; case of croup successfully treated with, 382; a potent remedy in malignant fever, 453; ineffectual in chronic croup, 509; on their uses in diseases of the stomach, xvi. 425; on the action of, 426; mischievous in apoplexy, 427; how admissible in croup, 430; objected to in thoracic affections, 431; diseases in which they are serviceable, 432; the lesser spearwort a powerful one, xvii. 372; on the use and abuse of them in diseases of the stomach, xviii. 18; emetic properties of the dittander, 174; on their use in chorea sancti viti, 214; horse-radish serviceable as, 272; remarkable qualities of the milk-wort as an emetic and diuretic, xix. 73; decoction of the eupatorium a violent one, 262; on the use of them in pulmonary consumption, 337; cases in which they were successful, 339, 344; chamomile a good one, 459; powerfully effectual in scarlatina, xxi. 255; on their efficacy in hooping-cough, xxii. 71; how serviceable in febrile diseases, 463; injurious in remittent fevers, xxiii. 498; their utility in disorders of the stomach, xxiv. 251; on the inability of producing any effect by them in horses, 271; to be cautiously used in gout, xxvi. 481; their utility in croup, xxvii. 206; reprobated in apoplexy, xxviii. 219; useful in paralysis, 453; their use in apoplexy said to be sanctioned by experience, xxix. 15; yet condemned as fatal by Portal, xxx. 370; their effect in a case of ischuria, xxxi. 109; efficacious in severe ophthalmia, 272; their effects when injected into the veins, xxxii. 343, 429; successfully exhibited in diabetes mellitus, 446; effectual in scarlatina, 484; improper in yellow fever, xxxiii. 5; seldom used by the Chinese, 247; recommended in rabies contagiosa, 296.

EMHOFF, M. his experiments on the medical use of galvanism, x. 59.

EMIGRATION, shocking picture of Irish, vii. 381; miseries sustained in the voyage, 382; state of emigrants in America, ibid.; pestilence engendered in a ship crowded with passengers, 383.

EMMENAGOGUES, the madder formerly reckoned among the number of, xii. 29; the centaury recommended as, 221; the seeds of wild carrot serviceable as, 232; the pimpinella an excellent one, 556; the asarabacca said to possess that virtue, xv. 268; spearmint a powerful one, xvii. 559; pennyroyal a useful one, 561; the white horehound recommended, 564; marjoram in use as such, 565; the tansy extolled as an excellent one, xix. 354; the feverfew a good one, 457.

EMOLLIENTS, utility of joining them with tonics in the relief of the antagonist muscles, iii. 254; parieteria officinalis improperly considered in the number, xii. 31; the leaves of the white mullein recommended as an excellent one, 130; the seeds of the flax useful in that capacity, xiv. 162.

EMPEDOCLES, the sexual system of plants maintained by, xxvi. 293.

EMPETRUM, or black-berried heath, botanical description of the, xi. 530; said to occasion head-ache, 531.

EMPHYSEMA, observations on the disappearance of, iv. 84; two cases of its spontaneous occurrence cured by cold bathing, xxviii. 141.

EMPIRICISM, science of medicine improved by, i. 133; on the most effectual means of checking its progress, 337; on the encouragement of, ii. 150; suggestions for the remedy of the evil, 274; extract from an old statute against, 392; charged upon Dr. Beddoes, iii. 380; on the pernicious effects of, 433; ignorance the cause of its success, 523; plan for correcting the abuses of, iv. 17; on the proper methods of exposing, 230; mode of checking it in Russia, 405; account of a German nostrum for pregnant women, v. 398; necessity of repressing the evil of, vi. 129; plan for remedying, 338; account of a nostrum for scarlet fever, 367; caused by the prostitution of medical degrees, vii. 13, 161; scandalous practices in, 370; mischiefs of domestic quackery, 392; curious anecdote of, 396; on the causes of its prevalence, viii. 246; proposed remedy, 249; on the means of suppressing, 461; on the encouragement of it in England, x. 261; successful in America, ibid.; quack medicines should be discouraged by the faculty, xi. 183; historical accounts of, 554; causes of it in England, 555; singular account of an American adventurer, 556; historical account of it in England, xii. 137, 212, 423; anecdotes of, 428; singular history of, xiii. 258; on the origin and progress of, xiv. 437; account of an ancient empiric, 442; inadequacy of the measures proposed for remedying the evil of, xv. 258; encouraged by the people, 259; its prevalence in France, 334; historical view of its origin and progress, 362; anecdotes of Michael Schuppach, 364; pleasant story of an urine-caster, 365; account of Christopher Ozanne, a French quack, 366; danger produced by popular treatises on medicine, 369; historical notices of it in England, xviii. 3; mischiefs arising from, 286; necessity of enquiring into the state of irregular practice in medicine, xxi. 101; on the proposed means of remedying the evil of irregular practice, xxiii. 63; legal opinions on the same, 65; on the fashion for it among ladies, 211; mischief produced by water doctors, 378; particularly dangerous in disorders of the eyes, xxv. 75; method proposed for remedying, 110; animadversions on the plan for checking, 493; xxvi. 124; on the disposition to, xxvii. 31; extraordinary price given for De Bruyn's secret in midwifery, 118; tricks played by quacks to impose upon credulity, 348; its effects on regular practice, xxviii. 405; means of checking its progress, 407; facilities held out for, 464; a new discovery propagated in France called aphrodisiacs, 476; the scandalous encouragement which it has received from regular physicians, xxix. 2; on the empirical remedies of gold, 13; remarks on its utility, 119; facilities furnished for quackery by the Scotch universities, 206; evil of ignorant persons undertaking medical practice, 280; punishments inflicted upon quacks in England, 308, 311; remarkable instances of it, xxx. 2; examination of Goderneau's powder, 222; the want of medical reform to cure the evil of it, 452; on the facility of obtaining degrees, 471; caused by defects in the examinations of surgeons, 479; prevalence of it in disorders of the eyes and ears, xxxi. 257; necessity of legislative interference to remedy the evil, 477; the ergot used to expedite labour by empirics, xxxii. 93; remarks on the various forms of quackery, xxxiii. 20; account of the practices of the Chinese, 247; scandalous increase of it in England, xxxiv. 188; remarkable anecdotes of it, 189; facilities to medical practice in England, 190.

EMPLASTRUM GUMMOSUM, objections to the term in the Pharmacopœia, xi. 300; vindication of the use of it, 536.

EMPYEMA, history of a remarkable case, xiii. 21; singular case of, xviii. 392; the operation successfully performed in a case of hydrothorax, xxiv. 517; case of vomica cured by the operation for, xxxii. 120.

EMULSIONS, the oil of amber a good one in hooping-cough, i. 178; account of useful ones in various complaints, vi. 70; a diuretic one in the seeds of burdock, xix. 259.

ENAMEL, that term improperly applied to the teeth, viii. 559; experiments on that of the teeth, xvi. 95.

ENCYCLOPÆDIA, account of one for chemistry, iv. 74; announcement of a new medical one, v. 493; another for manufactures and useful arts, xviii. 579.

ENDEMICS, the intermittent fever properly classed under that denomination, i. 236; caused by argillaceous earth and clay soils, 258; general observations on them, 467; said to be most prevalent where vegetation is luxuriant, 468; remarkable histories of, 470; usual symptoms produced by them, 477; observations on those of India, ii. 4; account of a remarkable one in Norway, vi. 13; the ophthalmia in Egypt endemic, 357; observations on the endemic causes of disease, viii. 221; the plague considered under that character, 450; inquiry into the original causes of endemics, xviii. 86; remarks on those of Egypt, xix. 114; characteristics of the endemic air, 199; how occasioned in crowded cities and towns, 200; how caused in volcanic countries, 201; instance of an endemic cynanche parotidea, xx. 180; observations on their causes, xxi. 16; remarks on those of Egypt, 107; on the prevailing ones among the troops in the island of Walcheren, xxiii. 183; observations on the endemics of Bengal, xxx. 229; on those of Batavia, 231; cretinism said to be endemic, xxxiii. 282.

ENEMATA, on the use of them in gastritis, iii. 229; beneficial in fever, x. 39; observations on the effects of the tobacco when used as such, xxv. 224, 225; lemonade serviceable in that capacity for allaying thirst and relieving febrile irritation, xxvii. 267; benefit of the ipecacuanha when used as such in dysentery, xxviii. 93; dangerous effects of the tobacco in some cases, xxx. 388; retention of urine relieved by the same, xxxii. 520.

ENGELBRECHT, Hans, account of his pretended visions, xxix. 408.

ENGLAND, causes of the salubrity of the climate of, i. 254; observations on the diet of its inhabitants, iii. 251; on the encouragement of quackery in, iv. 406; on the progress of science in, vi. 408; on the prevalence of empiricism in this country, and the causes, x. 261; healthiness of the climate, xi. 284; on its population, 365; difference of temperature observable in, xxi. 4; its local peculiarities, 5; its freedom from poisonous plants and animals, 6; on the insects peculiar to, 7; its medicinal productions, 9; catalogue of medicinal plants growing in, 11; its mineral productions, 14; difference in the duration of life in, 15; on the diseases common in different districts, 91; great inundation on the eastern coast of, xxiii. 282; accounts of the epidemic in various parts of, 283; the rot in sheep first observed here in 1274, ibid.; account of the plague which occurred in 1348, ibid.; an epidemic madness in, 284; on the sweating sickness of 1485, ibid.; a malignant catarrh prevalent in 1517, ibid.; account of the great plague of 1665, 285; epidemic prevalent in 1734, 286; comparative state of its population, xxvii. 169; statistical inquiry into its population, xxviii. 307.

ENGLISH, Mr. his case of the efficacy of stramonium in asthma, xxvi. 490; his case of injury done to the fœtus though the mother was not affected, xxx. 510; his case of malformation of the heart, xxxii. 516; on the use of ipecacuanha and opium in dysentery, xxxiii. 146.

ENOMETER, description of an instrument so called for determining the quantity of saccharine matter in wine, xx. 482.

ENTERITIS, on the benefit of enemata in, iii. 229; observations on the nature of the disorder, viii. 472; appearances in cases of, xii. 498; case, with remarks, xiii. 182; remarks on the proper treatment of it, xx. 92; cured by bleeding, xxii. 85; relieved by blisters, xxiii. 83, 176.

ENTEROCHUS, experimental observations on that species of fossil, xxviii. 306.

ENTERODYNIA, singular case of, xix. 524.

ENTHUSIASM, remarkable history of it in one family, viii. 311; singular case of abstinence caused by, xv. 511; justifiable in botanical pursuits, xx. 11; remarkable history of Hans Engelbrecht, xxix. 408; surprising instances of religious frenzy in Cornwall, xxxi. 373; that account controverted, 464; the statement vindicated, xxxii. 32.

ENTOMOLOGY, society instituted in England to facilitate that study, xxiii. 262.

ENULA HELENIUM, efficacy of it in cutaneous diseases, i. 298.

EOSACO, his case of vaccine eruption, xiii. 399; observations explanatory of the case, 539.

EPIDEMICS, dreadful one in North America, i. 78; successful method of treating, 79; general history of, ibid.; account of one of the eyes, 234; observations on an epidemic catarrh, 340; on rheumatic ones, 384; a remarkable one among cats, 412; observations on the history and cure of, 467; enquiry into the causes of, ii. 178; observations on one at Birmingham, iii. 94; ravages of one at Strasburg, 377; why seldom observed in medical practice, 392; brief history of, 559; observations on autumnal, iv. 490; comparison of different, vi. 265; utility of publishing accounts of epidemic diseases, vii. 285; a pestilential one in England in 1545, 311; observations on pestilential, 312; how dependent upon the nature of soils, viii. 222; their causes ascertained, 224; on the frequency of them in Lincolnshire, 225; account of one in the French army in Syria, 451; on the epidemic scarlet fever and sore throat, ix. 157; alarming one at Paris, 287; farther particulars of it, 419; on the epidemical catarrhal fever, 377; observations on those of London, 385; communications on, 460, 470, 505, 509, 512, 515, 517, 522; general remarks on that of 1803 compared with former ones, 507; additional communications, x. 46, 73, 80; collection of papers transmitted to Dr. Beddoes from various correspondents, 97, 127, 193, 231, 289, 303, 385, 517; account of one at East Bourn, 127; at Plymouth, 129; observations on one at the same place in 1733, 135; not contagious, 142; account of one in 1580, ibid.; historical notices of, 144; on the re-appearance of, 177; observations on one at Aberdeen, 232; at Dumbarton, 233; at Rumsey, in Hampshire, 312; in Yorkshire, 355; account of one at Wilmington, in America, 365; prize question on, 383; account of four at Charlston, in America, 470; the measles considered as one, 489; contagious influence of the influenza observed at Alcester, xi. 81; on the prevalence of, 82; observations on those of 1802 in America, 309; observations on the epidemic catarrh at Bath, 488; observations on the true nature of, 513; remarkable one among sheep, xii. 456; on the origin of an epidemical fever in Spain, xiii. 103; proposals for preventing the progress of, 114; how to prevent their recurrence, 116; on the extensive range of one, 213; observations on one in Scotland, 216; outline of a plan for preventing, 272; origin and progress of one in North America, 304; account of one in the barracks at Canterbury, 348; enquiry into the particulars of one in France, 383; how far dependent on the state of the atmosphere, 467; observations on one in China, 487; description of one at Gibraltar, xiv. 200; on the symptoms and treatment of the same, 202; use of cold water in epidemic ophthalmia, 239; farther particulars of one in North America, 312; observations on the epidemical fever of Spain, 337; report of the London Institution for preventing, 340; prize question proposed by the Medical College of Berlin on, 381; means adopted in Prussia for preventing, 382; instinct of sparrows in discovering, ibid.; outline of a treatise on, 575; observations on one in Jamaica, xv. 41; on the discovery of the efficacy of nitrous acid in preventing, 374; account of a malignant fever at Quebec, 446; of another at New York, xvii. 97; observations on the different kinds of, 103; observations on the epidemic ophthalmia, 193; observations on that which occurred at Gibraltar in 1806, xviii. 9; in what respects connected with the state of the atmosphere, 86; alarm excited by one at Marseilles, 119; treatise of Dr. Mead composed in consequence, 120; mistakes in respect to, 425; supposed to be caused by volcanic eruptions, 500; account of an epidemic ophthalmia at Edinburgh, 578; most fatal in places subject to endemics, xix. 115; one ascribed to inflammation of the brain, 169; one among cats, 171; occasioned by vitiated air, 199; on the seasons peculiar to, 317; generally produced by want of cleanliness among the poor, 570; historical notices of remarkable, xx. 27; enquiry into the causes of, 28; account of one at New York, 30, 34; on the influence of the atmosphere in generating, 366; on that of Gibraltar, 368; on the sources of epidemic diseases, 370; in what respects depending on the state of the air, xxi. 16, 91; on those of hot climates, 107; on the disappearance of other disorders during the prevalence of epidemics, 109; observations on those of America and the West Indies, 241, 247; on the epidemic measles of 1808, 359; remarks on the same, 366; on contagion as confounded with epidemics, xxii. 105; on the epidemic ophthalmia, 116; account of a peculiar one in Barbary in 1799, 193; seclusion beneficial in, 229; account of an epidemic ophthalmia, 260; historical account of the leprosy, 107; observations on, 513; account of a remarkable one in France, xxiii. 33; danger of sudden changes in, 40; method of treatment, ibid.; means of preventing their propagation, 41; remarks on the epidemic ophthalmia, 77; on the prevalent ones at Madeira, 103; benefit of smoaking tobacco against, 118; remarkable one in Grenada, 166; on the contagious property of the hooping-cough, 177; historical particulars of ancient ones, 281; commonly follow earthquakes and volcanic eruptions, 282; fatal one in England, A. D. 1196, 283; another all over Europe, in 1348, ibid.; an epidemic madness in 1354, 284; account of the sweating plague of 1485, ibid.; again in 1517, ibid.; a remarkable pestilence throughout Europe in 1600, ibid.; the yellow fever breaks out among the Indians of North America in 1619, 285; account of the great plague in London in 1665, ibid.; fatal fevers in Ireland in 1719, 286; an epidemical catarrh nearly over all the globe, ibid.; general causes of, 287; view of the fever of Walcheren and its consequences, 418; history of an epidemic disease in France, 455; how produced by convulsions of the earth, 472; and by bad air, 474; affected by changes of the moon, 476; observations on the epidemics of marshy countries, and that of Walcheren in particular, xxiv. 22; attributed to animalculæ in the atmosphere, 23; account of the epidemic dysentery among the troops at the Cape of Good Hope, 161; queries respecting the epidemical ophthalmia in the British army, 317; history of one prevalent in Orange county in the state of New York, 365; how produced by the effluvia of putrid animal bodies, 422; account of the sickness in the American army at New Orleans, 435; statistical report of the Walcheren fever, xxv. 1, 13; benefit of tobacco in, 136; observations on the epidemical catarrh or influenza as it has appeared at different periods, 145; history of an epidemic puerperal fever, 193, 198; account of those of South America, 241; account of an epidemic fever in New England, xxvi. 217; on the causes and effects of marsh miasmata, xxvii. 37; report on those of Edinburgh in 1810, 167; croup and erysipelas alleged to be epidemical, 362; on the epidemic fever of hospital ships and jails, 378; account of an epidemic disorder in the army of Portugal, xxviii. 67; on the epidemic constitution of the atmosphere in 1810, 137; report of the Board of Health at New York on, 145; account of a spotted fever in New England, xxix. 328, 331; on the yellow fever of New York in 1803, 416; on the marsh fever of Walcheren, xxx. 15; occasioned by exhalations, 159; on those of Bengal, 228; observations on a fever in the Mediterranean, 246; account of a fever at Gibraltar, xxxi. 258; account of an epidemic disorder of the eyes, 264; a remarkable one in Cornwall, 375; observations on that case, 464; on the epidemic fevers of the Mediterranean, 489; vindication of the account of the epidemical enthusiasm in Cornwall, xxxii. 32; instance of epidemic pemphigus, 165; on an inflammatory fever in Portugal, 267; instances of pemphigus being epidemic, 287; account of a remarkable one in New York and New England, xxxiii. 92; remarkable one among cattle, 161, 401; account of an epidemic fever at Cambridge, 338, 429; account of an epidemic fever at Gibraltar in the years 1804, 10, and 13, 414; instance of pemphigus being epidemical, xxxiv. 171; on the fever prevalent in Guzerat, with remarks on the diseases of India, 243; on the fevers of Seringapatam, 244; answers to queries relative to the epidemic of Gibraltar, 301; account of one at Paris, 338; reports on those of Spain, 406, 498; frequent in hospitals, 487.

EPIDENDRUM FRAGRANS, correction of an error in the representation of that plant, xxxii. 85.

EPIDERMIS, most conspicuously seen in the human skin, i 244; separated into two parts, ibid.; chemical observations on the, ibid.; action of lime water on the, 245; description of the, iv. 9; effects of galvanism on the fingers when deprived of the, vii. 242; has but a small portion of vitality, xvi. 521; that of the leech different from other animals, xxvi. 410; remarkable state of it in a man whose skin turned black, xxxiii. 74.

EPIGASTRIUM, pulsating tumour in the, mistaken for aneurism, xviii. 485; on preternatural pulsation in the, xxii. 505; on a strong pulsation of the aorta in the epigastric region, xxxi. 159.

EPIGASTRIC ARTERY, inosculation of the axillary with the, ix. 177; found divided in crural hernia, xi. 125; safety of cutting the, 374.

EPIGLOTTIS, memoir on its use in deglutition, xxxi. 318; the larynx may subsist without one, 463; lost by the venereal disease, 464; its appearance in a case of laryngitis, xxxiv. 256.

EPILEPSY, account of various medicines successfully employed in, i. 179; analysis of a powder used in the cure of, 180; efficacy of the misletoe in, ibid.; benefit of nitrat of silver in, 181; on the causes of the disease, ibid.; cases of the efficacy of nitrat of silver in, 222; induced by excessive sleep, 259; benefit of the datura stramonium in, 279; farther account of the effects of nitrat of silver in, ii. 70; on the use of belladonna in, 72; cured by extractum taxi, 85; efficacy of the digitalis in a case, 118; case treated with nitrat of silver, 173; topical applications found beneficial in, iv. 380; failure of practice in the treatment of, 417; the nitrat of silver ineffectual in that disorder, 418; observations on its effects, 419; cured by opium, 570; success of electricity in, v. 347; narrative of the insufficiency of nitrat of silver in, 435; unreasonableness of looking for a specific in, 502; detail of unsuccessful cases of, 503; effects of argentum nitratum in, ibid.; delirium produced by, vi. 79; two species of, 377; use of topical anti-spasmodics in, ibid; cases of it connected with catalepsy, 425; distinguished from apoplexy, vii. 364; on the characteristics of, 441; beneficial effects of galvanism in, 538; history of a case cured by caustic application, x. 51; occasioned by a tumour of the leg, 432; efficacy of the valerian in, xi. 446; case of vertigo and epilepsy occasioned by an insect, 450; flowers of the Galium serviceable in, 533, 534; benefit of the misletoe in, xii. 33; an empyeumatic oil efficacious in, 37; on the suddenness of its occurrence, 112; case of it, ibid.; datura stramonium efficacious in, 131; the hyoscyamus administered in, 132; benefit of the viola tricolor in, 224; the cow-parsnip serviceable in, 364; occasioned by parsley, 558; efficacy of the sugar of lead in the cure of, xiv. 10; case of its succeeding a fit of anger, 275; efficacy of the sedum acre in, 430; success of galvanism in, 527; farther instances of the same, xv. 150; empirical remedy for the, 355; common in the South Sea Islands, xvi. 174; emetics serviceable in, 432; cured by trepanning, 473; on the distinction of chronic and acute, xvii 390; on the virtue of misletoe in, xviii. 17; benefit of digitalis in, 172; flowers of the cardamine serviceable in, 273; efficacy of sugar of lead in, 355; benefit of acetate of lead in, xix. 70; case of it produced by worms, 305; observations on the same, 308; supposed to be sometimes hereditary, xxi. 231, 238, 282; state of the brain in, xxii. 485; sometimes takes the appearance of tetanus, xxiii. 16; how to be treated when the brain is injured, 17; observations on the aura epileptica, 18; case of epilepsy, in which the recurrence of the paroxysms was prevented by cold affusion, 20; successfully treated by opiate frictions, 74; benefit of bleeding in, ibid.; fatal case of, 480; attributed to animalcula, xxiv. 26; the sanguinaria Canadensis useful in, xxv. 167; remarkable history of a case of, 179; supposed efficacy of tobacco in, 227; plumbi acetas given with success in, 456; physiognomic appearances of epileptics, xxvii. 40; observations on the facial angle in, 41; arsenic a remedy for, xxix. 13; benefit of the oil of turpentine in relieving that disease, xxx. 509; benefit of the nitrate of silver in, xxxi. 148; case resembling epilepsy occasioned by a spider in the ear, 172; case of epilepsy consequent on amenorrhœa, 264; caused by terror, 378; occasionally ceases spontaneously, xxxii. 293; observations on the cerebellum and different parts of the brain in epileptics, xxxiii. 182, 428; necessity of inquiry into the history of this disease, 186; effects of cold in producing, 246; an hereditary disease, 435; dissection of the head of a patient who died in it, 516; cured by purgatives, 519; cured by charms, xxxiv. 85; benefit of oil of turpentine in, 248; benefit of cathartics in cases of, 517; frequently ceases spontaneously, 518.

EPILODIUM, botanical description of that plant, xiv. 171; used in brewing, ibid.; employed by the Russians for intoxicating purposes, xxi. 14.

EPIPHORA, or involuntary flowing of tears, described, xxvi. 230.

EPISTAXIS, two cases of, vii. 332; observations on them, viii. 68; singular case of profuse, xi. 549; discovered to be a leech in coagulated blood, 550.

EPIZOOTICS, remarkable one among cows near London, i. 11; memoir on one among cats, 412; iv. 373; on the hydrophobia considered as, ii. 174; observations on the little attention paid to, xix. 170; account of one in the canine species, xx. 468; remarkable one among the horned cattle in Grenada, xxiii. 166; another in Barbadoes, 168; observations on the communication of diseases from one species to another, 169; account of one in Sweden, 430; severe one among horned cattle in France, xxxiii. 161, 401.

EPOCHS, memorable ones of the plague, iv. 365.

EPSOM SALTS, method of preparing, xxvi. 251; a remedy in gout, xxxii. 200, 201.

EQUATIONS, new method of freeing them from surds, xxx. 426.

EQUATOR, observations on the diet of people living in countries near the, iii. 250.

EQUILIBRIUM, observations on that of liquids and solids, xxviii. 415.

EQUISETUM, or corn horsetail, botanical description of that plant, xix. 539; its astringent and diuretic qualities, 540; description of the equisetum hyemale, or rough horse-grass, ibid.

EQUIVALENTS, description of a new scale for chemical, xxxi. 167.

ERASISTRATUS imputes inflammation to a transfusion of blood, ii. 338.

ERDMAN, Dr. his account of the effects of anger in a boy, ix. 288; on the use of spongia usta in glandular diseases, 486.

ERGOT, dissertation on the natural history and medical effects of the, xxxii. 90; generated in rye by disease, 91; deleterious in bread, 93; used to expedite parturition, ibid.; violence of uterine effects produced by it, 94; caution in the use of it, 96; beneficial in amenorrhœa, 97; its operative powers confined to the uterine fibres, 98; descriptive plate of it, with references, 99; instance of the bad effects of it, 192.

ERICA, or common heath, botanical description of, xiv. 541; applied to various purposes in the Highlands of Scotland, 542; honey extracted from the flowers, ibid.; account of the erica monadelphia, a new species, xxvi. 425; description of the erica odorata, 427.

ERIOSPERMUM LATIFOLIUM, description of that genus of plants, xxvi. 426.

EROSION, on the difference between perforation and, xxxi. 79.

ERRHINES, the root of the iris a dangerous one, xi. 449; efficacy of the powder of the convallaria to excite sneezing, xiv. 66; formulary of one for the eyes, xv. 151; benefit of the asarum as a sternutatory, 269; the wood betony serviceable in promoting sneezing, xvii. 563.

ERRMAN, M. his galvanic experiments on water, xvii. 295.

ERUPTIONS, representation of those on the arm of a milker the tenth day of the cow-pox, i. 3; use of hot flour externally applied in cutaneous, 84; minute account, and an engraved representation of those of vaccine inoculation, 118; farther observations on the benefit of hot flour in, 152; account of one observed after vaccination, 217; on those of the cow-pox as distinguished from the small-pox, 232; efficacy of the enula helenium in, 298; remarkable case of petechiæ, 420; ancient account in Welsh of the great pustulous one, ii. 272; observations on the same, 352; farther account of it, 416; benefit of a new salt in herpetic eruptions, iii. 85; on those of the vaccine disease resembling small-pox, 92, 97; inquiry into the true nature of those which appear after vaccination, 247; case of miliary eruption in puerperal fever, 265; on the pemphigus major, 552; benefit of belladonna in cutaneous, iv. 7; on those of the vaccine disease, 464; beneficial effects of the sulphuric acid in chronic, 482, 487; on chronical ones in scrofula, v. 490; case of an inveterate one caused by Jesuit's drops, 519; remarkable case of lepra græcorum, with a plate, vi. 125; cases of hæmorrhœa petechialis, 234; efficacy of coccinella in, 235; cases of vaccine, 236; on those in dolor facei, 244; on those of the cow-pox, vi. 253; observations on pestilential, 260; benefit of the rhus radicans in herpetic ones, 273; general classification of, 297; definitions of the different kinds of them, 298; the several orders, 299; representations of those of the cow-pox inoculation, 386; those of the vaccine virus confounded with other cutaneous ones, vii. 172; representation of those in the udder of a cow, 185; the eruptions in vaccination not the effect of inoculation, 187; remedy for psora, 520; on cutaneous and scurfy ones of the head, 521; advantages of mercury in, ibid.; remarkable case of, viii. 106; observations on that case, 200; remarkable history of lusus naturæ, 509; case of inveterate tinea capitis, 554; on the treatment of tinea capitis, ix. 61; good effects of walnut oil in herpetic ones, 94; on those of the scarlet fever, 159; union between elephantiasis and herpes, 289; description of those produced by the inoculation of the goat-pock, x. 344; on the petechia without fever, 444; cured by rhus radicans, 480; on those of the nettle rash, 481; description and causes of, 482; on those produced by the poison of muscles, 483; remarkable appearance in a child, 484; difference between urticaria and the itch, 485; on those of the measles, 487; history of the blue disease, 575; benefit of suroxygenated muriatic acid in cutaneous, 576; flowers of zinc recommended in, xi 93; remarks on herpetic eruptions, with a coloured representation, 230; generic character, ibid.; combined with other eruptions, 233; division and description of the species, 234; herpes phlyctænoides, 235; herpes erysipelatosus, 237; herpes miliaris, 239; on those after measles, 315; on those of the small-pox, 321; efficacy of hemlock in, 343; a confluent one after vaccination, 385; account of pustulous ones in poultry, 431; the same in swine, 432; virtue of common scabious in, 532; benefit of the galium in, 534; correction of an error respecting those of animals, 553; on the modifications of the vaccine, xii. 97; observations on cutaneous, 193; on those of the cow-pox, 195; efficacy of the viola tricolor in cutaneous, 224; benefit of the bark of the elm in removing, 230; on those which occur after vaccination, 358; benefit of hemlock in, 362; creeping water-parsnip serviceable in, 366; the œnanthe crocata beneficial in, ibid.; water-hemlock recommended in, 367; case of leprosy, 404; benefits of blisters in, 405; peculiar kind of them in sheep, 460; use of the pimpinella in cleansing the skin, 557; benefit of the common black elder in, 560; description of those which succeed vaccination, xiii. 2; efficacy of adhesive plaster in those of the head, 8; observations on ring-worm of the head, 24; effect of eruptive diseases on the security of vaccination, 268; observations on herpetic, 415; on those of the small-pox, 467; effect of cutaneous eruptions on vaccine inoculation, 537; observations on the same, 541; efficacy of sugar of lead in chronic ones of the head, xiv. 10; herpetic eruption, the cause of incomplete vaccination, 54; garlic serviceable in, 65; root of the convallaria useful in, 67; on the effects of arsenic in three cases of, 113; case of eruption of the face, 116; description, with an engraving of the confluent chicken-pox, 141; on those of the sheep pox, 142; on those of vaccination, 147; efficacy of the viola tricolor in venereal ones, 431; on prurigo, 525; produced by mezereon, 544; remarkable cases of crusta lactea, xv. 33; on herpes in vaccination, 137; the cucubalus used for the cure of erysipelatous eruptions, 264; on those which sometimes occur from the use of mercury, 280; effects of Dr. Fowler's mineral solution in the cure of eruptive diseases, 297; case of their occurrence in the sore-throat, 485; on those charged upon vaccination, 519; causes of them in children, ibid.; unfairly ascribed to vaccine inoculation, 537; on the effect of herpes in vaccination, xvi. 65; on those of the small-pox, 68; cases of herpetic, 69; three divisions of cutaneous ones, 178; account of some remarkable ones after vaccination, 219; produced by the grease in horses, 245; on herpes in vaccination, 247; benefit of the celandine in, 559; the root of the water-lily effectual in the cure of leprous ones, xvii. 69; use of arsenic in removing, 254; efficacy of kali sulphuratum in leprous ones, 315; antirrhinum elatine serviceable in cutaneous, xviii. 171; epidemics said to be produced by volcanic, 500; singular case of rubeola sine catarrho, 564; benefit of fumitory in cutaneous, xix. 72; efficacy of arsenic in, 206; the hypochœris, or spotted cat's-ears, said to be serviceable in, 256; description of a remarkable eruptive disease in children, 344; on those which succeed vaccination, 380; remarkable case of eruption produced by cold, 524; description of the several varieties of, xx. 273, 371; account of urticaria evanida, 275; observations on a species of porrigo in the scalp, xxi. 132; sometimes hereditary, 330; account of an eruptive disease after vaccination at Sherborne, xxi. 250; account of that of Mount Etna in 1809, xxii. 175; remarkable ones of Hecla, in Iceland, xxiii. 283, 284; account of an eruptive disease of the penis, with a coloured plate, 441; observations on the effects of volcanic ones, 470; danger of curing certain eruptions, xxiv. 125, 214, 219; characteristics of the itch, 127; cases of herpes excedens vermiculatus, 162; account of an inveterate species of psora at Malta, xxv. 429; preparations for herpetic eruptions, 498; remedy for the favus, ibid.; description of those which appear in a petechial epidemic, xxvi. 220; description of the eruption in the erythema mercurialis, 341; observations on herpes of the prepuce, 495; produced by the eau medicinale, 500; eruptive diseases common in Iceland, xxvii. 120; on those of a venereal character, 143; narrative of a volcanic eruption in the sea, off St. Michael's, xxviii. 200; observations on mercurial ulcers and eruptions, 511; eruptive diseases frequent in Ceylon, xxix. 163; account of the volcanic eruption in the island of St. Vincent, 426; use of arsenic in herpetic eruptions, xxx. 23, 296; classification of cutaneous eruptions, 414; account of lepra, 418; benefit of arsenic in, 422; fatal case of pemphigus, xxxi. 265; discovery of sulphurated soap for the itch, xxxii. 79; variolous ones found in the trachea, ibid.; case of eruptive affection, 102; on the eruptions in the itch, 142; instance of epidemic pemphigus, 165; the hepar sulphuris successful in tettery affections, ibid.; eruptive disease prevalent at Paris, 256; historical researches on pemphigus, 281; case of leprosy in a boy, 355; ointment recommended in, 356; lepra and psoriasis not essentially different, ibid.; case of psoriasis diffusa, 357; cases of pemphigus, 397, 398, 400; on the difference of opinion respecting the cause of the itch, 496; description of porrigo, xxxiii. 130; varieties of that complaint, 131; benefit of viola tricolor in that disorder, 132; on porrigo lupinosa, ibid.; on porrigo scutellata, 133; perrigo decelvuus, ibid.; description of the impetigo or running tetter, ibid.; divisions of this genus, 135; washing and bark necessary in, 136; Chinese method of treating eruptive diseases, 248; history of tubercular eruption of a syphilitic appearance, 410; the itch produced by an insect, 515; new method of treating that disorder, 516; on the infectious nature of cutaneous eruptions, xxxiv. 98; case of pemphigus, 171; on venereal and those which are supposed to be syphilitic, 229, 245, 265, 455; the hepar sulphuris a remedy in tettery eruptions, 257; analogy of those of small-pox and vaccination, 295, 387.

ERYNGIUM, or sea-holly, botanical description of the, xii. 231; the root made into a candy, ibid.; description of the eryngium corniculatum, xxvii. 259; a native of Portugal, ibid.

ERYSIMUM, botanical description of the varieties of, xviii. 364; benefit of the erysimum officinale, or hedge mustard, in ulcers of the throat, ibid.; use of the erysimum barbarea, or water cresses as a salad, 365; benefit of erysimum alliaria in gangrenous ulcers, 365; use of erysimum cheiranthoides, or treacle wormseed, as a vermifuge, 366.

ERYSIPELAS, benefit of hot flour externally applied in, i. 84, 152; an empirical remedy for, 154; on the appearances of it in vaccination, ii. 97; objections to cold applications in, 280; definition of the disease, iv. 301; occasioned by the stings of insects, ibid.; said to be contagious, ibid.; carbuncles of the plague of the nature of, 302; cases of typhus, ibid.; on that of the face, 303; on artemesia in, ibid.; how reckoned among the exanthemata, v. 484; on that of new born children, 511; produced by the action of the metallic brush, vi. 91; case in which it was followed by general anasarca after vaccination, 131; use of ammonia and aromatic confection in, viii. 473; its appearance under a variety of forms, ix. 13; uniformly a disease of debility, 14; bleeding improper in, 15; combined with herpes, xi. 233; description of the erysipelatous herpes, 237; efficacy of hemlock in, 343; a tumour produced by hemlock, xii. 368; the flour-bag recommended in, 406; benefit of cold applications in, 523; form of a lotion for, 524; cases successfully treated on that plan, ibid.; observations of Hippocratès on, 525; benefit of the black elder in, 560; remedy used in Gothland for the, xv. 264; similarity between scarlatina anginosa and, 492; emetics serviceable in, xvi. 432; want of sufficient attention to this disease, xix. 280; method of treatment, 281; cured by cantharides, xx. 296; produced by diseased liver, 342; a febrile disease, 371; characteristics of the, ibid.; on the erysipelas phlegmanodes, 373; symptoms of, 374; on the erysipelas erraticum, 375; treatment of erysipelas œdematodes, 376; utility of bark in, ibid.; caused by stricture of the urethra, xxii. 135; transition from erysipelas to the gout, xxvi. 198; advantage of cooling lotions in, 433; supposed to be contagious, xxvii. 362; facts relative to an erysipelas on board the Jalouse, xxxi. 441; observations on the symptoms of, 446; mode of treatment, ibid.; case in which it was complicated with pemphigus, xxxii. 285; said to be contagious, xxxiii. 247; advantage of the treatment of erysipelas by incision, 506.

ERYTHEMA, description of that order of cutaneous diseases, xx. 281; of the erythema tuberculatum, ibid.; case of erythema not occasioned by mercury, xxi. 417; a severe case unconnected with mercurial action, xxviii. 61.

ERYTHEMA MERCURIALE, history of three cases of, xiii. 177; essay on that complaint, xv. 280; case of, xxvi. 341; uncommon in India, ibid.; a case in which it was accompanied by an affection of the cornea, xxvii. 420; the term not sufficiently characteristic of the eruption described, xxviii. 512.

ESCHAROTICS, ganglia successfully treated with, xxiii. 432; the Sanguinaria Canadensis, or blood-root, effectual on fungous flesh, xxv. 167.

ESCHENMEYER, M. his observations on sensations after decapitation, i. 52.

ESDRAS, the Chaldaic language introduced by, i. 438.

ESQUIMAUX, on the peculiarity of their smell, vi. 442.

ESSENES, a philosophical sect among the Jews, their tenets, i. 439.

ESSEX, on the aguish nature of that county, i. 255; proceedings of the Medical Benevolent Society of, vi. 113; on the aguish nature of the climate of, xxi. 91; note to a practitioner of, xxii. 88; observations by the same on spiders' web in ague, 218; his case of diseased scrotum, 220; reply to the Essex practitioner on cobweb in ague, 369, 373; the same in twin cases, xxv. 485; reply to him on that subject, xxvi. 115; his vindication, 282; his remedy for ring-worm, xxvii. 187; reply to a Norfolk practitioner, 188; on Clarke's Practice of Physic, 189; answer to him, 370; annual mortality in, xxx. 140.

ESTABLISHMENT, plan of a convalescent, v. 306; account of one for artificial mineral waters, vi. 185; plan of that in London for the prevention of contagious fever, viii. 538; account of one at Bristol for the sick poor, x. 571; plan of a provincial one for the propagation of the vaccine discovery, xiv. 230.

ESTLIN, J. B. his case of amaurosis relieved by blistering, xxxiv. 425.

ETHER, a re-agent in chemical process, i. 265; encysted hernia reduced by the vitriolic, 283; the same recommended in steatomatous tumours, ibid.; alcohol not essential to the formation of, 301; its effects on unguentum hydrargyri, 359; its utility in biliary calculi, 394, 499; martial ether obtained from red oxyd of iron, 505; rheumatism cured by friction, with acetic, ii. 43; a child cured by, ibid.; on the inflammation of, 77; obtained from sulphuric acid, 82; elastic gum dissolved in, 83; method of preparing nitric ether without fire, iii. 86; how to deprive it of its alcohol, ibid.; improved method of preparing the acetous, 267; another process, 268; medicinal uses of the acetous, iv. 82; on the effects of acetic ether in rheumatic complaints, 369; on the formation of, 377; its good effects combined with opium, in cramp, 444; new method of preparing muriatic, vi. 16; efficacy of acetic ether in rheumatic affections and gout, 273; its effects on germination, vii. 471; its action in water, 573; new method of preparing phosphoric, viii. 569; mode of keeping phosphorus in

2

sulphuric, ix. 299; effects of the phosphorated ether in paralysis, 300; new method of preparing muriatic, x. 94; memoir on the means of obtaining muriatic, xx. 125; note on that discovery, 130; its property of producing muriatic acid when burning, 131; its use in hydrophobia, xxi. 340; improved process for preparing sulphuric, xxii. 174; farther observations on the same, 362; an excellent remedy in hydrothorax, xxiii. 334; case of strangulated hernia reduced by the administration of it, xxv. 28; elastic gum dissolved in, xxvi. 382; the only solvent for that substance, xxviii. 56; forms part of the preparation of camphor, xxix. 11; its effects on a resinous substance, xxx. 343.

ETHICS, plan of a work on medical, x. 96; review of Dr. Thomas Percival's medical, xi. 181.

ETHIOPS, new mode of procuring it, iii. 269; short and easy method of preparing it, vi. 15; experiments to prove that cinnabar and ethiops are composed of sulphur and mercury, viii. 573.

ETIENNE, Charles, professor of anatomy at Paris in the sixteenth century, account of his works, ii. 64; on the Cæsarian operation, 458; on the nerves of the voice, iv. 341; ignorant of the first cervical nerve, 342; on the intercostal nerve, 343.

ETNA, account of an eruption of Mount, xxii. 175; another, followed by the plague, xxiii. 282; violent eruptions of, 285; again in 1788, 287; electrical phenomena observed on, xxxiii. 249.

ETTMULLER, his different opinions on animal heat and inflammatory diseases, ii. 338; acquainted with Mayow's discoveries, iii. 342; ascribed infectious disorders to worms, vii. 177; his commendation of Gremb's Arbor Integra, 401; denies that acute diseases are hereditary, xxi. 332.

ETYMOLOGY, on that of diabetes, ii. 87; plan of an anatomical one, 479; observations on, xx. 535; animadversions on that paper, xxi. 89; instances of erroneous, xxii. 264; that of contagion, 513; curious instances of medical, xxv. 58; of the word Liver, xxvi. 313; observations on the derivation of the word Quickening, xxvii. 448; on adjectives ending in *ive*, xxxiii. 487; the advantages of etymological knowledge, 489; of the small-pox, xxxiv. 121.

EUCOMIS NANA, error corrected in regard to the description of that plant, xxix. 260.

EUDIOMETER, on the advantage to medicine from the use of the, i. 396; experiments made at Martinique with the, ii. 386; an excellent one in phosphorus, 389; description of Mr. Davy's, vi. 10; experiments with the, 12; importance of observations made with the, 203, 359; remarks and experiments with the several kinds of the, 376; experiments on the atmosphere of Philadelphia with the, vii. 122; account of that of Fortana, xxix. 403.

EUGENIA ZEALANICA, a plant of the order of myrti described, xxv. 549; its germen contains sixteen ovala, ibid.

EUNUCHS, on the formation of the larynx in, xii. 575.

EUONYMUS, or spindle-tree, botanical description of the, xii. 222; its medicinal properties, 223.

EUPATORIUM, or agrimony, botanical description of, xix. 262; a violent emetic and cathartic, ibid.; efficacious in ulcers, ibid.; cases of hernia cured by, xxiv. 517; the eupatorium perfoliatum cultivated early in England, xxvii. 429; the eupatorium cannabinum supposed to be the basis of the eau medicinale, ibid.; botanical observations on the eupatorium perfoliatum as it grows in America, 449; its powerful effects in the cure of fever, 450; and the ague, 451; one of the oldest American plants brought into England, xxviii. 15; its powers as a tonic and febrifuge, 476.

EUPHORBIUM, description of the, xvi. 75; its purgative qualities, ibid.; botanical description of the several kinds of the, xxv. 94, 372; analysis of the gums exuded by, 324; description of the resin of, 325; its component parts, 326; its efficacy in gout, xxvi. 200; on its medicinal properties, xxvii. 380; account of a new species, xxix. 429; analysis of one kind of, xxxii. 434.

EUPHRASIA, or eyebright, botanical description of the, xviii. 169; its virtue in strengthening vision, 170; remarkable instances of its beneficial effects in weak sight, xxiii. 104; observations on its utility in diseased vision, xxv. 71.

EUPHRATES, prevalence of epidemics on the shores of the, xix 114; the weeping willow first brought into England from that river, xxvi. 473.

EUROPE, observations on the inhabitants of, i. 405, 407; on the inflammatory diseases peculiar to, iii. 250; on the principal productions of, v. 371; prowess of scientific discovery in, vi. 408; odorous smell of some of the southern nations of, 443; physiological observations on its inhabitants, viii. 468; directions to Europeans who visit warm climates, x. 83; xi. 203; on the plants of Europe compared with those of tropical countries, xvi. 95; estimation of the female character in, xviii. 180; on the atmosphere of, xx. 6; on the diseases incidental to its inhabitants, xxi. 71; the highest tracts of land in, xxvi. 11; on the variations of climate in, xxx. 137.

EURYALE FEROX, a curious plant cultivated in China, described, xxvii. 525.

EUSTACHIAN TUBE, on the increased irritability of the, ii. 231; effects of galvanism of the, vii. 243; anatomical account of the, xviii. 6; deafness cured by injecting it, xxxiii. 400.

EUSTACHIUS, Bartholomew, account of his works, ii. 65; his discovery of the stapes, 160; his defence of Galen on the foramen of the vertebræ, 162; on the muscles of the ear, 164; his discovery of the stylo hyoideus, 165; united the hypogastric veins with those of the bladder, 167; his correction of an error concerning the aorta, 460; on the origin of the arteria ethmoidea, 461; not acquainted with the division of the humoral artery, ibid.; on the arteries and veins, ibid.; discovers the thoracic duct, 463; on the glands above the kidneys, iii. 76; on the ureters, 77; his error on the mediastrium, ibid.; on the organs of generation, 156; first described the sphincter vaginæ, 157; the first who truly described the human uterus, 159; on the human fœtus, 161; his figures of the brain, 258; his explanation of the optic nerves, 356, 361; on the facial nerve, 362; on the nerves of the voice, iv. 340; his explanation of the origin of the cervical nerves, 342; on the intercostal nerve, 343.

EUSTIS, Dr. on the uses of cold air and water in fever, i. 18.

EVANS, Arise, an astrologer, said to have raised a spirit at Battersea, iii. 246.

———, Dr. J. his letters on the success of vaccine inoculation, ii. 311; good effects of alkaline salts in counteracting poison, iii. 535; his case of compound fracture of the leg, with a representation, 549; his case of fracture of the cranium, iv. 346; his case of dislocated shoulder, v. 521; his case of fracture of the occipital bone, with the loss of the cerebellum, xii. 15; on the advantage of the cooling treatment of burns and scalds, xvi. 461, 527; his case of compound fracture of the ankle joints, xviii. 162; additional cases on the beneficial effects of cold in burns and scalds, 208.

EVANS, Mr. his curious collection of Eastern plants, xxviii. 164, 165.

EVAPORATION, ice prepared by the means of, vi. 409; new instrument for facilitating, x. 479; how that of alcohol destroys inflammation, xxiv. 101; particular effects observed in, 102; method of conducting the process, 103; ice procured by, xxvii. 430, 520; on freezing water by it in vacuo, xxix. 173; on the cold produced by that of the sulphuret of carbon, xxx. 425; ice in the East Indies not formed by, xxxiii. 235.

EVEILLE, M. on specks in the eye, ii. 479; his account of a singular fœtus, 480; on the Cæsarean operation, 481; on a case of tetanus, ibid.

EVERINGHAM, Mr. his account of a general inoculation for the small-pox, and its effects on vaccination, xx. 258.

EVIDENCE, statement of that given before the House of Commons on the petition of Dr. Jenner, vii. 489; viii. 17, 141; explanation of that given by Dr. Pearson, viii. 156; observations on the same, 265; necessity of caution in giving medical evidence in courts, 283, 347; defence of that given by Mr. Cuff on the same, ix. 66; account of a French work on the different kinds of, 69; explanation of that of Dr. Heberden on vaccination, 95; caution respecting that on child murder, 449; examination of the evidence on the Jennerian discovery, 481; observations on that given on the trial of Charles Angus for murder, xxi. 336, 399, 426; xxii. 118.

EVIL, on the frequent occurrence of it after the small-pox, v. 293. See *Scrofula*.

EWART, Dr. on the treatment of cancer, xii. 76.

EWELL, Dr. on the benefit of sugar of lead in profuse hæmorrhage, xxii. 350.

EXANTHEMATA, benefit of mercury in chronic, iii. 585; a new salt efficacious in herpetic, iv. 470; the erysipelas reckoned among, v. 484; observations on a peculiar one, vi. 79; the petechia excluded from the number of, 233; observed in dolor facei, 243; description of the, 298; some constitutions unsusceptible of, 366; on the origin of, vii. 177; use of charcoal in herpetic, xii. 283; observations on the several kinds of, xviii. 90; attributed to insects, xxiv. 26; description of them, xxx. 414.

EXCITABILITY, on the action of various powers in producing, i. 126; on that of the whole body, 127; observations on the Brunonian doctrine of, i. 488; ii. 450; the phosphoric acid a stimulus to, vi. 92; great accumulation of it in typhus, vii. 321; on its cessation in the system, ix. 290; on the causes of it, x. 25, 252; on the definition of it by Brown, xvi. 184; use of cold bathing in rousing it, xix. 111; observations on the preceding article, 304; different names for that property, xxi. 199.

EXCITEMENT, account of the Brunonian sense of that term, i. 125; tabular view of the degrees of it, 129; necessity of it to health, 132; observations on that doctrine, i. 488; ii. 450; vindication of the Brunonian doctrine, iii. 48; on the influence of sounds in producing it, 187; pain successful in counteracting the effects of opium, 574; on the medical practice in regard to, vi. 173; observations on an excess of it, 257; dysentery cured by it, xi. 516; on the definition of the term as used by Brown and his followers, xvi. 184; on that of the thyroid gland, 207; on the means of diminishing it, xix. 304; diseased liver cured by action of mercury, xxxi. 447.

EXCOECARIA AGALLOCHA, (or aloe,) botanical description of it, with a coloured representation, ii. 293; account of the aloe wood, 295.

EXCORIATIONS, the external application of flowers of zinc serviceable in healing, xi. 93; observations on those of the glans of the prepuce, xiii. 386; remedy for those behind the ears, xxv. 498; treatment of superficial ones, xxxiii. 387; benefit of starch or hair-powder in, xxxiv. 25.

EXCREMENT, observations on that of fowls, i. 505; odorous quality of that of the crocodile, iii. 68; the honey dew proved to be that of insects, xii. 229; experiments on that of butterflies, xxxii. 435.

EXCRESCENCES, on those formed on wild rose-bushes, iv. 377; observations on venereal ones, x. 179; case of excrescence on the scrotum, xii. 204; on extracting the fungous ones of the cerebrum or its membranes, xvi. 149; on those of the cornea, xx. 186; sometimes hereditary, xxi. 330; on the hæmorrhoidal excrescence, xxiv. 420; farther observations on the same, xxxiii. 238.

EXERCISE, caution in regard to it after taking emetics for the cramp in the stomach, i. 50; recommended to students, 260; advice to aged persons in regard to it, 494; its good effects against disease, iii. 298; on the impropriety of violent exercise, v. 297; beneficial in consumption, viii. 242; on the training of persons who are employed in athletic exercises, xiii. 532; that of the mind as necessary as that of the body, xiv. 277; on the degree to which it should be carried in dyspepsia, xv. 273; its effects on the body and mind, xvi. 207; cautions to those who use violent exercise, 383; how far serviceable in consumption, xxi. 327; farther observations on the necessity of abstaining from it in such cases, xxii. 89; want of exercise no cause of fever, 229.

EXFOLIATION, curious case of one, with an engraving, v. 497; on the cuticle, vi. 298; case arising from swallowing pins, vii. 17; instrument for extracting pieces of bone, vii. 554; whether the bones of the great extremities are exfoliated after amputation, xxiii. 456; case of exfoliation of the parietal bones, xxiv. 274; the effect of art as well as of nature, xxv. 488.

EXETER, account of an epidemic in that city and neighbourhood, ix. 505; progress of the influenza there, x. 135, 311; account of the black assizes at, xviii. 89; trial there of some persons for inoculating the small-pox, xxxiv. 168.

EXHALATIONS, observations on the noxious effects of them in the West Indies, i. 139; on the odour exhaled from the body, 243; effect of calcareous soils in counteracting, 254; on those of the human body, iv. 9; effects of poisonous ones, 273; on the odours exhaled by living animals, vi. 339, 439; effects of camphorised, 444; proved to induce fever, 468; intermittents caused by them, vii. 377; how productive of epidemics, x. 139; the deleterious effects of those of opium, xii. 42; pestilential consequences of them, xvii. 99; soporific effects of the odour of saffron, xviii. 288; on the nature of morbific ones, xxiii. 350; inquiry into the effects of putrid animal substances, xxiv. 423; on the exhalations of the human skin, xxviii. 161; deleterious effects of those of marshes, xxx. 16; on those which occasion intermittents, 159; infectious nature of those produced by cutaneous eruptions, xxxiv. 98; contagion occasioned by the exhalations of ulcers, 403.

EXOMPHALOS, or hernia umbilicalis, method of treating it, xx. 312; mode of cure by pressure no new discovery, 406; account of the operation for it in an aged person, 407; description of a new truss for it, xxv. 310; extraordinary instance of it, xxvi. 40.

EXOSTOSIS, instance of one cured by opiate friction, iii. 168.

EXOTICS, see *Plants.*

EXPECTORATION, necessity of promoting it in children, vii. 288: benefit of veronica in exciting it, xi. 371; efficacy of garlic in promoting, xiv. 65; the colchicum a powerful expectorant, 169; case of hepatitis followed by profuse bilious expectoration, 469; observations on expectorated matter, xxiii. 214, 223; on the want of a criterion by which to distinguish pus from mucus, xxiv. 10; observations and experiments on expectorated pus, xxv. 326, 342.

EXPERIMENTS, account of those which were instituted on the discovery of vaccination, i. 1, 10; on the analysis of atmospherical air, 27; on the cultivation of Indian corn, 47; on the artificial impregnation of animals, ibid.; account of those of Galvani, 53, 55; to try the benefit of medicated frictions, 71; on distilling animal substances, 85; on the rhus glabrum, or common sumach, 108; on a new species of cantharis, 142; on the temperature of the atmosphere necessary for the growth of plants, 161; for converting boiling water into vapour, 162; for producing detonating fulminations, 195; for producing artificial cold, 196; on vegetable fermentation, 200; on staining wood, 201; on the acid properties of tungsten, 239; observations on those of Mayow, 249; on the elements of plants, 263, 268; on atmospheric air, 299; on Canton's phosphorus, ibid.; on the temperature of the electric fluid, ibid.; on the separation of carbon from phosphorus, 300; on the sulphuric acid, ibid.; on sulphat of silex, 301; on the unguentum hydrargyri, 359; how to conduct those on plants, 379; on ascertaining the purity of the air, 397; on the nature of cancerous matter, 431, 435; directions for conducting them on vegetables, 495, 498; on the excrement of fowls, 505; on the red oxyd of iron, ibid.; on the black chalk of Bayreuth, 506; on the external use of tartarized antimony, ii. 35, 74; on animal substances treated with nitric acid, 76; on the inflammation of the ethers by acids, 77; on the decomposition of muriatic acid, 78; on the respiration of frogs and fish, 79; on the oxalic acid and lime, ibid.; on the muriatic and sulphuric acid, 81; on vegetable acids, ibid.; on the muriat of mercury, 82; on the martial flowers of ammoniac, ibid.; on fulminating substances, 185; on the non-oxygenability of sulphuric acid, 186; on the solution of mercury in nitric acid, 187; extraction of sugar from beet-root, 188; account of some instituted to ascertain the nature of the vaccine disease, 213, 219; on certain sensations of the eye, 227; on the Moffat waters, 354; with the eudiometer at Martinique, 386; on the Bononian phosphorus, 388; on the muriatic acid, ibid.; on the luminous property of phosphorus, 389; on the nitrat of mercury, 390; to separate mineral alkali from the salts, ibid.; on the optic nerve, 479; on new articular cavities of the bones, 482; on a new species of salt, iii. 82; on nitric ether, 86; on the oxyd of tungsten, 87; on the caustic potass, 88; on rendering odorous effluvia visible, 89; on the analysis of snow water, 90; on the extraction of soda from marine salt, ibid.; on lead spoons, ibid.; on the antiseptic property of beer, ibid.; on the petals of flowers, 91; on pulverized hæmatites, ibid.; on muriated acid and the earths, ibid.; on lime in fixed vegetable alkali, ibid.; on cheese, 166; on the separation of soda from marine salt, 169; on muriat of barytes, 170; the acetic and acetous acids, 172; on a new metallic acid, 173; on the extractive principle of vegetables, ibid.; on the vegetation of plants, 175; on different kinds of ink, ibid.; for producing acetous ether without mineral acid, 269; to find a substitute for coffee, 270; on the doctrine of animal life, 272; observations on those of Mayow, 342; method of analizing vegetables, 371; for obtaining carbon by decomposing the carbonat of lime by phosphorus, 374; on nitrous fumigation, 431; for obtaining sugar from vegetable substances, 582; on the honey-stone, 583; on the blood vessels of living and dead bodies, 587; account of those of Mr. Charles Darwin for a criterion between mucilaginous and purulent matter, iv. 49, 108, 203; to determine the quantity of fixed air in that of the atmosphere, 83; on the illumination of rotten wood, ibid.; on the germination of garden cresses, 84; on tellurium, ibid.; on the emphysematous humours, ibid.; account of some performed with the galvanic apparatus of Volta, 115, 126; for obtaining acid of tartar, 181; on the liquor amnii of a cow, 185; analysis of azote, ibid.; on a new earth in Sweden, ibid.; shewing that coal is a compound substance, ibid.; ascertaining iron particles in the blood, 186; on the division of the vertebræ, 190; to obtain sugar from indigenous plants, 244, 347; on the nitrous oxide, 265; on nitric acid, 266; on respiration, 274; for obtaining sugar and brandy from beet-root, 276; on the respiration of hydrocarbonate, 360, 451; on the sugar of the beet-root, 366; on the mildew and its acid, 372; on the crystallization of platina, 373; on the decomposition of red lead, 374; on the blue chalcedony of Siberia, ibid.; on the common kali, 375; on the properties of charcoal, ibid.; for purifying nitric acid, 376; on the tulip tree, ibid.; on a volcanic fossil of Iceland, 377; on the waters of Aix, in Provence, 458; on the origin of the vaccine virus, 465; on a new salt, 466; on urinary stones and gravel, 467; on absorption, 468; for curing the venereal disease by oxygen, 469; with the rock crystals of Switzerland, ibid.; on the medicinal barks, v. 33; on the anti-venereal power of the acids, 71; on the virtue of citric acid, 75; on houseleek, 198; on the glass of antimony, 199; on caloric, ibid.; on the chemical analysis of vegetables, 361, 467; on preparing ice vinegar, 395; on the veins of horses, 399; on elm trees, 492; with phosphoric acid, 562; on the illumination of rotten wood, 583; galvanic ones with saltpetre, 587; on the air of London, vi. 10; on the atmosphere at Bristol, 12; on the internal use of phosphoric acid, 92; on the windpipe in drowned persons, 94; on inoculating dogs with cow-pox, 95; on the absorbents of a horse, 139; on a new earth found in Sweden, 188; analysis of the honey-stone, 189; for ascertaining the quantity of carbon in the blood, 192; on the nature of chyle, 200; on land and sea air, 203, 359; account of Galvanic ones, 209; on the Kilburn waters, 242; analysis of cork, 276; on vaccine virus in hot weather, 327; on the Kilburn waters, 333; for the analysis of minerals, 373; to ascertain the influence of light on phosphorus, 374; on water in fossils, 375; in eudiometry, 376; on the solution of phosphorus in oil, 378; on the colouring part of the blood, 469; on the earth found in the Saxon beryl, 473; on Hahneman's nostrum for scarlet fever, 475; on the muriatic acid, ibid.; on the ear wax, ibid.; to determine the proportion of phosphoric acid and lime in bones, 476; on the atmosphere of marshes, 493; on the vitality of the blood, 513; on the Kilburn waters, 516; on James's powder, 518; on urinary stones and gravel, 527; vii. 60; account of some, to ascertain the quantity of earth in corn, vi. 568; arsenic acid in a fossil, 570; galvanic ones on frogs, vii. 71; to ascertain the origin of amber, 92; on the atmosphere of marshes, 113; on the modus operandi of opium, 124; on camphire, 129; with the cow-pox, 169; with nitric acid, 239; with muriatic acid, 240; details of galvanic, 242; on opium, 341; on the action of phosphorus in different gases, 374; relating to the pile of Volta, 381; on bituminous wood, ibid.; on the germination of seeds, 470; on the constituent parts of urine, 474; for preserving water sweet in long voyages, 477; on the contact of metals, 478; to ascertain whether fluids are conductors of caloric, 480; on metallic combinations of muriatic acid in its different states, 546; on the movement of odorous substances on water, 573; on emetic tartar, viii. 92; effects of carbonous gas on living animals, 189; on fulminating mercury, 190; on sheep in differents parts of Lincolnshire, 227; for the cure of diseases by galvanism, 250, 315; proving that the small-pox is common to men and brutes, 271; on the action of opium, 325, 387; on the combinations of the earths, 474; on the bulbs of the hyacinth, 475; on the urine of horses, 476; on electrical phænomena, 477; on the decoloration of vegetable liquors, 478; on the affinity of the earths, 479; on a blue oxydated iron, ibid.; on the nature of animal heat, 503; made on animals with hydrogenous sulphurated gas, 522; galvanic ones for medicinal purposes, 524; for purifying the atmosphere, 529; to ascertain the benefit of galvanism in rheumatism, 552; on phosphoric ether, 569; for distinguishing the two electric fluids, 572; proving that light and heat are not the same, ibid.; on gold and platina, 573; on atmospheric air, ibid.; on cinnabar, ibid.; on the red oxyd of mercury, ibid.; on the coccinella as a remedy in the tooth-ache, 575; on mercurial salts, ix. 9; medical ones on the saccharine matter in diabetes, 12; on alcohol, 43; on frogs with the same, 45; on the matter of the cow-pox, 67; on the sebacic acid, 78; on the juice of the papaya, 79; on the mild sublimate of mercury, 81; proposal for ascertaining the cause and effect of inflammations, 91; analysis of the boracites, 92; on the effect of galvanism in diseases,

131; on galvanic contractions excited upon cold-blooded animals, 146, 235; galvanic ones on the body of a malefactor, 195; on the humours of the eye, 196; on the putrefaction of flesh, 219; on urinary stones and calculi, 242; on the phosphuret of lime, 290; observations on those of galvanism, ibid.; on the sensibility of plants, 291; on the medical effects of phosphorus, 299; on the urine, 349; account of those performed by Aldini on the body of a criminal, 382; on the chemical analysis of opium, 398; advantages arising from those on galvanism, 445; on those of professor Aldini, 446; on the cow-pox, 453; on the vaccination of sheep, 455; on the gold ores, 473; on frogs, 487; on the materials of calculi and animal concretions, with a coloured plate, 489, 499; phosphat of lime discovered in corn, 584; discovery of a new metal called palladium, ibid.; on the cause of hunger and thirst, x. 22; on the causes of irritability and excitability, 25; on the analysis of opium, 42; results of them, 44; on the muscles of animals, 53; proving that the oxyds formed on the surface of the metallic disks do not impede the action, 63; on muriatic ether, 94; on cobaltic acid, ibid.; on metallic cobalt, 95; on the distilled waters of inodorous plants, ibid.; prussic acid in the aqua laurocerasus, ibid.; on the irritability of the fibrine, 253; remarks on those of Mr. Cruickshank on charcoal, 273; on stony concretions coughed up from the lungs, 327; on the application of galvanism for medical purposes, 330; with matter of the goat-pock, 342; on the cortex salicis latifoliæ, 374; on a malefactor, 383; on prussic acid, 479; on the effects of arsenic in a metallic state, 575; on the body of a decapitated person, 576; remarkable ones on the body of a criminal, xi. 94; curious effect of emetic tartar injected into a vein, 190; on the febrifuge principle of cinchona, 215; to shew that cinchona does not contain gelatine, 266; on gum-kino, 276; on the new earth said to be found in the Saxon beryl, 285; on the oxydation of iron and steel, 286; on arsenic in a metallic state, 287; on the animal fluids, ibid.; on nitric acid, 288; on the solution of platina, ibid.; a new vegetable salt, ibid.; medical use of the oxygenated muriatic acid, 292; on the urine of diabetic patients, 338; on corrosive sublimate, 340; analysis of meteorical stones, 352, 354, 355; for the preservation of dead bodies from putrefaction, 358; on the intestines of a dog, 375; new method of preparing arsenic acid, 382; on muriated barytes, ibid.; on fixed vegetable alkaline salts, 404; on vinous fermentation, 411; on arseniated hydrogen gas, 422; on lac sulphuris, 437; on the roots of orchis, 442; new substances found in platina, 479; on preparing the tartrite of antimony, 497; analysis of cinchona, 564; on the effects of lead in water, 571; on opium, xii. 39, 117; analysis of a new fossil found in Sweden, 69; on a new vegetable acid in the white mulberry, 92; effects of vaccination on sheep for the cure of the scab, 93; on emetic tartar, 189; to ascertain the security of vaccination, 264; on the use of charcoal in purifying liquors, 283; on the same in exanthemata, ibid.; on the preparation of broth from bones, 350; analysis of the same, 353; on gallic acid, 382; on the efficacy of muriat of soda against worms, 383; for ascertaining the strength of winds, ibid.; on the febrifuge substance in bark, 430; on the efficacy of animal jelly in fever, 431; on the security of vaccination, 473; on mercurius dulcis, 574; on the decomposition of sulphat of potash and sulphat of soda, 575; on disengaging heat by the compression of air, ibid.; statement of galvanic ones, xiii. 84; on a new metal in crude platina, 90; additional ones on galvanism, 372; on the black vomit of yellow fever, 378; on muriat and tartrit of antimony, 447; on the action of opium and tobacco, 563; water formed from hydrogen and oxygen by compression, 575; for the strengthening of the sight, xiv. 153; on the analysis of animal fluids, 263; to decompose water, 272; on the sulphates of copper and the nitrate, 382; on naphtha aceti, 383; on the effects of mercury in preserving teeth, 479; on the bark of the service tree, or sorbus accuparia, 572; on cutaneous absorption, xv. 274; on animal fluids, 280; for the decomposition of the muriatic acid, 295; on the analysis of James's powder, ibid.; on guaiacum, ibid.; on artificial tanning, 296; on the torpedo, ibid.; on the solution of the metallic oxyds in kalis, ibid.; on the tartar of teeth, 372; on a new metal, 390; on the foul air in cisterns, 391; on divided arteries, 463; on the augmentation of the galvanic action, 487; on the precipitation of copper, 582; on wounded arteries, xvi. 88; on the effects of arsenic in resisting putrefaction, 95; on a new mineral found in Cornwall, ibid.; on the enamel of human teeth, ibid.; observations on some made to ascertain the origin of vaccine virus, 124; on the nature of gravelly and calculous concretions, 153, 221, 305, 497; on the efficacy of some anti-contagious fumigations, 190; on the internal temperature of the earth, 192; on the blood of living dogs, 199; on the formation of chyle, 200; to ascertain the process of digestion, 292; on the effects of cold water on the pulse, 367; on the Bath waters, 383, 434; galvanic ones on vegetable infusions, 479; on native cinnabar, 575; on the gaseous oxyde of azote, xvii. 51; on the heat and pulsation of animals, 93; new portable blowpipe for chemical uses, ibid.; cruel ones on fish, 197; on the fermenting principle of albumen, 198; on distilled water, 295; on the iris, 404; on various ores in a silver mine, xviii. 193; on the Bononian phosphorus, 289; with frozen quicksilver at Hudson's Bay, ibid.; on corpses with the conductor of light, invented by Dr. Bozzini, 387; on the acid of sweat, urine, and milk, xix. 124; on important chemical agencies in electricity, 190; repetition of Dr. Davy's on decomposing the alkalies, 286; on oxalic acid, 287; on the effects of vegetation on atmospheric air, 371; on domesticated dormice, 383; on the illuminating properties of the oil of Chinese radish, 479; on the union of hydrogen with the metals, 574; to ascertain the action of the digitalis on the pulse, xx. 14; farther particulars of the decomposition of the alkalies, 95; on a new mineral found in the isle of Elba, 97; on the friction of fluids in exciting heat, ibid.; on respiration, 98; on muriatic ether, 125; on sulphurous mineral waters, 133; on the acid formed in indigestion, 193; on the Cheltenham waters, ibid.; account of some made with rhubarb to ascertain its effects on the urine and blood, 215; on the difference of electrical shocks, 287; on the production of fire by the compression of air, ibid.; on the conversion of iron into steel, 347; on the decomposition of the earths, 383; on the analysis of coffee, 384; method of conducting galvanic ones for medical purposes, 475; on the saccharine matter in Indian corn, 482; on the heat evolved by the arum cordifolium, 499; on the fecula of potatoes, 568; repetition of those on the decomposition of the alkalies, ibid.; on fruit trees, 569; to ascertain the degree and nature of animal heat, xxi. 69; on a thunder stone in Suffolk, 85; on the decomposition of the earths, 131; on the action of mineral poison on the stomach, 148; on the utility of platina for pendulums, 262; on potassium and galvanic combinations, 350; on calculi extracted from the bladder of a dog, 370; for detecting arsenic, 421; on Dr. James's powders, 438; on a mineral spring at Dudley, 439, 464; on several species of marble, ibid.; on the muscular contraction, 457, 464; on assafœtida, 476; on the identity of charcoal and hydrogen, 527; on the matter of fire, ibid.; abstract of Professor Davy's on the decomposition of the alkalies, xxii. 12; on the mineral waters of Cheltenham, 15; on the fœtid gums, 34; on the changes produced in the atmosphere by respiration and vegetation, 48; on the different kinds of calculi, 49; on the gelatine of the blood, 74; new method of detecting arsenic, 87; on a species of polypody, ibid.; on the action of some vegetables upon the spinal marrow, 96; on the angustura bark, 144; on the separation and transfer of the chemical agents, 174; on sulphuric ether, ibid.; on the rhododendron ponticum, 261; with venereal virus, 312; on the decomposition of water, 315; acid obtained from ginger, 349; on opium, 351; on copal, ibid.; on gums, 358; on sulphuric ether, 362; on the digestive quality of meal, 403; simple method of filtration, 439; on the pyrolignite of iron, ibid.; on preserving grapes, 440; on iron in the blood, 449; to produce substitutes for sugar, 525; on rabid animals, xxiii. 41; on carbonate of potash, 45; on the poison of champignons, 70; for purifying the air in large halls, theatres, and hospitals, 84; on colours found at Pompeii, ibid.; on leeches, 212; on expectorated matter, 214; on fluoric acid, 293; on the effects of heat upon the animal economy, 301; on the vesicating properties of the potatoe fly, 323; on the foot of the living horse, 338; on the foot of the horse, 339; on the nature of morbific exhalations, 350; on the pickle of violets, 351; on the effects of arsenic, 384; on spontaneous phosphorescent substances, 387; for detecting spurious glass of antimony, 439; with a new test for arsenic, 448; on the respiration of fishes, 451; on the action of poisonous substances, 454; on the mineral waters of Cheltenham, 509; on expectorated matter, xxiv. 10; analysis of the smut of wheat, 37; on the poison of arsenic, 59; caution in regard to medical, 65; new test for arsenic, 145; on the effects of the upas poison, 175; on different kinds of tea, 190, 265; on the effects of magnesia in preventing the formation of uric acid, 203; on the acetate of potash, 224; on an artificial stony substance, 316; on the combination of acids with animal and vegetable substances, 321; on the muriatic acid, 345; method of producing artificial cold, 347; on the combustion of charcoal, ibid.; on the eau medicinale, 352; on the poison of snakes, 400; on arsenical preparations, 403; on the horse-chesnut, 430; on the duration of muscular action, 485; on sea-sickness, 488; on the effects of riding, 491; on the organs of absorption, xxv. 45; on the barometer, 52, 149; for the improvement of grass lands, 54; on the combinations of oxymuriatic gas and oxygen, 83; decomposition of the grey oxide of tin, 84; on the acetate of Barytes, 85; on the influence of the moon, ibid.; on tobacco, 130, 134; on the white serum of human blood, 151, 156; analysis of the scammonies of Aleppo and Smyrna, 156, 157; on reddening litmus, 160; on the blood root, 166; on the metallic oxide, 180; on muscular motion, ibid.; on two minerals from Greenland, 181; effects produced by air and water on meat, 252; new mode of budding, 253; on cystic oxide, 254; on the existence of sugar in the blood of diabetic patients, 277; on the nature of vital heat, 278; on the inoculation of horses with various diseases, 281; on the cause of the redness of the blood, 282; for the formation of James's powders, 315, 317; analysis of gamboge, 320; of euphorbium, 324; observations and experiments on pus, 326; on the non-conducting powers of the thoracic duct and spleen, 361; analysis of zeolite, ibid.; effects of vegetable poisons on animals, ibid.; on a gaseous combination of oxymuriatic gas and oxygen, 362; on the relative powers of different galvanic piles, ibid.; on muriate of mercury, 363; for giving red colour to glass, ibid.; on luminous animals, 409, 512; analysis of calculous matter, 446; on the manner in which plants shoot forth their radicles, 452; on the rocks of the isle of Arran, 453; on a salt produced by the combination of phosphoric acid and potass, 456; on the fulminating powders of mercury and silver, 457; on sensation, 470, 474; on the decomposition of vegetable and animal substances by heat, 508; on the preparation of acetate of potash, 518; on the sensible perspiration of fraxinella, 520: analysis of a meteoric stone which fell in Ireland, 545; on the debility and diseases of trees, xxvi. 8; new ones on the metals of the fixed alkalies, 18; on manganese, 19; on salt, ibid.; on magnesia, 20; influence of the brain on the action of the heart, and in generating animal heat, 59, 66; on the circulation of the blood, 67; on the aorta descendens of dogs, 83; on the effects of caloric, 89; on sensation, 91; on the different modes in which death is occasioned by vegetable poisons, 129; on wounds made in the cavity of the body, 167; on the alcohol of wine, 168; on the Iceland crystal, ibid.; on the theory of vegetation, 170; on the eau medicinale, 185; on the same, 225; on atropa belladonna in defective vision, 247; process of making sulphate of magnesia, 251; on the poison of the upas, 252; on the poisonous quality of the belladonna, ibid.; effect of different gases injected into the blood vessels, ibid.; process for dissolving elastic gum, 264; observations on those of Mr. Brodie in regard to animal heat, 278; on a combination of oximuriatic gas and oxigen gas, 298; chemical analysis of sodalite, 303; with cinchona, 329, 331; analysis of the cactus coccinillifer, 342; on the circulation of the blood in inflammation, 354; elastic gum dissolved in ether, 382; on common water, 395, 397; on the injection of the gases into the venous system, 406; with leeches, 415; on worms, 454; non-existence of sugar in diabetic blood, 462; farther observations and experiments on the same, 467; on the value of them in medical science, xxvii. 2; effect of poisons on the tongue and alimentary canal, 3, 12; cruelty of experiments on living animals, 7; on the passage of fluids in the human body, 13; on oxymuriatic gas, 15; on the properties of the atmosphere, 16; on the effects of vegetation, 17; on wounded intestines, 57, 64, 129; on the nature of the blood, 73, 168; on a vegetable wax from Brazil, ibid.; on cantharides, 126; on camphoric acid, 127; on Benzoic acid, 128; on certain preparations of gold, 156, 158; on the relative state of the barometer and thermometer on Skiddaw, 170; on the composition of writing inks, 224; on the influence of cinchonin upon different barks, 295, 306; on the seeds of plants, 310; on calcareous bitter, and iron spar, 340; combinations of metals with chlorine and oxygen, ibid.; proving that young animals may live without respiring, 375; on worms, 376; on luminous animals, 377; on eels, ibid.; on ulcers in the feet of sheep, 379; on chemical and electrical attractions, 403; sugar extracted from beet-root, 404; on the gas of Prussic acid, ibid.; on the triple salts, 405; on effects produced by larvæ on the arnica montana, 407; on the anti-contagious properties of oxygenated muriatic acid, 408; on the foot of the living horse, 425; on the action of poisons in the animal system, 427; to procure ice by evaporation, 430, 520; on the strength of men and horses in moving machines, 488, 494; on arsenic, 518; on a mineral spring in the Isle of Wight, xxviii. 22; on dropsical fluids, 62; on the urine in diabetes, 63; on detecting arsenic, 66; another process for the same, 70; on the chalybeate at Sandrocks, in the Isle of Wight, 72; on the salt springs at Droitwich, 75; on sponges, ibid.; on metallurgy, 77; additional ones on the action of poison, 115; on soporifics from lettuce, 130, 133, 135; on vegetable wax, 136; on silicated fluoric gas, 157; on combinations of phosphorus and sulphur, ibid.; on sulphuric acid, ibid.; on stratified rocks, ibid.; prize questions for, 161; on vegetable poisons, 179; on the test for arsenic, 187; on a gaseous compound of carbonic oxide and chlorine, 195; analysis of deadly nightshade, 205; experimental examination of the Pharmacopœia Londinensis, 226; on bister and other substances in the distillation of wood, 235; wood converted into coal, 236; on a new solution of ferrum tartarisatum, 244; remarks on the arsenical test, 284; on the process of petrifaction, 305; on the mechanical power of light, 334; chemical researches on the blood and other animal fluids, 386; on the composition of chyle, 387; analysis of lymph, 390; analysis of the serum of blood, 391; on the coagulum of blood, 393; on the colouring matter of the blood, 394; effects of acids on the colouring matter, 395; effects of alkalies on the same, 397; remarks on the experimental details on the blood, 400; on the expansibility of the air, 420; for separating the alkaline salt from the acid, 425; for the application of pyrolignous acid to tanning, 427; on the constitution of water, 436; explanation on the arsenical test, 469; analysis of the urine of the ostrich, 470; method of obtaining artificial blood, ibid.; analysis of a rose-coloured substance found in urine, 471; of the lymph in the ventricles of the brain, 472; on the anti-syphilitic tincture of Besnard, 473; on the polarity of light, 512; on spontaneous combustion, 514; on those for detecting arsenic, xxix. 10, 46; on the physical constitution of water, 17; on maniacal subjects, 49; on the influence of the brain in generating animal heat, 141, 151; on a resinous substance from Sicily, 172; on freezing water by evaporation, 173; on alcohol, ibid.; on arsenic, ibid.; on malic acid, ibid.; a new cleavage found in calcareous spar, 174; on metallic veins of the earth, 247; for refining sugar, 249; on tea, 338; account of those of Mr. Cavendish on factitious airs, 399; on mineral waters by the same, 402; on the phenomena of freezing, 404; on air, ibid.; electrical ones of Mr. Cavendish, 406; on the density of the earth, 407; on some new properties of light, 426; on the narwal, 427; method of obtaining fine wire, 428; on vegetables found in some minerals, 430; on the freezing of alcohol, 431; on the production of animal heat, 444; on the liquid gum from Botany-Bay, 474, 480; on the eyes of birds, 481; on compositions for extinguishing fire, 493; on the black matter in the glands of the lungs, 506; on fossils found at Brentford, 507; on the formation of fat in the intestines, ibid.; on the tusks of the narwal, 508; on the alcohol of sulphur, 509; on peculiarities in the structure of seeds, ibid.; on the cotyledons of grasses, 510; analysis of arragonite, ibid.; on the quantity of heat for the consumption of substances, 512; on the heat of boiling water, 513; on the capacity of oxygen gas, carbonic acid gas, and hydrogen gas, for heat, 514; on the different actions of chemical phenomena under similar circumstances, 515; on oxymuriatic acid, ibid.; on the combination of hydrogen and carbon, 516; on ammoniacal gas, ibid.; on the solution of metals in acids, 517; on the absorbing power of charcoal, ibid.; on the distillation of iron pyrites, 518; on the temperature of animals, ibid.; on the calculi in the gall bladder, 519; on the daphne alpina, ibid.; analysis of fossil teeth, ibid.; on fungin, 520; analysis of various fungi, 521; on new salts of lead, 523; on the colouring matter of blood, xxx. 9; on biliary matter, 11; analysis of bile, 12; on the product and qualities of grasses, 13; analysis of the cerebral matter of man and other animals, 55, 68; on the alcohol of sulphur, 79; on combinations of phosphorus and sulphur, 83; composition of azote, 85; on pure alumina, ibid.; on the injection of blood vessels, 90; on the digestive powers of animals, 108; on the bark of the cocoloba uvifera, 168; on the effects of vomiting, 209, 216; on Godorneau's powder, 222; on the animal fluids, 233; analysis of urine, 234; on the use of magnesia in urinary calculus, 249, 324; on the phenomena of electricity, ibid.; on the squalus maximus, 250; on the sap of plants, 251; on the structure of the organs of fructification in plants, 253; on a calculus found in the urethra of a hog, 256; on the hydrosulphurets, ibid.; spontaneous generation of ammonia, 260; on the chalybeate spring at Melksham, 300, 383; on the heat evolved during inflammation, 323; on a resinous substance dug up at Highgate, 343; on the antimoniacal acids, 344; on the acid of tin, ibid.; on the acid of tellurium, ibid.; analysis of the sulphite of copper, 345; method of obtaining intense heat, 346; additional ones on alcohol in fermented liquors, 392; on radical heat, 396; determination of the specific heat of different gases, 399; on the new detonating compound of chlorine and azote, 424; on the cold produced by the evaporation of sulphuret of carbon, 425; on the composition of fluor spar, ibid.; on the volatility of cerium, 426; action of azote on light, 427; on animals without vertebræ, 430; on corals, 431; on a new species of eels, 433; on bees, ibid.; made with gastric juice to ascertain its digestive power out of the stomach, 517; on the forms of articulation of the fore arm, ibid.; on the form of the sternum in birds, 518; on the anatomy of birds, ibid.; on grasshoppers, ibid.; on the gestation of the viper, ibid.; on vaccination, 519; preparation of the Porporino red, 523; new instance of combustion during construction, ibid.; on the formation of fat in the intestines, xxxi. 43; on cutaneous absorption, 80; on animal concretions, 81; on cataract performed at Greenwich Hospital, 82; on definite properties in chemical combinations, 85; on the colouring matter of the black bronchial glands, 128; description of a new scale for chemical experiments, 167; influence of the nervous system on muscular motion, 168; on the generative organs, 212; on a new gas extracted from sea-weed, 255; on the use of the epiglottis, 318; on fluorine and other objects of chemical investigation, 337; influence of the nerves on the secretions of the stomach, 338; account of some to determine the specific heat of arterial and venous blood, 339; on barilla, 346; on the principle of vitality, 419, 431; influence of the nerves on the stomach, 486; analysis of the bones of the spine in a case of mollites ossium, 498; analysis of animal fluids, 500; on weighing liquids, xxxii. 16; on respiration, 36, 49; on the principles of life, 56; method of oxidizing silver by the jatropha cureas, 74; on magnesian limestone near Dublin, 77; on the colchicum as a remedy in gout, 78; analysis of a calculous concretion, 80; analysis of a new bitumen, 81; on the ergot of rye, 93; on the influence of the temperature of the air in respiration, 132; on a biliary calculus, 169; on the influence of air in shut sacs, 208; analysis of the blood and animal fluids, 222; on the extrication of caloric during the coagulation of the blood, 223; on the production of animal heat by respiration, 233; influence of the nerves on the beating of the arteries, 248; on the virtues of charcoal as an antidote to poison, ibid.; solubility of white oxide of arsenic in water, 253; on digestion, 257; refutation of Dr. Hale on animal heat, 295; on the eyes of insects, 314, 400; on the effects of the upas, 343; on the action of poisons, 346; on earth-worms, 347; on iodine, ibid.; on the internal use of charcoal, 428; on the action of vomiting, 429; analysis of the euphorbia cyparissus, 434; analysis of the caoutchouc of Thibet, ibid.; analysis of Rocou, 435; on diabetic urine, ibid.; on the preservation of flesh in the gases, ibid.; on the formation of pyrophorus, 436; additional experiments on vomiting, 517; on those of M. Magendie illustrative of the same, 518; on the nutrition of the fœtus, 521; with a new diving machine, 522; charcoal an antidote to poison, 524; on combinations of chromic acid with different bases, xxxiii. 33; on barytes, 35; on the triple compounds of iodine and oxygen, 37; on acid compounds of iodine, 43; action of some compound gases on iodine, 46; on the urine and serum of the blood, 69; vindication of those of Mr. Brodie on the vital principle, 85; Dr. John Davy's experiments on animal heat, 113; Sir Humphrey Davy's on the combustion of the diamond and other carbonaceous substances, 121; on fluids in the pericardium, 139; on magnesia in human bones, 159; on some remarkable urine of a man in gonorrhœa, 160; on separating nitrous from sulphuric acid, 219; on carbonate of barytes, 220; on dew, 232; on carbonic acid gas in the lungs, 240; on semen, ibid.; on the accommodation of the eye to distance, 241; cruelty of experiments on living animals, 247, 401; on the principle of life, 309; mineralogical observations in Cornwall, 337; on the chameleon, 339; on bats, ibid.; metallization of charcoal, ibid.; on the respirating organs of insects, 397; on the action of the nerves in vomiting, 399; on poisons and their antidotes, 400; on the digestive process, 420; report on the experiments of M. Orfila on poisons, 471, 477; improved mode of making antimonium tartar, 484; easy mode of procuring potassium, xxxiv. 26; account of those of Dr. Black, 37, 40, 42; on the brain, 43; on the guinea worm, 57; on the action of acids on hyperoxymuriate of potash, 79; on the pulse, 80; on malic acid, 81; on double distillation from the same heat, 114; on the pulsation of the arteries, 163; on a new vegetable acid, 164; on the preparation of malic acid, 165; on the respiration of vermes in water, ibid.; on the mode of generation of the lamprey and myxine, 165; on the extra-vascular and vascular parts of animals, ibid.; galvanic ones with a large battery, ibid.; on the application of vegetable poisons to animals, 169; thermometrical experiments at Athens, 171; account of those of Mr. Tennant on the nature of the diamond, 209; on magnesia in limestone, 211; on germination, 212; on emery, ibid.; analysis of the powder of crude platina, ibid.; proving that gonorrhœa and a superficial ulcer arise from the same virus, 245; for artificial soda water, 292; improvement in preparing vegetable extracts, 293; on the brain, 309; on the nature of fat, 435; on a calculous found in a tumour, 437; on the effects of arsenic, 442; on carbonate of magnesia in calculous complaints, 445; on the constituent parts of the atmosphere, 469; on antimonium tartarisatum, 472; on the œsophagus, 524; on amputation, ibid.

EXPLOSION, account of a new invented lamp to prevent the dangerous effects of explosions in coal mines, xxx. 82; account of a dreadful one in the Collingwood Main Colliery, 427.

EXPRESSION, observations on the anatomy of, xviii. 8.

EXTENSOR MUSCLES, case of paralysis of those of the hand and fingers, iii. 253; observations on that case, 254.

EXTENUATION, singular case of that of the leg, with the process of cure, ix. 231.

EXTON, Dr. his method of extracting the placenta, iv. 325.

EXTRACTIVE PRINCIPLE, inquiries on that of vegetables, iii. 173.

EXTRACTS, benefit of alcohol in the preservation of bitter, iii. 581; on the virtue of the extractum cardui benedicti, vi. 370; method of preparing extract of opium, xxii. 351; on the nature of pharmaceutic, xxxi. 517; improved method of making vegetable, xxxiv. 293.

EXTRAVASATION, that of the blood in considerable quantity, cured, vi. 92; the alleged cause of apoplexy, 549; objections to that opinion in a particular case, 550; explanation on the subject, 561; the cause of sanguineous apoplexy, vii. 74; the cause of the death of Mr. Cruickshank, 77; the general cause of apoplexy, 78; that doctrine controverted, 89; farther objections to it, 140, 152; defended, 279; the opinion qualified, 363; erroneous opinion in it corrected, 438; explanation of the term, 452; the doctrine vindicated, 458; exploded, viii. 69; questioned whether a cause or effect, 78; on the use of the term, 115; fatal in apoplexy, ix. 322; case of fissure of the cranium attended with, 244; cases of extravasation of blood upon the brain, with an engraving, xxiv. 89, 92, 95; case of spontaneous extravasation within the theca vertebralis, xxx. 167; case of extravasation of bile into the cavity of the abdomen, xxxii. 152; effects produced by it on the brain, xxxiv 49.

EXTREMITIES, on applying the tourniquet to restrain arterial hæmorrhages from the lower, i. 23; observations on swellings of the lower, vi. 51; benefit of galvanism in paralysis of the, viii. 258, 322; on the connexion of the arteries of the, ix. 178; on the inosculations of the lower joints of the, ibid.; utility of amputating those of the bones in caries of the joints, x. 557; consequences of fracture of the, xiv. 476; on contraction of the lower, xv. 326; case of enlarged lower extremity, with a plate, xxv. 400; case of paralysis of the lower, xxvi. 153; extraordinary enlargement of the right lower extremity, xxviii. 60; utility of diuretics in ulcers of the lower, 141; new mode of treating fractures of the, xxxii. 177; on the management of fractures of the, xxxiii. 164.

EXUDATION, that of lymph the occasion of serous apoplexy, vii. 74; on the application of the term in that disorder, 77; proved not to be the cause of apoplexy, 400; nor to have any fatal effects, ix. 322; case of the blood exuded by the cutaneous pores, xxxiii. 246.

EYE, on the disease called amaurosis in the, i. 24; an epidemic disease of the, 234; cautions to students for the preservation of their sight, 260; narcotics recommended in chronic inflammation of the, 284; the oil of cajeput serviceable in diseases of the, 285; appearances of it in fever, 478; appearance of it in hydrocephalus, 492; history of the discovery of the muscles of the, ii. 164; experiments and observations on certain sensations of the, 227; on one of the means by which it accommodates itself to different distances, 331; superstitious opinions on disorders of the, 368; on yellow specks in the, 479; child born with only one, ibid.; discovery of the organs of secretion and lachrymal ducts in the, iii. 78; on the tunica albuginea, 79; on rendering odorous effluvia visible to the, 89; anatomical discoveries of the, 356; effect of the extract of belladonna applied to the, 367; farther observations on the same, iv. 5; ointment for humid ophthalmia, 88; description of a new speculum for extracting cataract, with a drawing, 103; benefit of cold water in weakness of sight, 179; corrosive sublimate serviceable in amaurosis, 184; on inflammation of the, 358; projected treatise on diseases of the, 471; benefit of electricity in a case of effusion of blood into the chambers of the, v. 68; on the operation of light on the, 199; extraordinary case of tumour of the, 241; cause of the weak sight in children, 295; remarkable appearance of it in trismus, 325; on the use of the recti muscles of the, 326; effects of scrofula on the, 413; remarks on an orifice in the retina of the, 585; on yellow spots in the, 586; on the state of it in the plague, vi. 268; remarks on the paralysis of the, 275; benefit of the extractum hyosciami in weakness of sight, 276; extirpation of an extraordinary tumour of it, with an engraved representation, 289; on the use of belladonna in the extraction of cataract, 352; on the ophthalmia of Egypt, 357; remarkable complaints in the lids of the, 393; query on the extraordinary tumour of the, 461; benefit of opium in ophthalmia, vii. 60; effects of camphire and opium on the, 129; danger of exposing it to intense solar heat, 209; peculiar effects of some climates on the, ibid.; ocular inflammations produced by various causes, 210; benefit of topical bleeding in, 211; means of preserving the, 212; on removing motes in the, ibid.; benefit of scarification in complaints of the, ibid.; forms of collyria, 215; effects of galvanism on the, 242, 247; cases of cataract removed by inflammation, 405; effects of galvanism on the, 531, 533, 536; restoration of sight in a case of cataract, viii. 115; method of treating the Egyptian ophthalmia, 185; action of galvanism on the, 319; galvanism beneficial in amaurosis, 323; case of opacity of the cornea, 398; on leucoma, 399; on removing cataract by artificial inflammation, 548; on the ophthalmia of Egypt, 555; occasioned by lime, 556; method of treatment, 557; preservatives, 558; effects of galvanism in disorders of the, ix. 131; cases of amaurosis, 138, 142; observations on cataract, 152; analysis of the humours of the, 196; case of cataract, 198; operation for extirpating the globe of the, 200; on the cause of the ophthalmia of Egypt, 575; on a case of early cataract, x. 10; case of the cure of amblyopia, 58; cure of the paralysis of the eyelids, 60; description of instruments for extracting cataracts, with a plate, 65; the operation of couching recommended, 371; observations on various diseases of the, 561; on the puriform running of the lids of the, ibid.; on tracheasis, 562; on macula cornea, ibid.; ulcers of the cornea, 563; pterygium, ibid.; process of depressing the cataract, 564; on the membranous cataract, xi. 70; the globe of the eye considered as a dioptric machine, 87; success of electricity in amaurosis, 135; humours of the, 163; singular case of tumour in the eye, with an engraved representation, 197; appearances on dissection, 199; inflammations of the eyes after measles, 315; case of fistula lachrymalis, 551; the ophthalmia of Egypt contagious, xii. 79; particularly affected by psoriasis diffusa, 97; juice of the rupture-wort good for specks in the, 227; use of sponge in ophthalmia, 233; on the cause of inflammation in the, 407; on the treatment of it with astringents, 408; ceruse of lead useful in disorders of the, 471; dispensary proposed for diseases of the, 576; patronage of that design, xiii. 96; observations on the proper means for the preservation of the eyes, xiv. 11; remarkable case of blindness produced by a fright, 15; case of optical illusion from hysteria, 117; observations on vision, 152; benefit of cold application in scrofulous ophthalmia, 184; use of cold water in epidemic ophthalmia, 239; case of bony tumour extracted from the, 471; analysis of the same, ibid.; case of gutta serena relieved by electricity, 481; mezereon supposed to be efficacious in disorders of the, 544; on the division of the iris, with a case, xv. 4; remedy for inflammations of the, 68; remarks on the couching needle, 103; effects of galvanism in the cure of disorders of the, 151; inflammation of the eyes occasioned by debility, 194; benefit of the asarum in disorders of the, 268; sequel to the case of the division of the iris, xvi. 1; on inflammation of the iris, and efficacy of belladonna to prevent the obliteration of the pupil, 55; case of gutta serena in the, 58; cases of ophthalmia, 60, 62; mucilage of quince-seeds good for an inflamed, 284; benefit of celandine in opacities of the, 559; practical observations on the principal diseases of the, 562; benefit of tincture of opium in ophthalmia, 575; remedy for diseases of the, xvii. 74; on disorders of the, 79; case of extirpation of the, 187; effect of evacuating the aqueous humour in inflammation of the, 193; account of a newly discovered membrane in the human, 283; successful treatment of the fistula lacrymalis, 284; on the contagious nature of diseases of the, 316; on the varieties and consequences of ophthalmia, 379; observations on ophthalmia as a contagious disease, 385; memoir on the organization of the iris and on artificial pupils, with engravings, 403; on that of the horse, 413; on that of the cat, 414; that of the bear, 415; on the eyes of swans, 416; on the ophthalmia in the British army which returned from Egypt, 479; effects of the obliquity of the, 522; observations on the cause of squinting, 523; error in the account of the new discovered membrane in the, 527; virulent ophthalmia among the British soldiers, xviii. 12; peculiarity observed in the, 15; farther remarks on diseases of the, ibid.; method of treating them, 16; benefit of eyebright in strengthening the, 170; observations on the Egyptian ophthalmia, 474; queries on the epidemic ophthalmia, 493; account of an epidemic ophthalmia at Edinburgh, 578; the fumitory beneficial in disorders of the, xix. 72; on the modern operations for cataract, 101; case of cataract, 102; observations on cataracts, 104; observations on cataract and strabismus, 310; effects of wormwood on the, 356; efficacy of the actual cautery in obstinate disorders of the, 430; how the infectious ophthalmia may be affected by a moist atmosphere, 545; on the application of irritating substances to the, ibid.; observations on the ophthalmia at Edinburgh, 571; on the contagiousness of the Egyptian ophthalmia, xx. 10; case of purulent ophthalmia, 57; observations on that disease, 59; on the ophthalmia as it appeared at Edinburgh, 90; case of fistula lachrymalis, with reflections on the different modes of operating in that disease, 147; on the mortification of the cornea, 174; essays on the morbid anatomy of the human, 182; on the diseases of the cornea, 185; pustules on the cornea analogous to apthæ, 186; on the abscess of the cornea, ibid.; on the ossification of the cornea, 187; on the formation of specks in the, 188; cloudiness not to be confounded with specks, 189; frequency of ophthalmia at Edinburgh, 190; observations on a case of purulent ophthalmia, 237; on the sensibility of the inflamed cornea, 472; benefit of the extract of lead in disorders of the, 563; on making the incision of the cornea for extracting the cataract, xxi. 146; on the Egyptian ophthalmia and purulent ophthalmia in England, 164; ointment for the eyes, 165; diseases of the eyes hereditary, 232; announcement of a practical work on diseases of the, 263; observations on inflammation of the, 390; on the epidemic ophthalmia, xxii. 116; on local inflammation chiefly in diseases of the, 167; benefit of tincture of opium in disorders of the, 170; case of purulent ophthalmia, 252; account of an epidemic ophthalmia, 260; observations on some phenomena in vision, 307; account of an optical illusion, 311; benefit of galvanism in disorders of the, 320, 322; amaurosis cured by Cayenne pepper, 353; on the treatment of purulent ophthalmia, 364; origin of ophthalmia, 457; on the vibration of the iris, 468; on the spontaneous passage of the crystalline humour, 474; directions to be used in such cases, 478; benefit of Goulard's extract in ophthalmia, 523; case of ulcerated cornea, xxiii. 57; remarks on the purulent ophthalmia, 77; on the supposed venereal origin of the purulent ophthalmia, 79; case of hydropthalmia from a blow, 125; method of operating in that case, 126; remarks on the same, ibid.; on the purulent ophthalmia of infants, 154; benefit of hyoscyamus in inflammation of the eye after cataract, 501; observations on the fungus hæmatodes as it affects the eyeball, xxiv. 12; observations on the supposed contagiousness of the ophthalmia, 130; cases of ophthalmia cured by bark, 131, 133; observations on the use of cinchona in ophthalmia, 221; queries on the epidemical ophthalmia in the British army, 317; on the morbid sensibility of the eye, xxv. 67; causes of weakness of sight, 68; method of cure, 69; benefit of leeches in, 70; purgatives useful in such cases, ibid.; fomentations, 71; benefit of digitalis in those complaints, ibid.; peculiarities of individual cases, 72; on the proper choice of glasses, 73; observations on psoropthalmy, 74; danger of applying to empirics in disorders of the, 75; utility of the solution of zincum vitriolatum, 76; observations on collyria, 77; remarks on the inflammation of the eye, and mode of treating it, ibid.; improved method of treating the blear eye, 85; cause of the failure of the operation of couching, 148; account of the staphyloma or protrusion of the iris, 161; method of relief, 162; on the treatment of hydropthalmia, 163; account of a child born without any eyes, 277; case of fungus hæmatodes, 303; on two varieties of ophthalmia in the army, 357; on staphyllana pellucidum cornicum, 359; advantage of metallic oxyds in ophthalmia and psorophthalmia, 497; method of applying the same, 498; case of amaurosis, 510; observations on ophthalmia, and its consequences, 522; an inflammatory and contagious disease, 523; description of the several kinds of ophthalmia, 527; efficacy of bleeding in it, 530; opium a palliative remedy, 531; a syphilitic taint of the system one cause of the disorder, 532; on relapses of the complaint, 534; effect of the sand at Alexandria on the eye, 535; practical observations on the formation of an artificial pupil, 535; on the insensibility of the iris, 536; operations for extracting the soft and capsular cataracts, 542; practical observations on the diseases of the inner corner of the eye, xxvi. 227; method of treating the fistula lachrymalis, 228; description of the epiphora, or flow of tears, 230; remarks on the sacculi lachrymalis, ibid.; on the obstructed state of the ductus ad nasum, 233; the focal distance of the eye dependent on the contractability of the muscles, 247; benefit of the atropa belladonna in strengthening the sight, ibid.; its appearance in phthisis pulmonalis, xxvii. 38; in hydrocephalus, 39; in scrofulous diathesis, ibid.; in gout, ibid.; in epilepsy, 40; on a peculiarity in the convex form of the, 114; use of the couching needle in infants, 161; observations on tropical nyctalopia, or night blindness, 165; contagious blindness in sheep, 219; account of a posthumous work by Mr. Saunders on diseases of the eye, 229; on the inflammation of the conjunctiva in infants, 230; use of leeches in that case, 231; on inflammation of the iris, 232; benefit of the belladonna, 233; on the cure of the inversion of the upper eyelid by excision of the tarsus, ibid.; on the terminations of ophthalmia, 234; illustrations of changes in the structure of the eye, 235; on the structure of the eye in men and birds, 257; practical observations on cataract, and the operations for it, 318; difficulty of operations to remedy derangement of the iris, 323; on the congenital cataract, 335; convex glasses disapproved of, 336; on the operation in the capsula, 337; disorders of the eyes affected by changes of the moon, 402; case of erythema mercuriale, accompanied by an affection of the, 420; improvement in the treatment of the, xxviii. 20; two cases of gutta serena produced by cancerous affection, 28; on the influence of belladonna in producing a dilated condition of the pupil, 258; Mr. Saunders's account of his method of operating in cataract, 259; cases of successful operation in cataract, 261; on the suspension of the absorptive process of the eye, 263; case of wound in the cornea, 264; practical remarks on the instrument used for operating in different kinds of cataract, 358; description of a new couching needle, 359; manner of performing the operation, 361; peculiar influence of the belladonna, 363; cases of capsular cataract, 364; advantages of the iris knife, 365; description of that instrument, 366; method of operating with it, ibid.; remonstrance of Dr. Farre to Mr. Stevenson on his operation for cataract, 467; syllabus of lectures on the, 517; Mr. Adams on the origin of one of his instruments for the operation of cataract, xxix. 89; description of the two-edged needle for the solid cataract in children and adults, 91; method of conducting the operation, 92; cases of its success, 93; cases of Egyptian ophthalmia in the workhouse of St. Pancras, 302; benefit of tartarized antimony in that complaint, 304; practical observations on ectropium, or eversion of the eyelids, 312; on the formation of the eyelids, 314; operation for ectropium, 315; on contracted or obliterated pupil, 316; on the formation of artificial pupil, 318; new method of operation, 319; on syphilitic inflammation of the eye, 320; early operation in cataract recommended, 321; description of instruments for the operation in cataract, 322; on opacity in the posterior portion of the capsule of the crystalline lens, 323; description of the treatment, 324; on the symptoms of amaurosis, 327; how the eyes are affected in spotted fever, 331; account of a practical treatise on cataract, 420; description of the symptoms of cataract, ibid.; proximate and exciting causes of cataract, 421; several modes of treating it, ibid.; on the process of absorption, 422; utility of the belladonna in the same, ibid.; benefit of the hyoscyamus, 423; remarkable case of short-sightedness, 426; success of the operation in the complicated ectropium, 432; on the efficacy of antimony in ophthalmia, 452; description of an organ by which the eyes of birds are accommodated to different distances, 481; on the action of antimony in ophthalmia, xxx. 46; benefit of the nauseating practice, 47; case of congenital cataract, with observations on the means of artificially dilating the pupil, 68; discovery of the utility of belladonna in affections of the eye, 69; effects of the hyoscyamus on the sight, 71; on the ultimate vascularity of the eye, 93; composition of the cornea, ibid.; on the capsule of the lens, 94; description of the hyaloid membrane, ibid.; of the retina, 95; on the eye of the ox, ibid.; on the alleged utility of exciting nausea in diseases of the eye, 199; Indian method of operating for the cataract, 221; case of remitting ophthalmia, 237; anatomy of the eye of the shark, 250; case of periodical day blindness, 509; on the external application of belladonna to the eye for dilating the pupil, 511; the antimonial tartar inefficacious in ophthalmia, xxxi. 17; sore or blear eyes common among the Laplanders, 52; new operations for cataract, performed in Greenwich Hospital, 82; singular affection of the eyes, 147; vindication of the antimonial tartar in Egyptian ophthalmia, 189; on the mechanism by which the eye is exactly adapted to the impressing agent, 256; perspicuous description of its anatomy, 257; prevalence of epidemic ophthalmia, 264; extraordinary affection of the eyes in pemphigus, 267; efficacy of emetics in severe ophthalmia, 272; on the new operation for cataract, 295; official account of the operations performed at Greenwich Hospital for the cure of cataract and ophthalmia, 326, 335; review of a practical treatise on cataract, 336; farther observations on the use of antimonial tartar, 355; explanation concerning the operations at Greenwich, 382; effects of evacuating the aqueous humour in inflammation of the eyes, and some diseases of the cornea, 504; on the anatomy of insects, 515; on the broad-leaved tobacco, 519; on the bitter principle of nitric acid and vegetable substances, ibid.; on the causes of opacity, xxxii. 65; cataracts formed in utero, 66; on those of new born children, 67; how the capsule is affected in cataract, 70; on the fluid cataract, 73; the operation for the hard cataract, ibid.; practice of operating in cataract used by Celsus, 118; on the compound and simple eyes of insects, 314, 400, 500; terms for students at the infirmary for the, 347; effects of pemphigus in the eye-lids, 397; strictures on the official report of cases of cataract in Greenwich Hospital, 486; illiberality manifested in that report towards other practitioners, ibid.; the operator not entitled to the merit of having discovered a new mode of curing Egyptian ophthalmia, 487; that honour due only to Mr. Saunders, 490; on the simple or smooth eyes of insects, 503; description of nyctalopia or night-blindness, xxxiii. 63; operation for the artificial pupil, 71; on the mechanism by which the eye accommodates itself to different distances, 157; explanation respecting the operation for artificial pupil, 192; on the accommodation of the eye to distance, 243; case of divergent strabismus of the right eye, 303; observations on the black cataract, 311; cases of that complaint, 312; rare instance of it, 313; condition of the eye in it, 314; successfully treated, 315; special report of the Infirmary for diseases of the eye, on the fraudulent attempts to injure that institution and the memory of its founder, 327: on Mr. Adams's vindication of himself, and his pretensions, in the Edinburgh Journal, 332; on the muscæ volitantes of nervous persons, 411; the cataract seldom sudden in its appearance, 413; observations on cataract, 416; case of depressing rabid cataract, 417; on the use of vinous solution of opium in diseases of the, 428; case of amaurosis cured by electricity, 468; its peculiar appearance in the plague, 481; blindness produced by epilepsy, 516; benefit of liquid laudanum in ophthalmia, xxxiv. 10; case of the cure of black cataract, 85; amaurosis relieved by blisters, 425; mode of discovering the proper capsula of the crystallized lens, 453.

EYEBRIGHT, (*euphrasia,*) botanical description of the, xviii. 169; its use in strengthening weak eyes, 170; cases in confirmation of its virtue, xxiii. 104; how to be used, 107; on its supposed efficacy in weakness of sight, xxv. 71.

